# Comparative Analysis of AGPase Genes and Encoded Proteins in Eight Monocots and Three Dicots with Emphasis on Wheat

**DOI:** 10.3389/fpls.2017.00019

**Published:** 2017-01-24

**Authors:** Ritu Batra, Gautam Saripalli, Amita Mohan, Saurabh Gupta, Kulvinder S. Gill, Pritish K. Varadwaj, Harindra S. Balyan, Pushpendra K. Gupta

**Affiliations:** ^1^Bioinformatics Infrastructure Facility (BIF) Laboratory, Department of Genetics and Plant Breeding, Ch. Charan Singh UniversityMeerut, India; ^2^Molecular Biology Laboratory, Department of Genetics and Plant Breeding, Ch. Charan Singh UniversityMeerut, India; ^3^Department of Crop and Soil Sciences, Washington State UniversityPullman, WA, USA; ^4^Department of Bioinformatics, Indian Institute of Information Technology-AllahabadAllahabad, India

**Keywords:** AGPase, genes structure, ligand binding, molecular dynamics simulation, ADP_Glucose_PP domain, promoter analysis, expression analysis

## Abstract

ADP-glucose pyrophosphorylase (AGPase) is a heterotetrameric enzyme with two large subunits (LS) and two small subunits (SS). It plays a critical role in starch biosynthesis. We are reporting here detailed structure, function and evolution of the genes encoding the LS and the SS among monocots and dicots. “True” orthologs of maize *Sh2* (AGPase LS) and *Bt2* (AGPase SS) were identified in seven other monocots and three dicots; structure of the enzyme at protein level was also studied. Novel findings of the current study include the following: (*i*) at the DNA level, the genes controlling the SS are more conserved than those controlling the LS; the variation in both is mainly due to intron number, intron length and intron phase distribution; (*ii*) at protein level, the SS genes are more conserved relative to those for LS; (*iii*) “QTCL” motif present in SS showed evolutionary differences in AGPase belonging to wheat 7BS, *T. urartu*, rice and sorghum, while “LGGG” motif in LS was present in all species except *T. urartu* and chickpea; SS provides thermostability to AGPase, while LS is involved in regulation of AGPase activity; *(iv)* heterotetrameric structure of AGPase was predicted and analyzed in real time environment through molecular dynamics simulation for all the species; (*v*) several cis-acting regulatory elements were identified in the AGPase promoters with their possible role in regulating spatial and temporal expression (endosperm and leaf tissue) and also the expression, in response to abiotic stresses; and (*vi*) expression analysis revealed downregulation of both subunits under conditions of heat and drought stress. The results of the present study have allowed better understanding of structure and evolution of the genes and the encoded proteins and provided clues for exploitation of variability in these genes for engineering thermostable AGPase.

## Introduction

Among all major enzymes involved in starch biosynthesis in plants, ADP-glucose pyrophosphorylase (AGPase) is the first key regulatory and rate limiting enzyme (Smidansky et al., 2002; Tuncel and Okita, 2013). Using glucose-1-phosphate (G-1-P) and ATP as the sole precursor molecules, AGPase synthesizes ADP-glucose (ADP-G) moieties, which are the basic building blocks of starch (Soliman et al., 2014). This enzyme controls the amount of starch in cereal grain endosperm as well as in other plant organs (for reviews see James et al., 2003; Saripalli and Gupta, 2015). Various abiotic stresses including heat and drought inhibit the activity of AGPase leading to significant losses in crop yield (Boehlein et al., 2008; for a review see Saripalli and Gupta, 2015). Therefore, attempts have also been made in maize to engineer the AGPase to obtain its thermostable variants (Hannah et al., 2012; Boehlein et al., 2015). However, the precise nature of the metabolic and developmental changes that result due to genetic engineering of AGPase remains to be elucidated (Smidansky et al., 2002, 2003, 2007; Hannah et al., 2012).

AGPase occurs in both, cytosol and plastids. In cereals, a major part of ADP-glucose (ADP-G) is synthesized mainly in the cytosol of the developing endosperm cells, although some ADP-G is also synthesized in the plastids (Denyer et al., 1996). However, in dicotyledonous plants, AGPase-mediated ADP-G synthesis primarily occurs in the plastids (Beckles et al., 2001a,b). The plastidial AGPase also encodes transit peptides for transport of the newly-synthesized AGPase subunits into the plastids. Both plastidial and cytosolic AGPases function as heterotetramers (α2β2), each composed of two similar small subunits, SS (α2) and two similar large subunits, LS (β2) (Preiss et al., 1990). The SS and the LS differ only slightly in their molecular weights, and are encoded by different nuclear genes (for a review see Georgelis et al., 2007). The SS has both catalytic and regulatory functions, whereas the LS have largely a regulatory function (Kavakli et al., 2001). The relative roles of the two subunits suggest that the LS in the seed endosperm plays an important role in determining grain yield in cereals (Lee et al., 2007; Li et al., 2011; Hannah et al., 2012; Kang et al., 2013), while SS in the leaf contributes to plant biomass (Li et al., 2011; Schlossar et al., 2014). Two different types of AGPase SS genes (Type 1 and Type 2) have been reported in most of the cereals. Type 1 genes give rise to two transcripts; one is cytosolic endosperm and second is leaf plastidial. Similarly, Type 2 genes encode endosperm plastidial protein (Johnson et al., 2003; Rösti and Denyer, 2007; Kang et al., 2010). In case of rice and maize, AGPase LS is encoded by four different genes; one of these four genes is mainly expressed in embryo and remaining three genes are either expressed in embryo or leaf or both (Bae et al., 1990; Lee et al., 2007). However, in wheat (Johnson et al., 2003; Kang et al., 2010) and barley (Burton et al., 2002; Johnson et al., 2003), LS is known to be encoded by two genes which are mainly expressed in endosperm and embryo.

The genes encoding the LS were cloned from maize and hexaploid wheat (Shaw and Hannah, 1992; Thorneycroft et al., 2003; Rose et al., 2016); however, genes for the SS were cloned only from maize and potato (Nakata et al., 1994; Hannah et al., 2001). Recently, the primary and secondary structures of the AGPase subunits have been analyzed *in silico* in some selected monocots (maize, wheat, rice and barley) and dicots (potato and *Arabidopsis*) (Rani et al., 2013), where major emphasis was laid on the study of physico-chemical properties of amino acids and their importance in conferring stability to proteins. Using the crystal structure of the potato SS (Jin et al., 2005), *in silico* 3D structure of SS in several monocots and dicots as well as that of heterotetramer in potato and rice and heterodimer in wheat have also been reported (Tuncel et al., 2008; Danishuddin et al., 2011; Dawar et al., 2013; Sarma et al., 2014).

The monomer units of AGPase LS and SS, each consists of an N-terminal catalytic domain (ADP_Glucose_PP) and a C-terminal β-helix domain (Lbh_G1P_AT_C). The catalytic domain mainly consists of a largely parallel but mixed seven-stranded β sheet covered by α helices, which represent a fold reminiscent of the dinucleotide binding Rossmann fold. N-terminal catalytic domain makes strong hydrophobic interactions with the C-terminal β-helix domain via an α helix (Jin et al., 2005; Rani et al., 2013).

Although considerable information about AGPase and its genes is available, but detailed information about the diversity and variation in structure, function and evolution of the underlying genes among different plant species is missing. Therefore, the present study was carried out using well characterized maize AGPase genes as a reference to identify and characterize its “true” orthologs from 10 plant species including both cultivated and model species along with two progenitors of wheat (*Triticum urartu* and *Aegilops tauschii*). Results for the following aspects in 11 species (including maize) are presented in this communication: (*i*) identification of the “true” orthologs of maize LS and SS genes in 10 species; (*ii*) synteny and collineraity analyses across the 11 species; (*iii*) evolution of gene structure for both subunits among the monocots and the dicots; (*iv*) identification of motifs and domains of AGPase protein that are critical for proper functioning of the enzyme and the subtle differences between and within AGPases of monocots and dicots; (*v*) promoter and expression analysis, revealing novel features that might have bearing on the spatial and temporal expression of AGPase genes. Possible use of this knowledge for crop improvement is also discussed.

## Materials and methods

### Identification of “true” orthologs of genes for LS and SS

Full-length cDNA sequences of the maize *Sh2* (encoding LS) and the *Bt2* (encoding SS) were used as references in tBLASTx to identify “true” orthologs for the genes encoding LS and SS in seven monocots (*Triticum urartu, Aegilops tauschii*, wheat, rice, barley, sorghum and *Brachypodium*) and three dicot plant species (*Arabidopsis*, chickpea and potato). Criteria as described in Dhaliwal et al. (2014) were used to identify the “true” orthologs. Briefly, “true” orthologs were identified based on: (*i*) high level of sequence identity and query coverage along the protein length; (*ii*) presence of all domains and motifs present in the original query sequence; (*iii*) conservation of the relative size and sequence interval among motifs and domains of the query sequence with those of different species. Retrieved gene sequences were then used to identify the full length gene sequences from various sources including NCBI/EMBL/ Ensembl Plants (http://www.ncbi.nlm.nih.gov/)/ (http://www.embl.org/)/ (http://plants.ensembl.org/index.html).

### Gene structure analysis

Intron-exon junctions in the full length gene sequences were determined using the genomic and coding DNA sequences (CDS) for different species. The following three intron phases were marked depending on their position relative to the reading frame: phase 0 (intron insertion between two codons), phase 1 (insertion after the first base of a codon) or phase 2 (insertion after the second base of a codon). Ka/Ks values defining the magnitude of non-synonymous to synonymous substitutions were calculated using MEGA version 6.0.6 (dated April 2015) employing Jukes Cantor substitution model (Jukes and Cantor, 1969). The GC content was calculated both in exons and introns using the GC content calculator (http://www.endmemo.com/bio/gc.php). The gene sequences were also evaluated for the presence of simple sequence repeats (SSRs) and retro-elements using repeatmasker version 4.0.5 (http://www.repeatmasker.org/), using default parameters. About 1 kb genomic regions upstream of the translation start site (ATG) were evaluated for the presence of cis-regulatory response elements in the promoter regions, using PlantCARE database (Lescot et al., 2002). Only the response elements on the sense strand showing a matrix value of ≥5 were accepted, following the criteria described by Chen et al. (2007).

### Protein sequence analysis

Consensus amino acid (aa) sequence for LS and SS were generated through multiple sequence alignment of aa sequences belonging to all the orthologs through Geneious software ver 6.6.1 with default settings (http://www.geneious.com). An aa at a particular position present in majority of the orthologs was used for generating the consensus sequence. If a different aa was present at a particular position, aa in the maize reference was used for the consensus sequence. The insertions in any of the other species relative to the reference species were also added to the consensus sequence (Navarro et al., 2015). For sequence similarity, aa at different positions in a particular species were compared with the consensus sequence. In monocots, a similarity scale of 0–10 (a separate scale for each wheat homoeologue), and in dicots, a scale of 0–3 was used. A value of zero indicated complete lack of similarity with the consensus, while a value of 10 in monocots and 3 in dicots suggested conservation of aa in all of the species. Domains and motifs in the consensus protein sequence were identified through CDD analysis (http://www.ncbi.nlm.nih.gov/Structure/cdd/wrpsb.cgi).

### 3D structure analysis of the LS and the SS

The 3D structures of the LS and the SS were generated using aa sequences; for this purpose, Swiss-Model was used in an automated mode. The 3D structures for all the species thus generated were verified by both geometric and energetic means using the following servers: (*a*) Structure Analysis and Verification Server (SAVES) (http://nihserver.mbi.ucla.edu/SAVES) employing (*i*) PROCHECK to find out the relative proportion of aa, which fall in favored region, relative to other regions (Laskowski et al., 1993); (*ii*) VERIFY3D to determine the compatibility of an atomic model (3D) with its own aa sequence (Eisenberg et al., 1997) and (*iii*) ERRAT to analyse the statistics of non-bonded interactions between different atom types (Colovos and Yeates, 1993); (*b*) Swiss-Model server using structure assessment tool. FATCAT server (Ye and Godzik, 2004) was used to confirm the 3D structures of LS and SS by superimposing the 3D structures of LS/SS for each of the 10 plant species on the 3D structures of LS/SS of maize.

### MD simulation of AGPase structures

The molecular function of proteins depends on their conformational behavior and structural stability, which can be studied in real time environment through atomic-level perturbations using Molecular Dynamics (MD) simulations (Gupta et al., 2015a,b). In the present study, MD simulations were performed using Desmond v4.2 and system generation and results were generated using Schrodinger's maestro v11 platform (Schrödinger, Inc.).

Initially, the 3D structures of LS (chains A and C) and SS (chains B and D) of each species were used to generate the heterotetramer structures of AGPase using the potato AGPase (1YP2, Jin et al., 2005) as a template, which was followed by energy minimization of all the heteroterameric AGPase proteins structures. The heterotetrameric structure of each protein was embedded into an orthorhombic box with dimension of 10Å × 10Å × 10Å using TIP4P water solvent model. In order to ensure a complete solvent coverage over all tetrameric AGPase models, the box volume was recalculated and minimized to suit each model. Neutralization of each system was carried out by adding the required Na^+^ ions. Further, a full system minimization with solute was performed using hybrid of steepest descent and limited memory Broyden-Fletcher-Gold farb-Shanno LBFGS algorithms for 5000 consecutive iterations with a convergence threshold of 1.0 kcal/mol/Å (Liu and Nocedal, 1989). In the next phase, whole system of all models was subjected to NPT ensemble with 300 K temperature and 1.0325 bar pressure for 50 ps (picoseconds). Finally, MD production run was performed for 10 ns (nanoseconds) for all systems, using Nose-hoover thermostat at 300 K and Martyna-Tobias-Kien barostat method with relaxation of 1 ps and 2 ps, respectively. The calculation of potential and total energy, Root Mean Square Deviation (RMSD) and Root Mean Square Fluctuation (RMSF) of Cα atoms and backbones of structures in each trajectory were analyzed with respect to corresponding simulation time. The stability of all tetramer protein structures was determined on the basis of RMSD, average potential and total energy score. The RMSF measures the thermal motions of an individual residue and its fluctuation values over a well-defined average position. Finally, the B-factor of all tetramer structures reveal the fluctuation of atoms from their average positions and these render the crucial information about the protein backbone and side chains movements, which occurs due to thermal motion and the kinetic energy of individual atoms (Yuan et al., 2005). The energy minimized heterotetramer structures were then compared to the potato homotetramer (1YP2) through Schrodinger's maestro v11 platform (Schrödinger, Inc.).

### Ligand binding site analysis

Analysis for ligand binding sites was performed for only LS (using software 3DLigandSite; Wass et al., 2010), since information for SS was already available for wheat, rice, barley, *Arabidopsis* and chickpea. However, for the SS of the remaining six species, analysis could not be carried out, since no output was available using the software 3DLigandSite.

### Phylogenetic, synteny, and collinearity analysis

Phylogenetic analysis using the aa sequences of the two subunits was undertaken using MEGA version 6 (Tamura et al., 2011). Neighbor-joining method of distance matrix, with a bootstrap involving 1000 iterations, was used to construct an unrooted phylogenetic tree. Using blocks of genes associated with *Sh2* and *bt2* genes of maize, synteny and collinearity of the genes for the LS and the SS was studied using the online tool Genomicus (Muffato et al., 2010).

### Expression analysis

*In silico* expression analysis for the AGPase orthologs was carried out for maize, rice, wheat, barley and *Arabidopsis* using the “Genevestigator” microarray database. In case of wheat, *in silico* expression analysis was also carried out using transcriptome data from wheat expression database (http://wheat.pw.usda.gov/WheatExp/).

## Results

### Gene sequences and structure

#### “True” orthologs of genes encoding maize AGPase

The “true” orthologs of the genes encoding maize AGPase were identified from 10 different plant species using the criteria mentioned in the material and methods section. The genomic, cDNA and CDS of the LS and the SS genes from the 11 plant species (including maize) thus obtained are presented in Tables 1, 2. In all the species examined, a single ortholog each for LS and SS was found except in case of wheat where three orthologs each for LS and SS were found, one each in the three genomes (A, B, and D genomes). The similarity of cDNA sequences of LS for 10 species with that of maize LS (used as a reference sequence) ranged from 64.1 to 92.5% in monocots and from 56.2 to 59.2% in dicots; similarly, the similarity of SS ranged from 71.5 to 86.8% in monocots and from 67.9 to 69.1% in dicots. Similarity of CDS sequences of LS ranged from 70.6 to 94.8% in monocots and from 61.7 to 63.5% in dicots; similarly, the similarity of SS ranged from 79.8 to 86.1% in monocots and from 72.8 to 73.6% in dicots. However, the similarity level of gene sequences relative to cDNA and CDS sequences was low (for LS, it ranged from 36.9 to 78.8% in monocots and from 39.4 to 40.6% in dicots; for SS, it ranged from 38.3 to 44.4% in monocots and 33.1 to 34.9% in dicots).

**Table 1:** **Details of cDNAs, CDS, proteins and gene sequences for AGPase LS in different monocots and dicots with respect to genes for maize AGPase LS**.

**Species LS**	^*^cDNA		**CDS**		**Protein**		**Gene**	
	**Length (bp)**	**% similarity**	**Length (bp)**	**% similarity**	**Length (aa)**	**% similarity**	**Length in bp (chromosome assignment)**	**% similarity**
Maize	1911	–	1551	–	516	–	7320 (3L)	–
Wheat^**^	1855	66.0	1569	71.6	522	70.7	4773 (1AL)	40.7
	1947	63.8	1569	70.6	522	70.5	7725 (1BL)	39.0
	2646	64.0	1569	70.6	522	70.5	4432 (1DL)	40.9
*T. urartu*	1377	69.6	1377	73.9	458	73.4	4635 (1AL)	38.9
*Ae. tauschii*	1725	66.5	1725	70.6	574	70.5	3474 (1DL)	41.2
*Brachypodium*	2097	65.1	1569	73.2	522	70.4	5078 (2)	41.5
Rice	1906	75.3	1557	82.0	518	76.9	8079 (1)	43.3
Barley	1786	66.5	1572	71.0	523	70.6	4367 (1)	36.9
Sorghum	1921	92.5	1554	94.8	517	91.2	4774 (3)	78.8
*Arabidopsis*	2049	58.7	1557	63.5	518	56.9	3313 (1)	39.4
Chickpea	2233	57.3	1557	63.5	518	57.4	4249 (5)	40.6
Potato	2166	56.5	1584	61.7	527	56.7	4985 (UI)	40.7

* Accession id's for maize, NM_001127632; wheat 1AL, Traes_A1B2A8EB0.1; wheat 1BL, DQ839506; wheat 1DL, Traes_844FE40E6.1; T. urartu, TRIUR3_17989-T1; Ae. tauschii, F775_52189, EMT32758; Brachypodium, XM003567769; rice, EU267956; barley, KF442975; sorghum, XM002455967; Arabidopsis, NM102533; chickpea, XM004500747; potato, XM006365058;

** *indicates wheat homoeologues of group 1 chromosomes*.

**Table 2:** **Details of cDNAs, CDS, proteins and gene sequences for AGPase SS in different monocots and dicots with respect to maize AGPase SS**.

**Species**	^*^cDNA		**CDS**		**Protein**		**Gene**	
	**Length (bp)**	**% similarity**	**Length (bp)**	**% similarity**	**Length (aa)**	**% similarity**	**Length in bp (chromosome assignment)**	**% similarity**
Maize	1754	–	1428	–	475	–	6076 (4)	–
Wheat^**^	1853	78.3	1422	85.6	473	90.8	7962 (7AS)	42.9
	3679	76.6	1545	83.7	514	88.2	7605 (7BS)	39.9
	1508	83.2	1422	85.7	473	89.0	6719 (7DS)	41.1
*T. urartu*	1761	75.3	1761	80.3	586	82.8	6514 (7AS)	39.5
*Ae. tauschii*	1422	86.8	1422	86.0	473	89.5	6067 (7DS)	41.6
*Brachypodium*	1686	80.8	1425	86.1	474	90.0	5277 (3)	38.9
Rice	1833	78.6	1545	83.7	514	89.0	6248 (8)	40.5
Barley	1820	79.0	1419	85.3	471	89.4	9669 (UI)	38.3
Sorghum	2122	71.5	1533	80.0	510	83.3	4902 (2)	44.4
*Arabidopsis*	1896	67.9	1563	72.8	520	84.6	2565 (5)	34.6
Chickpea	1861	69.1	1560	73.6	519	83.6	3012 (2)	34.9
Potato	1833	69.7	1566	72.8	521	84.2	5820 (7)	33.1

* Accession id's for maize, NM_001111568; wheat 7AS, TRIAETRIAE_CS42_7AS_TGACv1_569682_AA1821750; wheat 7BS, TRIAETRIAE_CS42_7BS_TGACv1_591785_AA1921060; wheat 7DS, AF492644; T. urartu, TRIUR3_26024-T1; Ae. tauschii, F775_30798; Brachypodium, XM003573742; rice, FJ940194; barley, Z48562; sorghum, XM_002462095; Arabidopsis, NM_124205; chickpea, XM004491501; potato, DQ207843;

** *indicates wheat homoeologues of group 7 chromosomes*.

#### Chromosome assignment for LS and SS genes

The chromosomes and the respective arms on which LS and SS genes are located are provided in Tables 1, 2. LS genes were located on the long arms of wheat homoeologous group 1 (1AL, 1BL and 1DL) and the corresponding chromosome arms of other species. Similarly, SS genes were located on the short arms of wheat homoeologous group 7 (7AS, 7BS, and 7DS) and the corresponding chromosome arms of other species.

#### Gene structure comparison

The gene structures for different species were compared, using the reference genes in maize. Major differences were observed in the sequence lengths of the genes (3.3–7.7 kb in LS and 2.5–9.6 kb in SS). The gene for LS was longest in rice and smallest in *Arabidopsis* (Table 1). Similarly, the gene for SS was longest in barley and smallest in *Arabidopsis* (Table 2). This variation in gene length is mainly attributed to the difference in the number and size of the introns, as was apparent from comparison of cDNA sequences, which lack introns (1.377–2.646 kb in case of LS and 1.508–2.122 kb in case of SS). The cDNAs for genes for both LS and SS were longer in dicots than in monocots. However, in case of LS, the situation was just the opposite in case of *Brachypodium* and the gene on 1DL of wheat (Table 1). Similarly, for SS the situation was opposite in case of sorghum and the gene on 7BS of wheat (Table 2). Only marginal differences were observed in CDS except in case of LS for *T. urartu*, where the CDS was shortest and *Ae. tauschii*, where the CDS was longest. Thus the observed length variation in cDNA is due to variation in the lengths of UTRs in the cDNAs.

**Figure 1:**
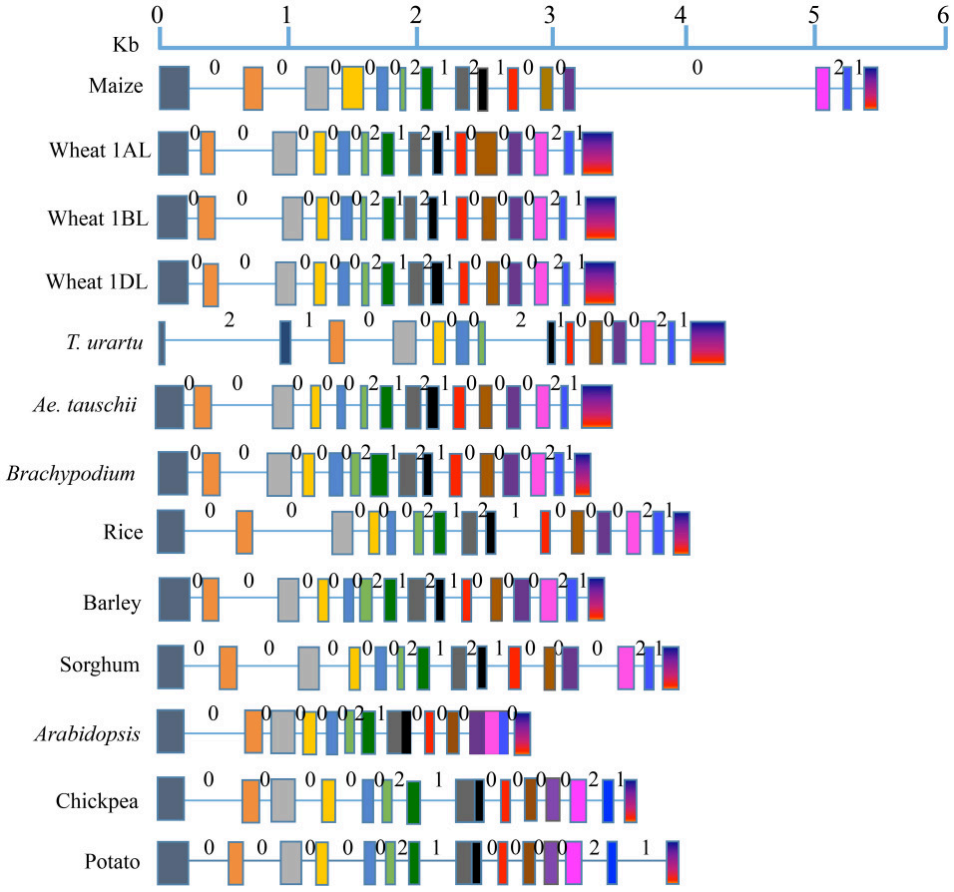
**Gene structure for AGPase LS from translation start to stop sites in eight monocots (including all the wheat homoeologues) and three dicots**. Solid boxes indicate exons and lines indicate introns. Exons are color coded based on the sequence similarity with the respective exons in the AGPase LS gene of maize (used as reference). Intron phases 0, 1, and 2 are marked above each intron.

**Figure 2:**
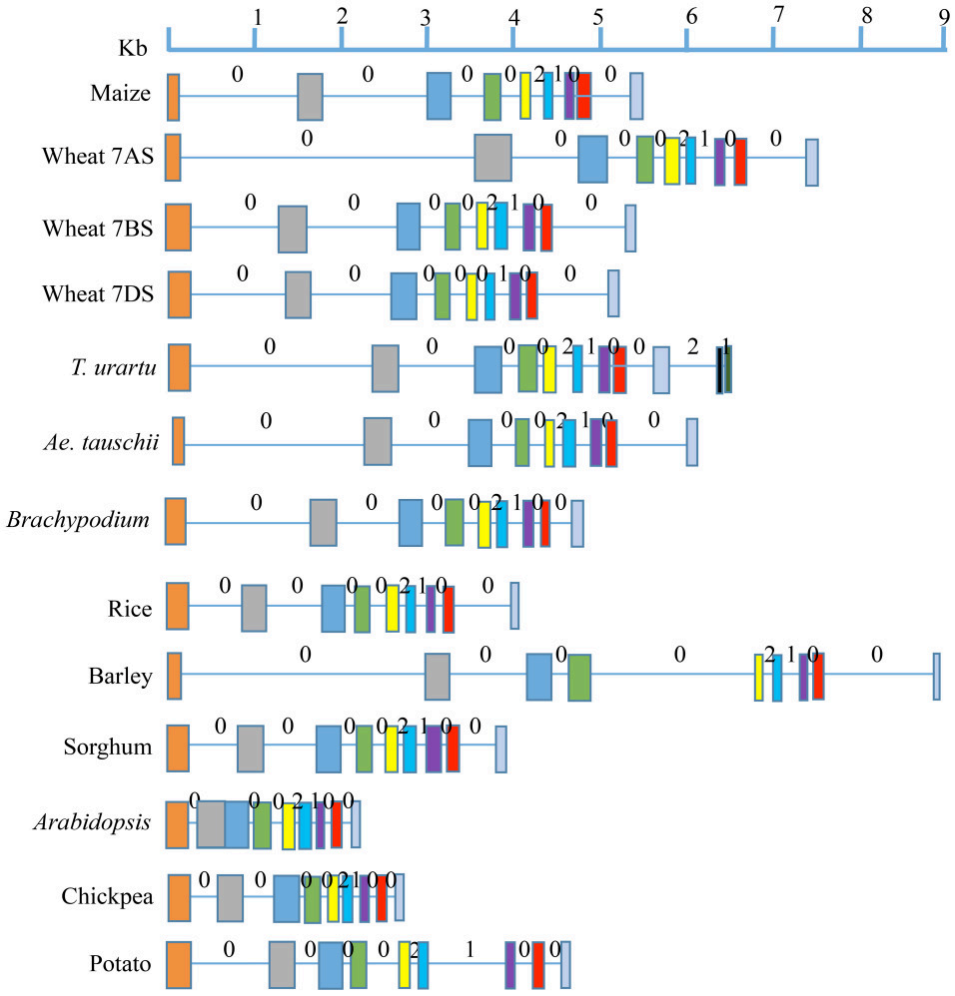
**Gene structure for AGPase SS from translation start to stop sites in eight monocots (including all the wheat homoeologues) and three dicots**. Solid boxes indicate exons and lines indicate introns. Exons are color coded based on the sequence similarity with the respective exons in the AGPase SS gene of maize (used as reference). Intron phases 0, 1, and 2 are marked above each intron.

The number of exons and introns in the LS and SS genes showed some variation among the 11 plant species (Figures 1, 2; Supplementary Tables 1, 3). The LS genes each had 15 exons in monocots (except in *T. urartu*, which had 14), and 14 exons in dicots (except in *Arabidopsis* which had 12 exons). The SS genes, each had 9 exons both in monocots and dicots except for *T. urartu* with 11 exons and *Arabidopsis* with 8 exons.

Variation was also observed in the size of introns and exons. Introns in LS genes were generally shorter than those in SS genes, which also showed a wider range of variation in intron size (34–3532 bp) relative to LS genes (66–605 bp); the third last intron in LS gene of maize was an exception (1821 bp). The variation in lengths of exons for LS (range: 56–274 bp) and SS (range: 74–298 bp) was relatively low, second exon of *Arabidopsis* SS gene being the only exception (569 bp).

All the three types of intron phases (0, 1, and 2) were found in genes for LS and SS both in case of monocots and dicots (Figures 1, 2). Among all species examined, and for both subunits, phase 0 was the most prevalent (for LS, frequency of phase 0 ranged from 53.8 to 57.1% in monocots, and 69.2 to 81.8% in dicots; for SS, it ranged from 60.0 to 87.5% in monocots and from 71.4 to 75.0% in dicots). This was followed by equally frequent phase 1 and phase 2, except for SS in monocots (for LS, phases 1 and 2 ranged from 21.4 to 23.1% in monocots and 9.1 to 15.4% in dicots; for SS, from 12.5 to 14.3% in dicots). For SS in monocots, phase 1 was more frequent (12.5 to 20.0%) relative to phase 2 (0 to 12.5%).

**Figure 3:**
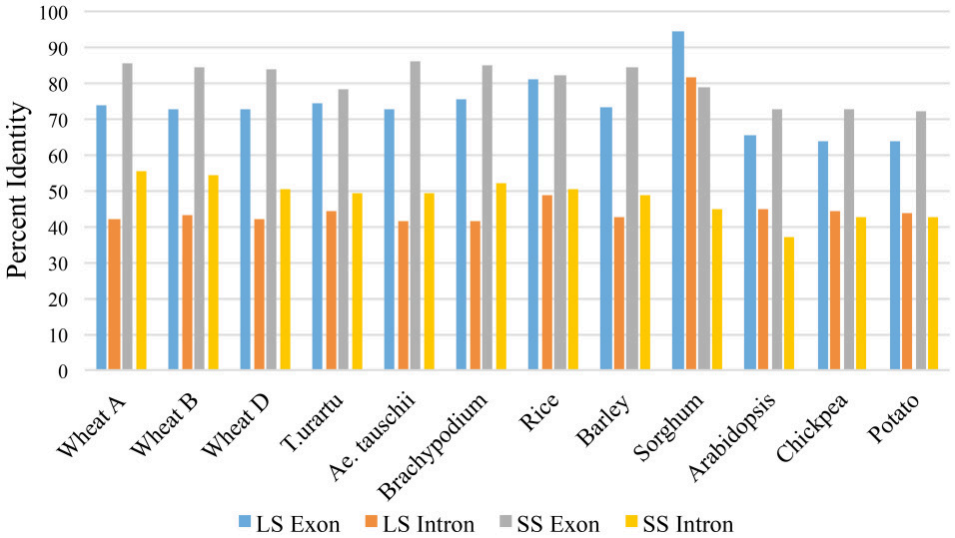
**Percent similarity of exons and introns in LS and SS of monocots and dicots with respect to exons and introns of maize LS and SS AGPase genes**.

For LS genes, the average sequence similarity for exons was higher in monocots (72.6 to 94.4%) than in dicots (64.0 to 65.2%), whereas the average sequence similarity for introns was low in both monocots (41.4 to 48.9%) and dicots (43.4 to 44.9%) except for introns in sorghum, where the similarity was 81.6% (Supplementary Table 2; Figure 3). For SS genes, however, exons and introns, both exhibited marginally higher sequence similarity in monocots (78.1 to 85.8% for exons; 44.7 to 56.0% for introns) than in dicots (72.3 to 73.0% for exons; 37.0 to 42.8% for introns) (Supplementary Table 4).

The Ka/Ks values for the LS genes in monocots were higher (2.92) than in the dicots (0.697) (Supplementary Tables 5, 6). In contrast, the Ka/Ks values for the SS genes were marginally higher in the dicots (5.54) than in the monocots (5.26) (Supplementary Tables 7, 8).

The LS and the SS genes had variable GC content in exons and introns, but most GC-rich exons were flanked by GC-poor introns. The GC rich exons ranged from 62.28 to 92.30% in LS and 62.5 to 100% in SS, although in *Arabidopsis* SS, the GC-poor exons were flanked by GC-rich introns (detailed data not presented). The GC content was generally higher for the SS genes than for the LS genes in most of the plant species examined except in *T. urartu, Ae. tauschi, Brachypodium* and wheat (homoeologous group 1). For LS genes, the length and GC content of exons were positively correlated (*r* = 0.10 to 0.57), while those for introns were negatively correlated (*r* = −0.09 to −0.34) with a few exceptions. For SS genes, the length and GC content of both introns and exons were positively correlated (*r* = 0.15 to 0.78 for introns; *r* = 0.02 to 0.81 for exons) except wheat orthologs on 7AS and 7BS, where the results were the opposite.

#### Synteny/collinearity analysis

Analysis of synteny conservation was undertaken using a block of 31 genes, including 15 genes flanking either side of the AGPase LS gene on maize chromosome 3 and SS gene on chromosome 4. This analysis was possible for only nine of the eleven species, since chickpea genome sequences were not available in Ensemble Plants, and those of wheat genome could not be utilized by Genomicus. For SS genes, no synteny conservation was observed (Supplementary Figure 1), but for LS genes, some degree of synteny conservation was observed among maize, sorghum and rice (Supplementary Figure 2). Synteny of 19 of the 30 genes flanking the maize LS gene was shared on the corresponding chromosome 3 of sorghum, and for 12 of the 30 genes in case of rice chromosome 1. Even in these two species (rice and sorghum), the collinearity within the synteny block was rather disrupted. Microsynteny, in terms of nucleotide sequences of the AGPase LS and SS genes has already been described earlier, while presenting the results of gene structure.

#### SSRs and retro-elements in the LS and SS genes

SSRs were available in both, the LS and SS genes belonging to some of the species (Supplementary Tables 9, 10). Retro-transposons (LINEs and LTR elements), SINEs and transposons were also identified in some of these genes which were present in introns except for rice SS gene; in *Brachypodium* SS gene, no retero-element/transposon was found (Supplementary Table 10).

#### Promoter analysis of the LS and the SS genes

**Figure 4:**
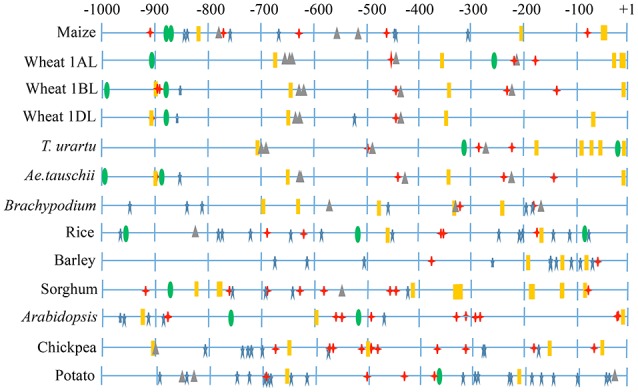
**Representative figure showing regulatory elements identified in 1 kb upstream region of AGPase LS**. Different symbols indicate major regulatory elements identified. TATA box (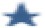), CAAT box (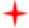), light responsive response elements (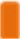), abiotic stresses responsive elements (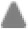), endosperm expression responsive elements (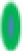).

Promoter analysis allowed identification of cis-regulatory elements in the 1 kb upstream regions of both the LS and the SS genes. These elements (e.g., MBS, GARE, ABRE) presumably respond to abiotic stresses and also to hormones like gibberellic acid and absicic acid. Regulatory elements responsible for tissue-specific expression (e.g., endosperm expression) and those with unknown functions were also identified (Figure 4 and Supplementary Figure 3). In both, LS and SS genes, regulatory elements including TATA box were frequent, with the following exceptions: (i) LS gene of wheat (1BL) and *Ae. tauschii*, and (ii) SS gene of wheat (7DS), *Brachypodium* and *Arabidopsis*. Response elements were absent in LS gene of *T. urartu* and wheat 1AL.

### Protein sequences and structure

#### Comparison of predicted protein sequences

The range of variation in the length of protein sequences of LS and SS in monocots (458 to 574 aa in LS and 472 to 586 aa in SS) was higher than in the dicots (518 to 527 aa in LS and 519 to 521 aa in SS) (Tables 1, 2). A comparison of the aa sequences of the LS and the SS genes with respect to that of maize showed a higher similarity for SS (82.8 to 90.8% for monocots and 83.6 to 84.6% for dicots) than for LS (66.9 to 89.0% for monocots and 54.0 to 54.4% for dicots; for more details about the variation in the protein sequences, see below).

##### Protein sequences in LS

**Figure 5:**
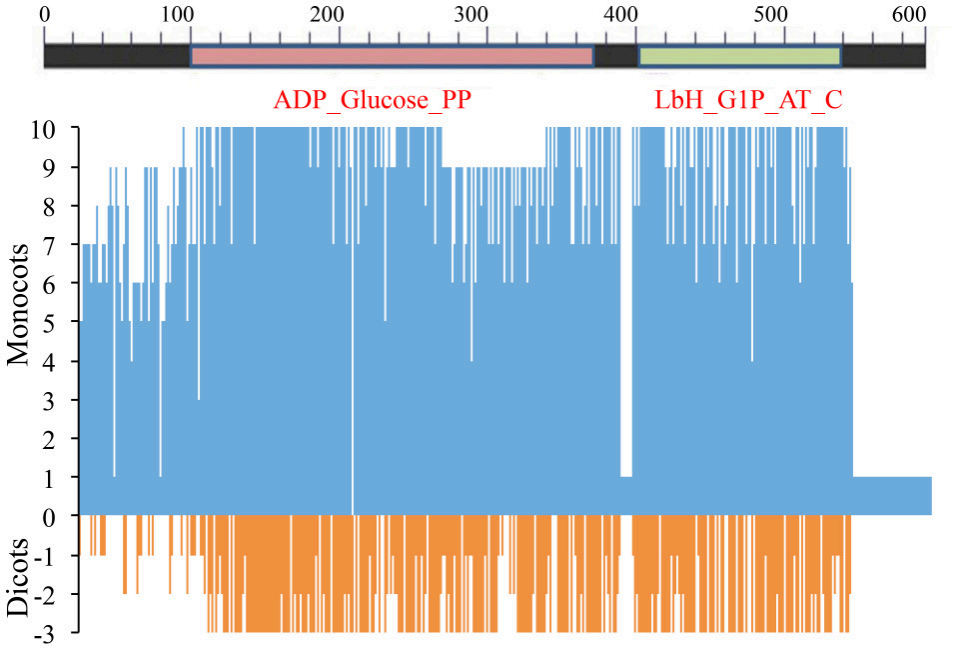
**Amino acid sequence similarity of AGPase LS among 8 monocots (including wheat homoeologues on group 1 chromosomes) and 3 dicots with respect to consensus sequence**. Position 0 (on y-axis) indicates amino acid consensus sequence. Presence of similar amino acids against consensus is plotted on a scale of 1–10 in monocots (blue) and 1–3 in dicots (red).

Among all species examined the predicted LS protein in *Ae. tauschii* was longer than that of the other species. The aa sequence was variable, and on an average only 29% residues were conserved. The LS had two conserved domains (CDs), namely ADP_Glucose_PP (aa 98–374) and Lbh_G1P_AT_C (aa 407–533). The N-terminal region (aa 1–97) was hyper-variable, but the linker region (aa 375–406) connecting the two domains was largely conserved. Variation was mainly due to deletions, insertions and mismatches (Figure 5 and Supplementary Figure 4).

In monocots, with respect to the consensus sequence, variations observed were classified in two categories (aa absent and additional aa present):

**(a) Amino acids (aa) absent**: (*i*) proline at position 11 and leucine at position 13 in sorghum; (*ii*) arginine at position 58 and glycine at position 59 in rice and sorghum; (*iii*) arginine at position 58 in *Brachypodium*; (*iv*) 69 (sixty nine) aa residues at positions 257–325 in *T. urartu*; and (*v*) alanine at position 76 in each of the following four species: maize, rice, sorghum and *Brachypodium*.
**(b) Additional amino acids (aa) present**: (*i*) cysteine at position 26 in maize; (*ii*) alanine and glycine in rice, serine and cysteine in sorghum and asparagine and valine in *Brachypodium* at positions 25 and 26, respectively; (*iii*) methionine at position 35 in rice; (*iv*) methionine in *T. urartu* and alanine in barley at position 57; (*v*) glutamine at position 84 in maize and rice; and (*vi*) a block of nine aa at positions 378–386 in *Ae. tauschii*.

In dicots, the above two classes of sequence variations included the following:

**(a) Amino acids (aa) absent**: (*i*) glutamic acid at position 27 and alanine, glutamine and cysteine at positions 65–67 in *Arabidopsis*; (*ii*) serine at position 40 in potato; and (*iii*) arginine and glycine at positions 58 and 59, respectively, in chickpea.
**(b) Additional amino acids (aa) present**: (*i*) proline at position 84, lysine at position 191 and tyrosine at position 212 in all the dicots; (*ii*) alanine at position 12, serine at position 35, proline at position 57 and asparagine at position 165 in *Arabidopsis*; (*iii*) cysteine at position 12, lysine at each of three positions (25, 26, and 35) and serine at position 381 in chickpea; and (*iv*) valine at position 12, glycine at position 25, glutamic acid at position 26, arginine at position 35, lysine at position 57 and phenylalanine at position 381 in potato.

##### Sequence variation in SS

**Figure 6:**
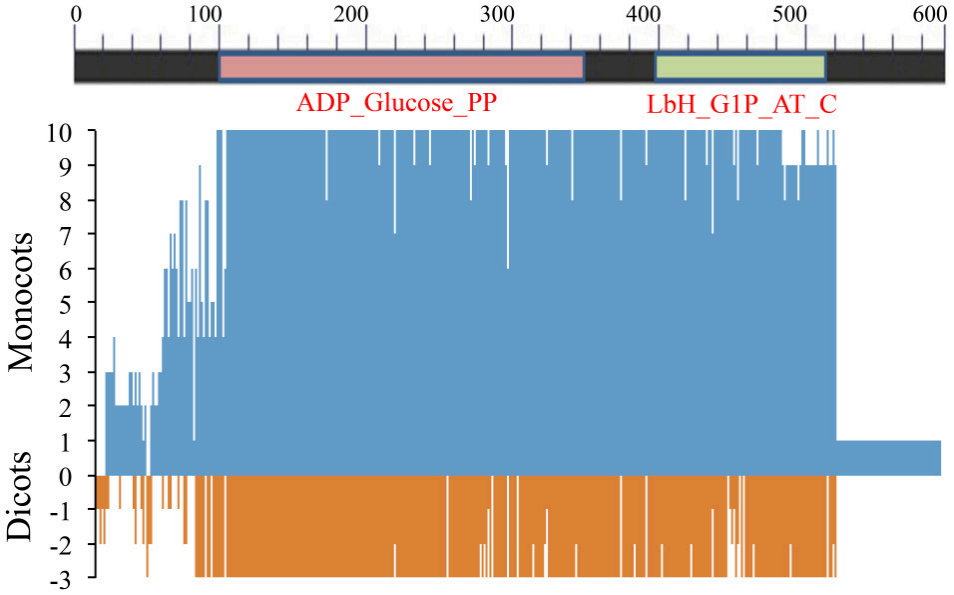
**Amino acid sequence similarity of AGPase SS among 8 monocots (including wheat homoeologues on group 7 chromosomes) and 3 dicots with respect to consensus sequence**. Position 0 (on y-axis) indicates amino acid consensus sequence. Presence of similar amino acids against consensus is plotted on a scale of 1–10 in monocots (blue) and 1–3 in dicots (red).

The predicted SS protein was relatively longer in *T. urartu* than the one in any of the other species. Unlike the low level of conservation for LS, ~55% of aa residues in SS were conserved for each of the 11 species examined. However, like LS, SS also had a hyper-variable N-terminal region (aa 1–95) associated with ADP_Glucose_PP domain (aa 96–354), Lbh_G1P_AT_C domain (aa 392–517) and a connector region (aa 355–391). The SS also had sequence variations, some of which were species-specific. Following sequence variations were observed in SS:

**(a) Amino acids (aa) absent**: (i) phenylalanine at positions 59 and lysine at position 170 in barley; (ii) arginine at position 63 in wheat orthologs on 7AS and 7DS, *Ae. tauschii* and barley; (iii) cysteine at position 72 in *Brachypodium*; and (iv) isoleucine at position 509 and valine at position 510 in *T. urartu*.
**(b) Additional amino acids (aa) present**: (i) at position 70, glutamic acid in maize, lysine in *Brachypodium* and threonine in chickpea; and (ii) at positions 82 and 83, respectively, aspartate and serine in maize, lysine and histidine in wheat orthologs (7AS, 7DS), *Ae. tauschii* and barley and lysine and proline in *Brachypodium* (Figure 6 and Supplementary Figure 5).

“QTCL” motif in SS that was earlier reported in potato (Linebarger et al., 2005) was also identified in the remaining two dicots (*Arabidopsis* and chickpea) and few monocots including *T. urartu*, wheat (7BS), rice and sorghum.

#### 3D structure comparison of AGPase LS and SS

The 3D structures that were generated for each of the 11 species using potato SS homotetramer as a template (PDB id: 1YP2) had a high level of confidence, as inferred from the following observations (Supplementary Table 11): (*i*) a high proportion of aa residues were in the favored region of Ramachandran plots (relative to “allowed” and “disallowed” regions); (*ii*) an overall value of the G-factor was within the acceptable range for all comparisons; (*iii*) the values of quality factors estimated by ERRAT and those of 3D-1D score estimated by VERIFY3D were high; (*iv*) graphical data [obtained from Anolea (Melo and Feytmans, 1998), Qmean (Benkert et al., 2008), and GROMOS (van Gunsteren and Billeter, 1996) (data not presented)] based on Swiss-Model showed negative energy values, suggesting a favorable energy environment for given amino acids, which was also reflected by the acceptable values of *Dfire energy, QMEAN6* and *GMQE* (Supplementary Table 12).

The predicted values of different parameters obtained through superimposition of the 3D protein structure for each species are presented in Supplementary Table 13. Pair-wise alignments of the 3D structures of LS and SS of each of the 10 species with the corresponding structures in maize showed a higher level of similarity, which ranged from 63.3 to 77.8% for LS in monocots, 62.4 to 64.7% for LS in dicots, 88.0 to 100% for SS in monocots and 90.3 to 91.2% for SS in dicots. Further, the 3D structures of LS and SS of each of the 10 species with the corresponding structures in maize showed a high level of similarity for physico-chemical properties ranging from 74.0 to 90.8% for LS in monocots, 78.2 to 80.3% for LS in dicots, 95.7 to 100% for SS in monocots and 95.7 to 96.2% for SS in dicots. The values for RMSD were 1.07 to 2.25 Å for LS in monocots, 0.81 to 1.57 Å for LS in dicots, 0.19 to 1.80 Å for SS in monocots and 0.33 to 0.85 Å for SS in dicots.

#### MD simulation analysis and validation of structures

**Figure 7:**
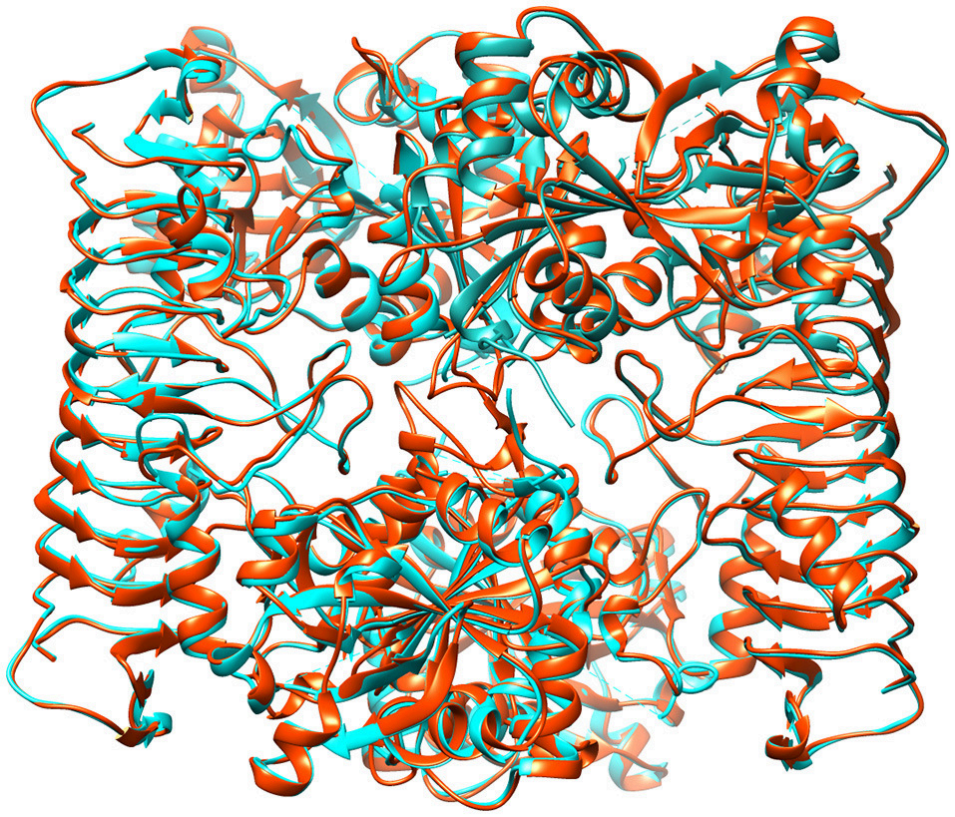
**Representative figure showing superimposed structure of the predicted wheat AGPase heterotetramer (orange colored) over potato AGPase homotetramer (cyan colored)**.

Initially, energy minimization in NPT ensemble for 50 ps was carried out for all modeled structures. Every chain of tetrameric structure was restrained during the production run of MD simulation for 10 ns, to reveal their stability in real time environment. The brief details such as number of atoms, average volume and added Na^+^ for each protein system is summarized in Table 3. The comparative RMSD plot for Cα atoms and backbone of protein showed that backbone deviation is higher than Cα carbon for all 11 heterotetramer structures (Supplementary Figure 6). This is due to their distance-dependent calculation method and it's usage to infer the stability (Rotkiewicz and Skolnick, 2008). Insight analysis of backbone RMSD showed that the average deviation of heterotetramer structures ranged from 0.519 to 0.541 Å in different species examined. This showed that all structures have marginal deviation differences, which indicated an overall good stability. In addition, the B-factor profiles and RMSF of Cα carbon and backbone of all structures showed the thermal motions of an individual residue and its fluctuation values over a defined threshold (Supplementary Figure 7A). Insight analysis of RMSF plots indicated that the backbone residues of *T. urartu, Ae. tauschii, Brachypodium*, rice and chickpea have higher fluctuations in comparison to others (Supplementary Figure 7B). Further, the protein structure validation statistics for all the heterotetramer structures indicated the acceptable ranges of all parameters (Table 3). Superimposition of all 11 heterotetrameric structures (Figure 7 and Supplementary Figures 8–17) over potato AGPase homotetramer (1YP2) showed RMSD values in the range of 0.250 Å in rice to 0.790 Å in *T. urartu*, indicating only minor structural changes, which suggested conservation of the predicted heterotetramer structure of the different species. Overall, the MD simulation analysis showed that all structures are stable and they can be further used in various structural analyses as AGPase proteins in dicots and monocots.

**Table 3:** **Various average energy parameters of each system after Molecular Dynamics (MD) simulation analysis**.

**Various energies and structural validation statistics**	**Maize**	**Wheat**	***T. urartu***	***Ae. tauschii***	***Brachypodium***	**Rice**	**Barley**	**Sorghum**	***Arabidopsis***	**Chickpea**	**Potato**
Total energy in Kcal/mol	−339,510	−345,632	−320019.9	−345768.8	−338027.48	−375,651	−373,275	−337,195	−344537.89	−353074.87	−342,517
Potential energy in Kcal/mol	−430,721	−437,789	−404186.1	−438186.7	−426788.93	−475,387	−472,755	−427257.56	−437024.80	−447208.06	−433,711
Volume (Å^3^)	1,547,957	1,558,700	1450820.1	1,561,993	1531665.44	1,439,503	1,429,764	1531994.11	1568638.944	1598327.28	1,556,526
Atoms in each orthorhombic box	182078	183,110	169,772	183,562	178,846	145,566	144,623	179,298	184,546	188,194	182,880
Added Na^+^ ions	29	44	42	43	45	50	46	45	22	32	20
dFire energy	−2647.3	−2461.2	−2253.5	−2659.85	−2511.89	−266.53	−2651.3	−2636.77	−2634.55	−2651.31	−2657.8
QMEAN6	0.698	0.691	0.66	0.68	0.681	0.704	0.692	0.695	0.69	0.692	0.701
Core region	84.2	83.6	82.8	83	82.6	83.3	84	84	82.9	82.7	83.9
Allowed region	14.7	14.9	15.7	15.4	16	15.5	14.8	14.3	16	15.2	14.9
Generously allowed region	0.9	1.3	1	1.1	1.1	0.9	0.9	1.2	0.5	1.6	0.8
Disallowed region	0.3	0.3	0.5	0.5	0.3	0.3	0.3	0.5	0.6	0.5	0.4
G factor	−0.97	−0.98	−0.99	−0.99	−1.01	−0.93	−0.94	−1	−0.97	−1.01	−0.97

### Ligand binding site analysis

**Figure 8:**
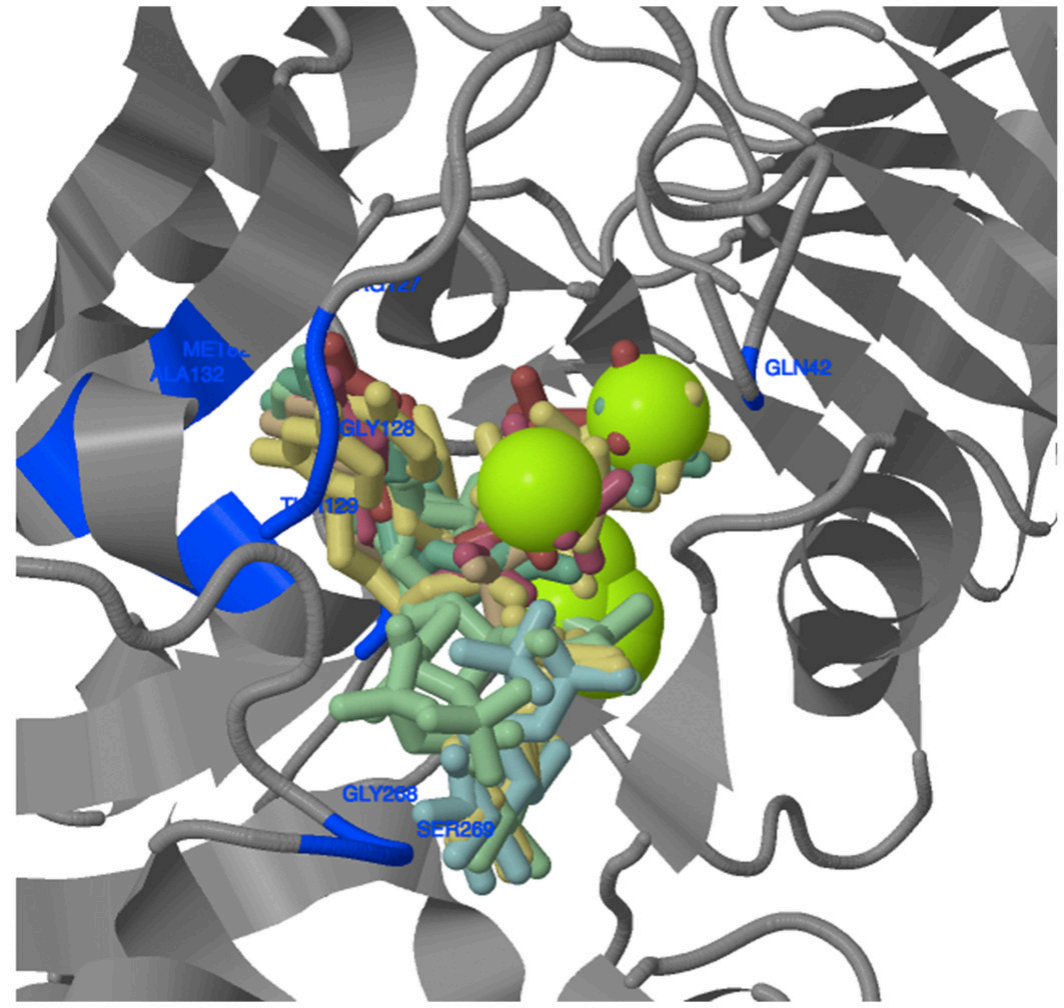
**3D structure of wheat AGPase LS protein**. The amino acids and their positions in the protein involved in ligand binding are shown in blue. Green sphere represent metallic heterogen (Mg^++^) involved in ligand binding.

The amino acids (aa) constituting the ligand binding sites were identified only in LS (Figure 8, Supplementary Table 14). Similar information for SS was already available for five of the 11 species; for the remaining six species (*T. urartu, Ae. tauschii*, maize, sorghum, potato, and *Brachypodium*), no output for ligand binding aa residues for SS was available following data analysis. Most of the ligand binding sites were confined to the domain ADP_Glucose_PP. Generally, 1–4 clusters of ligands were predicted for the binding of ATP, ADP and Mg^++^ (involved in AGPase activity). Amino acid residues ranging from 13 to 17 in number were involved in ligand binding in most monocots except for *T. urartu*, where only 4 aa residues were involved. Among dicots, ligand binding involved 13 aa in chickpea, and 14 aa in *Arabidopsis* and potato. In wheat, barley and *Brachypodium*, ~70% of the ligand binding sites were common, although their positions varied. A “LGGG” motif is also present in the LS gene of each species except *T. urartu* and chickpea. However, this motif was present in the N-terminal region of LS in all wheat homoeologues and *Ae. tauschii*; in the remaining species it was present in the ADP_Glucose_PP domain.

### Phylogenetic analysis

**Figure 9:**
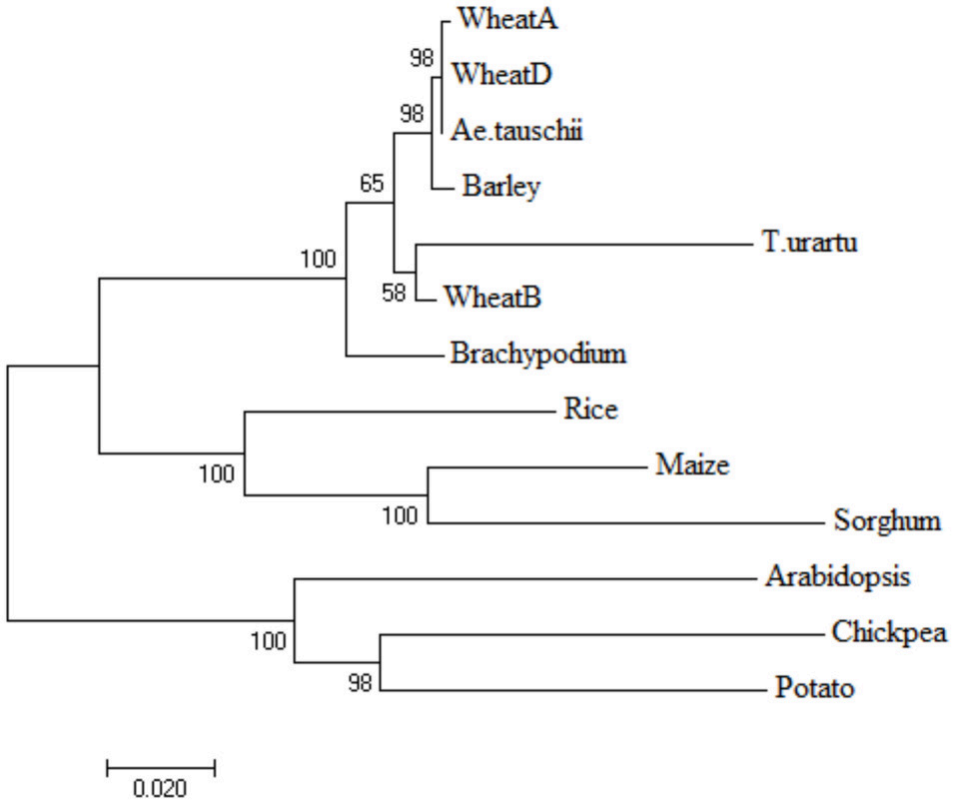
**Phylogenetic tree obtained by neighbor-joining method using amino acid sequences of proteins encoded by genes for AGPase LS+SS to depict the relationship among monocots and dicots**. The branch length represents magnitude of genetic change.

Based on the aa sequences, three separate phylogenetic trees were initially constructed, each using LS, SS, and LS + SS. Since the three trees were largely similar, only the tree based on aa sequences of the entire AGPase enzyme complex (LS + SS) is reported (Figure 9). The tree had two major clusters, one with the eight monocots, and the other with the three dicots. Among the monocots, wheat, *T. urartu, Ae. tauschii*, barley and *Brachypodium* were grouped into the sub-cluster I, while maize, sorghum and rice were grouped in a separate sub-cluster. There were two sub-clusters in cluster II, with one containing potato and chickpea and the other containing *Arabidopsis*.

### Expression analysis of AGPase genes

**Figure 10:**
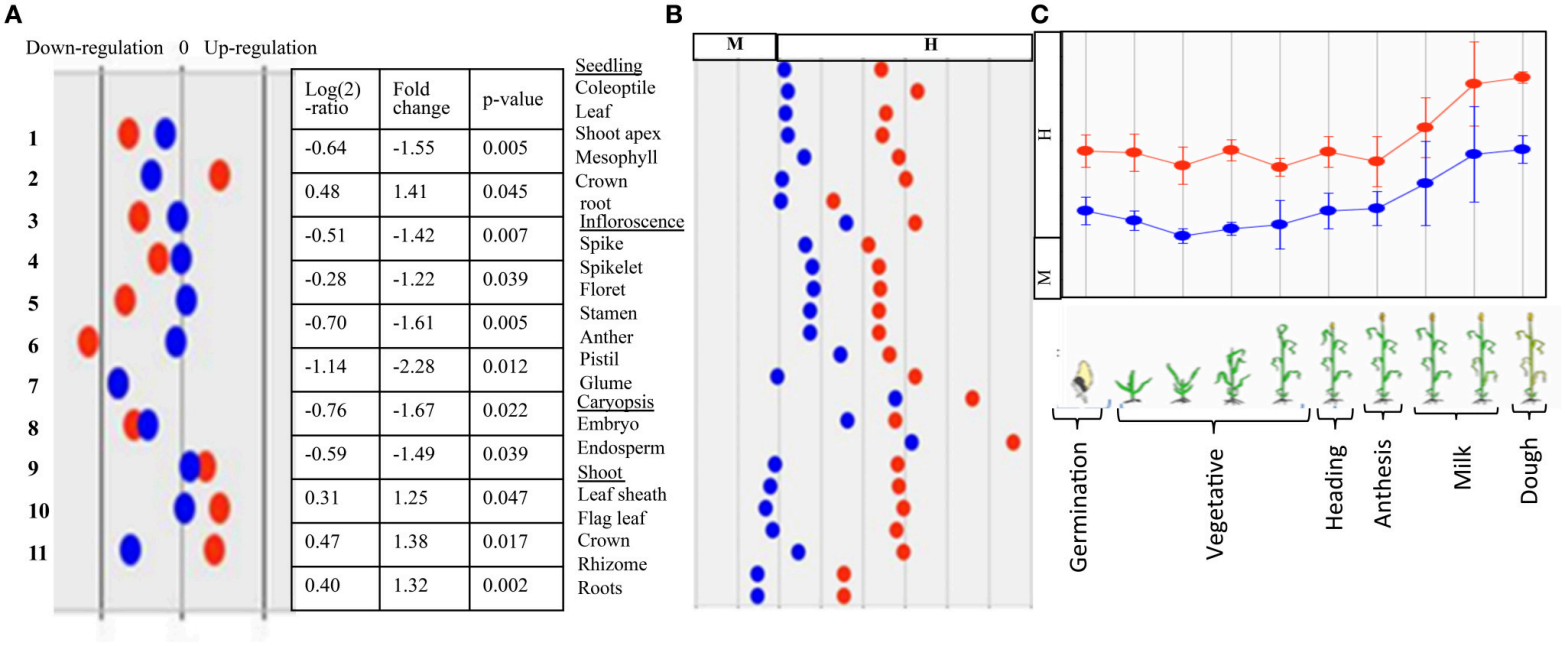
**Representative figure showing *in silico* expression of wheat AGPase LS and SS where 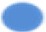 = transcript (probset id: Ta.2797.2.S1_x_at) encoding AGPase LS and 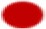 = transcript (probset id: Ta.242.1.S1_at) encoding AGPase SS. (A)** Expression during major abiotic stresses (drought and heat) where fold change is significant at *p* ≥ 0.05; Numbers on Y-axis indicate 11 different microarray experiments; **(B)** Expression in different tissues and **(C)** Expression at different plant developmental stages.

Expression of AGPase LS and SS genes was examined using the microarray and transcriptome data, as mentioned in the section dealing with material and methods. The expression analysis based on microarray data indicated that the level of expression of genes for both subunits (LS and SS) was highest in spike/endosperm tissues and at anthesis, which declined during dough stage in all of the four cereals examined (maize, wheat, barley and rice) (Figure 10); the expression was relatively low in vegetative tissues. In *Arabidopsis*, maximum expression was observed in leaves during bolting stage, which declined during senescence (no data was available for anthesis and grain development).

**Figure 11:**
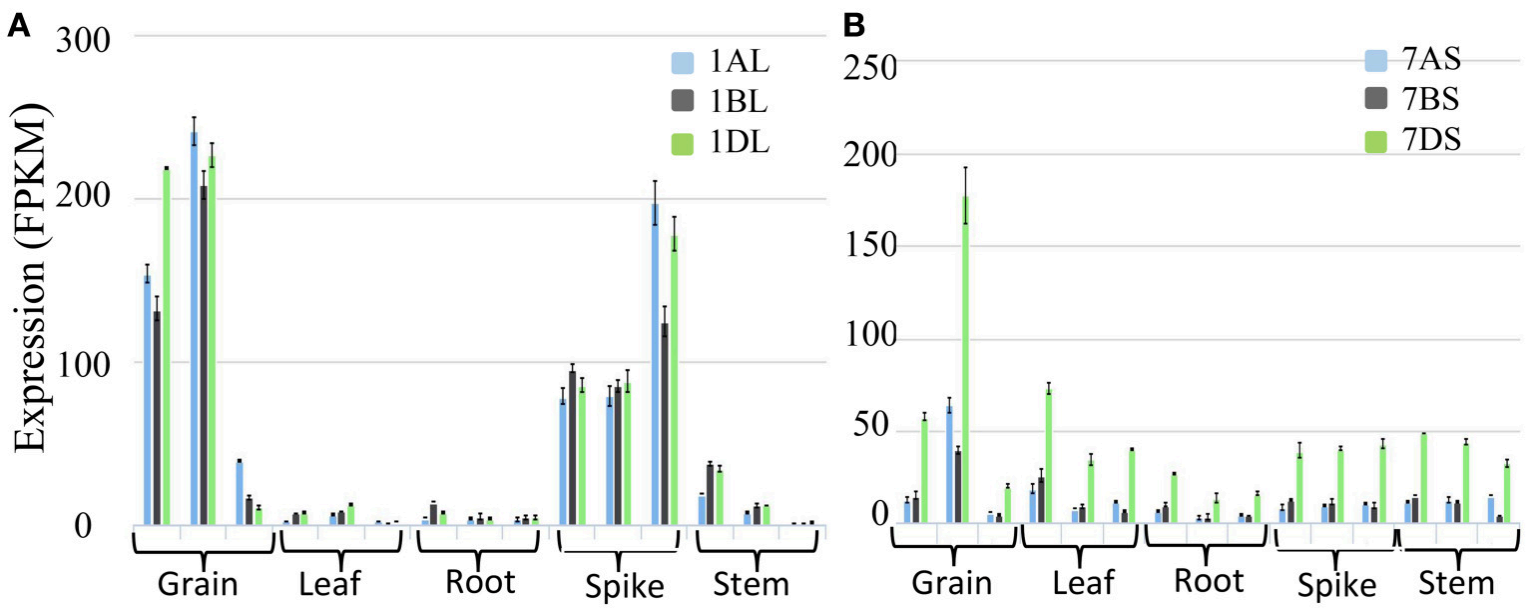
**Expression analysis based on wheat transcriptome data in different tissues (grain, leaf, root, spike and stem) and their development stages. (A)** Expression analysis of homoeologous genes for AGPase LS (Transcript id's: Traes_1AL_A1B2A8EB0.1, Traes_1BL_190920E1E.1 and Traes_1DL_844FE40E6.1) on group 1 chromosomes of wheat, and **(B)** Expression analysis of homoeologous genes for AGPase SS (Transcript id's: Traes_7AS_1B2A8C929.2, Traes_7BS_4FBE4B00A.2, and Traes_7DS_02539EB3B.1) on group 7 chromosomes of wheat.

Wheat LS homoeologues showed tissue specific and development stage specific expression. In the developing grains, the expression of the wheat LS homoeologues on 1AL and 1DL was significantly higher than that in 1BL. However, in the spike, the expression of all the three homoeologues was similar except at the later stages of spike development; where the expression of the gene on 1BL was significantly lower (Figure 11A). In case of SS, the highest expression was noticed in the developing grains, although the expression due to homoeologue on 7DS was distinctly higher relative to that of homoeologues on 7AS and 7BS (Figure 11B).

**Figure 12:**
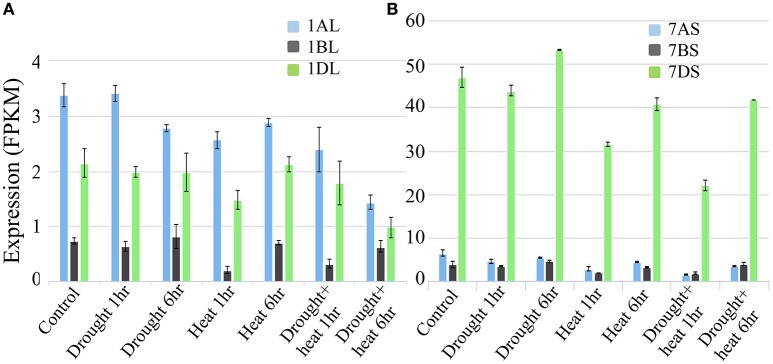
**Expression analysis based on wheat transcriptome data under heat and drought stress conditions. (A)** Expression analysis of homoeologous genes for AGPase LS (Transcript id's: Traes_1AL_A1B2A8EB0.1, Traes_1BL_190920E1E.1 and Traes_1DL_844FE40E6.1) on group 1 chromosomes of wheat **(B)** Expression analysis of homoeologous genes for AGPase SS (Transcript id's: Traes_7AS_1B2A8C929.2, Traes_7BS_4FBE4B00A.2, and Traes_7DS_02539EB3B.1) on group 7 chromosomes of wheat.

The microarray expression data revealed that under conditions of heat and drought stress, the expression of genes for both LS and SS was down-regulated in seedlings, mature leaves, and developing endosperms of several species; similar data was not available for wheat endosperm (data not presented). The expression analysis based on wheat transcriptome data confirmed the reduced expression of the LS and SS genes under drought and heat, although this decline was relatively more pronounced in LS (Figures 12A,B).

## Discussion

The present study demonstrated a high level of conservation of AGPase and its subunits (LS and SS) not only at the protein level, but also at the gene level in both monocots and dicots. At the protein level, this conservation was evident through estimates of similarity, identity, coverage of a sequence, the presence of two similar conserved domains (ADP_Glucose_PP and Lbh_G1P_AT_C) and 3D structure analysis across all the 11 species examined (8 monocots and 3 dicots). The data at the DNA level (gene sequence) suggested a higher level of conservation in exonic regions relative to that in introns, which is understandable, because introns are not involved in encoding the protein.

The level of conservation was higher for SS than for LS both at the protein and gene levels. This is in agreement with the results of an earlier study (Smith-White and Preiss, 1992). At the DNA level, value of Ka/Ks for SS was 2.61-fold that of LS, although the values of Ka/Ks for LS was higher in two earlier studies; Ka/Ks value in LS was 3.18-fold that in SS in one study (Corbi et al., 2012), and 2.7-fold in the other study (Georgelis et al., 2007, 2008), which may be attributed to the study of a larger set of species by the latter two studies than the present study. The higher similarity among SS than in LS may be due to its higher functional importance in the enzyme complex or due to a higher purifying selection exercised on SS during the evolution as suggested by Georgelis et al. (2007). Many more exons and introns, and relatively higher variability in their number in LS can be attributed to many more duplications in LS genes than in SS genes belonging to a number of eukaryotic plant systems (Georgelis et al., 2008), suggesting relatively higher conservation of SS than LS. Our analysis on intron phase gives further support to this conclusion. The prevalence of phase 0 in several genes was earlier attributed to primitive and conserved nature of the corresponding genes, since it allowed conservation of codons in the reading frame (Long and Deutsch, 1999; Figures 1, 2).

A comparison of intron length variation revealed that introns were generally longer in SS (34 to 3532 bp) than in LS (66 to 605 bp except third last intron of maize). Available evidence also suggested that during the course of evolution, division of introns in LS genes have given rise to smaller introns in monocots. For instance, splitting of sixth intron (537 bp) of LS on 1AL in *T. urartu*, the progenitor contributing A sub-genome to wheat, gave rise to a smaller sixth intron (102 bp) and two extra exons and introns (7 and 8) in wheat ortholog on chromosome arm 1AL. This variation in intron lengths may also be due to one or more of the following reasons: (*i*) presence of transposable elements, (*ii*) the changes in frequency and size of deletion events, and (*iii*) presence of regulatory elements and RNA genes (Zhu et al., 2009).

The low GC content in the introns than in the exons of genes for both subunits (LS and SS) was conspicuous, although it differed for the two subunits. Birdsell (2002) suggested that introns, intergenic regions and pseudogenes tend to have lower GC contents than ORFs. Differences in GC content allow discrimination between exons and introns and allow marking of exons for the splicing machinery (Amit et al., 2012). A positive correlation between the size of introns/exons (except introns of LS) with the GC content, observed in the present study, was in agreement with similar correlation reported in case of plant genomes of rice and *Arabidopsis* and the genomes of fly, zebrafish and worms (Zhu et al., 2009).

The sequences of genes for LS and SS of monocots and dicots differed, and seem to be more conserved in monocots. For instance, the number of exons and introns in monocots largely remained constant during evolution except in case of LS of *T. urartu* (15 exons in LS). However, variation seems to have occurred in dicots (12–14 exons in LS, 8–9 exons in SS), which was accompanied with loss of introns, leading to fusion of exons. It appears, that after the divergence of monocots and dicots from a common ancestor 140–150 MYA (Chaw et al., 2004), there was intron loss in dicots (Roy and Gilbert, 2006), which receives support from the fact that the exons were longer in dicots (Figures 1, 2). Dicot-monocot divergence is also apparent in our dendrogram, and in an earlier study (Rani et al., 2013). This is understandable since the domestication syndromes differ in monocots and dicots due to difference in the storage organs for starch. Higher variations in dicots also suggest a higher level of conservation in monocots than in dicots.

The lack of shared synteny (gene content) and collinearity (gene order) in most species except rice and sorghum suggest that the homoeologous chromosomes of different species carrying AGPase genes have undergone extensive reshuffling during evolution. The retention of some degree of synteny in rice and sorghum is understandable, since relative to other species, these are more closely related to maize (Salse et al., 2004). However, complete absence of shared synteny in other monocot species including *T. aestivum* (wheat), *T. urartu, Ae. tauschii*, barley and *Brachypodium* was a bit surprising, because at the whole genome level, extensive conservation of synteny has been reported among all grasses (Pfeifer et al., 2012).

Synteny and microsynteny information from the available literature (Ahn et al., 1993; Sorrells et al., 2003; Bennetzen and Chen, 2008) also suggest that although synteny at the whole genome level is conserved among grasses, but there are genomic regions, where synteny and microsynteny is lacking (Ahn et al., 1993; Tarchini et al., 2000; Vision, 2005; Bennetzen and Chen, 2008). This loss of shared synteny is attributed to gain/loss of genes and other structural changes within chromosome segments.

The superimposition of the heterotetrameric structures (predicted through MD simulation analyses) of each species over the known potato homotetramer (Jin et al., 2005) showed that the predicted heterotetramer structures are accurate and confirms identity of the true orthologs identified in the study. The rice heterotetrameric structure obtained during the present study appears to be better than the corresponding structure reported earlier (Dawar et al., 2013). The N-terminal region of the proteins for both subunits was hypervariable in all species examined. The N-terminal regions of SS in all dicots and in few monocots (*T. urartu*, wheat ortholog on 7BS, rice and sorghum) also contain a “QTCL” motif, which was earlier reported in potato and *Arabidopsis* (Linebarger et al., 2005; Hädrich et al., 2012). The absence of this motif in the remaining monocots suggested that this is present in plastidial AGPases only. It appears that after the divergence of monocots and dicots, this motif was retained by the plastidial copies of AGPases and was lost by the cytosolic copies since the motif is available in common ancestor (*Amborella trichopoda*) of monocots and dicots. Further study involving more dicot and monocot species may resolve whether or not this motif occurs more frequently in plastidial AGPase than in cytosolic AGPase. The important role of this motif in providing thermo-tolerance to AGPase was demonstrated in maize through the observation that QTCL motif was present in heat stable AGPase and not in heat labile AGPase (Hannah et al., 2001). The role of QTCL in providing heat stability was further substantiated through substitution of the “QTCL” motif for the native “STYL” motif through site directed mutagenesis, which provided heat stability (Linebarger et al., 2005).

It is known that the cysteine residue in the “QTCL” motif forms intermolecular SS disulphide bridge between the two small subunits (SS-SS dimerization), which facilitates the heterotetrameric structure of AGPase leading to its improved thermo-stability, albeit with reduced activity (Linebarger et al., 2005). The role of cysteine residue in SS-SS dimerization and in AGPase turnover was also reported in *Arabidopsis* (Hädrich et al., 2012).

In the present study, ligand-binding sites including the “LGGG” motif were identified in the ADP_Glucose_PP domain of the LS subunit in all of the species except *T. urartu*, the three wheat homoeologues, *Ae. tauschii* and chickpea. In all the three homoeologues of wheat and *Ae. tauschii*, the “LGGG” motif was present in the N-terminal region. The ATP, ADP, Mg^++^, 3-phosphoglyceric acid (3-PGA), fructose-6-phosphate (F-6-P) and glucose-6-phosphate (G-6-P) ligands are known to bind to the above sites and control the AGPase activity through allosteric and redox modifications (Boehlein et al., 2010). The occurrence of these identified ligand binding aa residues corresponds the similar lignad binding aa residues of potato homotetramer (1YP4), which interacts with ATP (Jin et al., 2005). Further, from among the several aa residues involved in the ligand binding sites, arginine has been implicated in controlling the allosteric regulation of maize AGPase (Boehlein et al., 2010). In an earlier study of SS of several monocot and dicot species, five times more ligand binding sites were reported in the ADP_Glucose_PP domain than in the LbH_G1P_AT_C domain (Sarma et al., 2014). Since ADP_Glucose_PP domain is mainly involved in catalyzing the first key rate-limiting step of starch biosynthesis in plants, the larger number of ligand binding sites in this particular domain may be contributing to its catalytic activity and may be involved in differential regulation of its enzymatic activity. However, the exact role of these residues may be elucidated through wet-lab experiments.

In promoter region, several cis-regulatory elements responsible for response to light, abiotic stress and endosperm expression were identified. Light responsive elements may have an important role, since starch is synthesized in the chloroplast of leaves during the day and degraded during the night following circadian rhythm. Thus, a tight regulation of pathways of starch synthesis and degradation in response to light signals is required. The response regulators in the LS and the SS 5′ regulatory regions may be responsible for the high level of expression of these genes in developing endosperm (Burton et al., 2002; Ohdan et al., 2005). Similarly in wheat LS, first 400 bp at the 5′ region along with the first intron of 88 bps was found to be important for its expression in the endosperm (Thorneycroft et al., 2003). The functional significance of the several other identified response elements in the present study need to be validated using wet lab approaches.

The higher expression of both the LS and the SS in developing endosperm during anthesis and at grain filling stage and low expression in the vegetative tissue (roots and leaves) revealed its role during the starch biosynthesis in developing grain. Similar results through RT-PCR analyses were reported in wheat endosperms-embryo, roots and leaves (Burton et al., 2002) and in the developing rice endosperm (Ohdan et al., 2005). However, in *Arabidopsis*, a higher expression of the AGPase genes was shown in leaves at the bolting stage, suggesting leaves to be the main organs for the carbohydrate accumulation (Streb and Zeeman, 2012), and not the grain endosperms as in the case of cereals.

The available data also suggested temporal expression of the wheat homoeologues and also down-regulation of both subunits of AGPase under conditions of abiotic stress (e.g., heat and drought) in the spikes and developing endosperm. However, similar data for expression of AGPase subunits in endosperm of cereals under abiotic stress was not available for any of the species studied. An earlier study involving RT-PCR analyses in wheat cv. Butte86 provided evidence of an early and sharp decline in the expression of AGPase and other starch synthesizing enzymes at a higher day temperature (37°C) (Hurkman et al., 2003). We are currently carrying out a detailed study of the expression of LS and SS in the developing wheat endosperm using a set of heat tolerant and sensitive genotypes under varying levels of heat stress. In the long run, this will allow us to develop gene-based functional markers for use in breeding programs for development of heat tolerant wheat cultivars.

## Conclusions

The present study identified the “true” orthologs of AGPase genes along with its structural and functional evolution among monocots and dicots. Overall, the comparative study revealed the structural conservation of LS and SS of AGPase, although some variation was observed in the length, number and phases of introns; N-terminal regions were relatively more variable. Among the two domains (ADP_Glucose_PP and LbH_G1P_AT_C) in each of the LS and SS, the domain ADP_Glucose_PP (with many more ligand binding sites carrying a conserved “LGGG” motif) perhaps plays an important role in the regulation of the AGPase activity. Some specific features of the LS and SS also suggested a species-specific evolution among monocots and dicots. Promoter and expression analyses revealed endosperm to be the main site of expression among cereals and leaves in *Arabidopsis*. Expression analysis also suggested that the expression of the genes for AGPases is regulated in a temporal manner during abiotic stresses.

## Author contributions

RB and GS performed the data analyses; MD simulation analysis was performed by SG and PV. AM assisted RB and GS in conducting the data analyses and preparing the first draft. KG, AM, HB, and PG conceived, supervised, edited, and finalized the manuscript.

## Funding

This work was supported by United States Agency for International Development, Feed the Future Innovation Lab-Climate Resilient Wheat (Grant number AID-OAA-A-13-00008).

### Conflict of interest statement

The authors declare that the research was conducted in the absence of any commercial or financial relationships that could be construed as a potential conflict of interest.
