# Supplementary material for: Comparative Analysis of AGPase Genes and Encoded Proteins in Eight Monocots and Three Dicots with Emphasis on Wheat
Source: Front Plant Sci. 2017 Jan 24;8:19. doi: 10.3389/fpls.2017.00019 (PMC5259687; doi:10.3389/fpls.2017.00019)
Supplement: Supplementary file 1 [file Table1.DOCX]

**Supplementary material**

**Comparative analysis of AGPase genes and encoded proteins in eight monocots and three dicots with emphasis on wheat**

Ritu Batra^1¶,^ Gautam Saripalli^1¶^, Amita Mohan^2^, Kulvinder S. Gill^2*^, Harindra Singh Balyan^1^ and Pushpendra Kumar Gupta^1^

*Correspondence:

Kulvinder S. Gill

email: [ksgill@wsu.edu](mailto:ksgill@wsu.edu)

Phone: 509-335-4666

**Supplementary Table 1:** Positions of exons (upper row) and introns (lower row) in base pairs in genes for AGPase LS in different species. The position of first exon is marked from translation start site.

| Species | Exon/Intron number | | | | | | | | | | | | | | |
| --- | --- | --- | --- | --- | --- | --- | --- | --- | --- | --- | --- | --- | --- | --- | --- |
|  | 1 | 2 | 3 | 4 | 5 | 6 | 7 | 8 | 9 | 10 | 11 | 12 | 13 | 14 | 15 |
| Maize | 1545-1745 | 2222-2344 | 2629-2799 | 3036-3125 | 3217-3303 | 3388-3443 | 3546-3639 | 3805-3917 | 3986-4058 | 4217-4297 | 4463-4549 | 4620-4724 | 6546-6652 | 6735-6795 | 6912-7013 |
|  | 1746-2221 | 2345-2628 | 2800-3035 | 3126-3216 | 3304-3387 | 3444-3545 | 3640-3804 | 3918-3985 | 4059-4216 | 4298-4462 | 4550-4619 | 4725-6545 | 6653-6734 | 6796-6911 |  |
| Wheat 1AL* | 1271-1492 | 1591-1710 | 2160-2330 | 2453-2542 | 2647-2733 | 2817-2872 | 2975-3068 | 3160-3272 | 3340-3412 | 3538-3618 | 3765-3851 | 3937-4041 | 4144-4250 | 4344-4404 | 4503-4604 |
|  | 1493-1590 | 1711-2159 | 2331-2452 | 2543-2646 | 2734-2816 | 2873-2974 | 3069-3159 | 3273-3339 | 3413-3537 | 3619-3764 | 3852-3936 | 4042-4143 | 4251-4343 | 4405-4502 |  |
| Wheat 1BL* | 4094-4315 | 4405-4524 | 5033-5203 | 5313-5402 | 5482-5568 | 5652-5707 | 5809-5902 | 6005-6117 | 6185-6257 | 6386-6466 | 6603-6689 | 6775-6879 | 6981-7087 | 7183-7243 | 7344-7444 |
|  | 4316-4404 | 4525-5032 | 5204-5312 | 5403-5481 | 5569-5651 | 5708-5808 | 5903-6004 | 6118-6184 | 6258-6385 | 6467-6602 | 6690-6774 | 6880-6980 | 7088-7182 | 7244-7342 |  |
| Wheat 1DL* | 710-931 | 1035-1154 | 1609-1779 | 1899-1988 | 2090-2176 | 2260-2315 | 2418-2511 | 2613-2725 | 2793-2865 | 2994-3074 | 3210-3296 | 3382-3486 | 3588-3694 | 3789-3849 | 3948-4049 |
|  | 932-1034 | 1155-1608 | 1780-1898 | 1989-2089 | 2177-2259 | 2316-2417 | 2512-2612 | 2726-2792 | 2866-2993 | 3075-3209 | 3297-3381 | 3487-3587 | 3695-3788 | 3850-3947 |  |
| *T. urartu* | 01-11 | 976-1033 | 1370-1489 | 1955-2125 | 2248-2337 | 2439-2525 | 2609-2664 | 3202-3274 | 3400-3480 | 3627-3713 | 3799-3903 | 4006-4112 | 4207-4267 | 4366-4635 | x |
|  | 12-975 | 1034-1369 | 1490-1954 | 2126-2247 | 2338-2438 | 2526-2608 | 2665-3201 | 3275-3399 | 3481-3626 | 3714-3798 | 3904-4005 | 4113-4206 | 4268-4365 | - |  |
| *Ae. Tauschii* | 1-213 | 317-436 | 891-1061 | 1181-1270 | 1372-1458 | 1542-1597 | 1700-1793 | 1895-2007 | 2075-2147 | 2276-2356 | 2465-2578 | 2664-2768 | 2875-2981 | 3076-3136 | 3235-3474 |
|  | 214-316 | 437-890 | 1062-1180 | 1271-1371 | 1459-1541 | 1598-1699 | 1794-1894 | 2008-2074 | 2148-2275 | 2357-2464 | 2579-2663 | 2769-2874 | 2982-3075 | 3137-3234 |  |
| *Brachypodium* | 1431-1652 | 1757-1876 | 2261-2431 | 2526-2615 | 2742-2828 | 2906-2961 | 3060-3153 | 3262-3373 | 3444-3516 | 3645-3725 | 3877-3963 | 4048-4152 | 4252-4358 | 4449-4509 | 4606-4707 |
|  | 1653-1756 | 1877-2260 | 2432-2525 | 2616-2741 | 2829-2905 | 2962-3059 | 3154-3261 | 3374-3443 | 3517-3644 | 3726-3876 | 3964-4047 | 4153-4251 | 4359-4448 | 4510-4605 |  |
| Rice | 3793-3999 | 4392-4514 | 5120-5290 | 5388-5477 | 5582-5668 | 5749-5804 | 5897-5990 | 6117-6229 | 6296-6368 | 6688-6768 | 6950-7036 | 7116-7220 | 7364-7470 | 7566-7626 | 7719-7820 |
|  | 4000-4391 | 4515-5119 | 5291-5387 | 5478-5581 | 5669-5748 | 5805-5896 | 5991-6116 | 6230-6295 | 6369-6687 | 6769-6949 | 7037-7115 | 7221-7363 | 7471-7565 | 7627-7718 |  |
| Barley | 668-892 | 1005-1124 | 1574-1744 | 1869-1958 | 2061-2147 | 2231-2286 | 2388-2481 | 2583-2695 | 2763-2835 | 2960-3040 | 3190-3276 | 3374-3478 | 3585-3691 | 3779-3839 | 3944-4045 |
|  | 893-1004 | 1125-1573 | 1745-1868 | 1959-2060 | 2148-2230 | 2287-2387 | 2482-2582 | 2696-2762 | 2836-2959 | 3041-3189 | 3277-3373 | 3479-3584 | 3692-3778 | 3840-3943 |  |
| Sorghum | 532-735 | 1216-1338 | 1603-1773 | 2005-2094 | 2186-2272 | 2356-2411 | 2515-2608 | 2775-2887 | 2956-3028 | 3198-3278 | 3456-3542 | 3622-3726 | 4028-4134 | 4236-4296 | 4395-4496 |
|  | 736-1215 | 1339-1602 | 1774-2004 | 2095-2185 | 2273-2355 | 2412-2514 | 2609-2774 | 2888-2955 | 2029-3197 | 3279-3455 | 3543-3621 | 3727-4027 | 4135-4235 | 4297-4394 |  |
| *Arabidopsis* | 346-558 | 1014-1127 | 1204-1377 | 1462-1553 | 1630-1717 | 1788-1840 | 1918-2011 | 2094-2279 | 2375-2455 | 2544-2630 | 2717-2989 | 3065-3166 | x | x | x |
|  | 559-1013 | 1128-1203 | 1378-1461 | 1554-1629 | 1718-1787 | 1841-1917 | 2012-2093 | 2280-2374 | 2456-2543 | 2631-2716 | 2990-3064 | - | - | - |  |
| Chickpea | 243-449 | 898-1020 | 1114-1284 | 1591-1683 | 1796-1879 | 1972-2027 | 2143-2236 | 2527-2712 | 2850-2930 | 3021-3107 | 3197-3301 | 3389-3495 | 3629-3689 | 3801-3902 | x |
|  | 450-897 | 1021-1113 | 1285-1590 | 1684-1795 | 1880-1971 | 2028-2142 | 2237-2526 | 2713-2849 | 2931-3020 | 3108-3196 | 3302-3388 | 3496-3628 | 3690-3800 | - |  |
| Potato | 657-890 | 1200-1322 | 1590-1760 | 1969-2061 | 2219-2302 | 2406-2461 | 2650-2743 | 2942-3127 | 3237-3321 | 3431-3517 | 3599-3703 | 3856-3962 | 4082-4142 | 4534-4635 | x |
|  | 891-1199 | 1323-1589 | 1761-1968 | 2062-2218 | 2303-2405 | 2462-2649 | 2744-2941 | 3128-3236 | 3322-3430 | 3518-3598 | 3704-3855 | 3963-4081 | 4143-4533 | - |  |

* indicates wheat homoeologues of group 1 chromosomes, x indicates absence of exons, - indicates absence of introns
