# Supplementary material for: Comparative Analysis of AGPase Genes and Encoded Proteins in Eight Monocots and Three Dicots with Emphasis on Wheat
Source: Front Plant Sci. 2017 Jan 24;8:19. doi: 10.3389/fpls.2017.00019 (PMC5259687; doi:10.3389/fpls.2017.00019)
Supplement: Supplementary file 2 [file Table2.DOCX]

**Supplementary material**

**Comparative analysis of AGPase genes and encoded proteins in eight monocots and three dicots with emphasis on wheat**

Ritu Batra^1¶,^ Gautam Saripalli^1¶^, Amita Mohan^2^, Kulvinder S. Gill^2*^, Harindra Singh Balyan^1^ and Pushpendra Kumar Gupta^1^

*Correspondence:

Kulvinder S. Gill

email: [ksgill@wsu.edu](mailto:ksgill@wsu.edu)

Phone: 509-335-4666

**Supplementary Table 2**: Percent similarity of exons (upper row) and introns (lower low) in genes for AGPase LS in different species with respect to exons of gene for AGPase LS of maize

| Species | Exon/Intron number | | | | | | | | | | | | | | |
| --- | --- | --- | --- | --- | --- | --- | --- | --- | --- | --- | --- | --- | --- | --- | --- |
|  | 1 | 2 | 3 | 4 | 5 | 6 | 7 | 8 | 9 | 10 | 11 | 12 | 13 | 14 | 15 |
| Wheat 1AL* | 44.97 | 69.17 | 79.76 | 81.11 | 80.23 | 84.21 | 71.58 | 70.18 | 80 | 77.22 | 78.41 | 69.23 | 64.29 | 77.05 | 76.92 |
|  | 35.11 | 39.15 | 50.82 | 36.21 | 54.22 | 52.63 | 47.06 | 53.12 | 45.54 | 35.07 | 31.03 | 53.92 | 14.81 | 42.86 |  |
| Wheat 1BL* | 43.43 | 67.5 | 79.76 | 80 | 80.23 | 84.21 | 69.47 | 71.05 | 77.14 | 77.22 | 75 | 69.23 | 64.29 | 77.05 | 75.96 |
|  | 35.29 | 40.32 | 55.05 | 33.33 | 53.01 | 53.13 | 42.62 | 53.12 | 45.22 | 35.66 | 37.93 | 54.46 | 25.93 | 48.94 |  |
| Wheat 1DL* | 44.44 | 67.5 | 79.17 | 78.89 | 80.23 | 84.21 | 70.53 | 69.3 | 77.14 | 75.95 | 76.14 | 69.23 | 64.29 | 77.05 | 75.96 |
|  | 31.11 | 36 | 52.1 | 39.66 | 53.01 | 53.68 | 45.9 | 53.12 | 45.22 | 34.38 | 31.03 | 53.47 | 18.52 | 39.8 |  |
| *T. urartu* | 63.64 | 50 | 69.17 | 79.76 | 81.11 | 80.23 | 84.21 | 80 | 77.28 | 78.41 | 68.27 | 64.29 | 78.69 | 75 | x |
|  | 37.35 | 38.46 | 52.46 | 37.93 | 50.6 | 51.49 | 37.93 | 44.12 | 40.43 | 32 | 50 | 56.12 | - | - |  |
| *Ae. tauschii* | 43.92 | 67.5 | 80.36 | 78.79 | 80.23 | 84.21 | 70.53 | 69.3 | 77.14 | 76.25 | 76.14 | 68.27 | 64.29 | 77.05 | 75 |
|  | 31.11 | 36 | 52.1 | 37.93 | 50.6 | 53.68 | 44.26 | 53.12 | 45.22 | 30.39 | 31.03 | 52.83 | 18.52 | 42.86 |  |
| *Brachypodium* | 53.81 | 71.67 | 82.74 | 83.83 | 79.07 | 85.96 | 70.53 | 73.68 | 80 | 79.75 | 78.41 | 69.23 | 66.67 | 77.05 | 76.92 |
|  | 38 | 38.18 | 42.55 | 48.05 | 46.75 | 56.52 | 35.82 | 55.22 | 45.3 | 33.81 | 23 | 48.48 | 37.04 | 33.33 |  |
| Rice | 73.13 | 82.93 | 88.1 | 78.89 | 83.33 | 82.46 | 78.75 | 82.46 | 80.28 | 86.25 | 80.68 | 77.14 | 73.68 | 83.61 | 87.5 |
|  | 43.55 | 41.28 | 56.25 | 47.5 | 58.97 | 56.04 | 54.4 | 56.92 | 53.02 | 45.4 | 49.23 | 55.94 | 27.59 | 39.13 |  |
| Barley | 43.75 | 67.5 | 79.76 | 88.89 | 80.23 | 84.21 | 68.42 | 71.93 | 78.57 | 76.25 | 78.41 | 68.27 | 62.5 | 77.05 | 76.92 |
|  | 33.33 | 40 | 55.65 | 38.98 | 53.01 | 53.19 | 42.62 | 54.69 | 43.24 | 34.81 | 32.76 | 52.83 | 22.22 | 42.31 |  |
| Sorghum | 93.53 | 95.93 | 96.43 | 94.51 | 94.19 | 94.74 | 93.68 | 93.86 | 95.77 | 96.25 | 94.32 | 95.24 | 85.96 | 96.72 | 94.23 |
|  | 89.43 | 79.85 | 82.46 | 94.51 | 90.36 | 88.24 | 78.79 | 94.12 | 90.51 | 77.58 | 74.29 | 71.63 | 69.14 | 61.22 |  |
| *Arabidopsis* | 41.38 | 61.06 | 70.24 | 61.11 | 61.73 | 71.93 | 69.47 | 58.77 | 69.01 | 77.5 | 75 | 61.54 | 61.4 | 75.41 | 62.75 |
|  | 42.42 | 45.45 | 36.21 | 44.64 | 43.48 | 50 | 53.85 | 40 | 31.17 | 52.38 | 53.85 | - | - | - |  |
| Chickpea | 38.93 | 56.56 | 74.4 | 65.56 | 51.79 | 67.86 | 63.16 | 64.04 | 64.79 | 69.23 | 68.97 | 60.19 | 70.18 | 73.77 | 70.59 |
|  | 38.46 | 59.55 | 45.22 | 52.75 | 44 | 36.36 | 41.21 | 36.36 | 29.17 | 48.24 | 28 | 54.14 | 50.7 | - |  |
| Potato | 36.47 | 52.85 | 74.4 | 68.89 | 62.65 | 73.21 | 67.37 | 64.04 | 59.15 | 75.64 | 68.97 | 58.1 | 59.65 | 72.13 | 66.67 |
|  | 40.82 | 49.57 | 39.18 | 49.45 | 49.4 | 50 | 46.3 | 42.86 | 15.38 | 40.54 | 44.29 | 53.78 | 43.9 | - |  |

* indicates wheat homoeologues of group 1 chromosomes, x indicates absence of exons, - indicates absence of introns
