# Supplementary material for: Comparative Analysis of AGPase Genes and Encoded Proteins in Eight Monocots and Three Dicots with Emphasis on Wheat
Source: Front Plant Sci. 2017 Jan 24;8:19. doi: 10.3389/fpls.2017.00019 (PMC5259687; doi:10.3389/fpls.2017.00019)
Supplement: Supplementary file 3 [file Table3.DOCX]

**Supplementary material**

**Comparative analysis of AGPase genes and encoded proteins in eight monocots and three dicots with emphasis on wheat**

Ritu Batra^1¶,^ Gautam Saripalli^1¶^, Amita Mohan^2^, Kulvinder S. Gill^2*^, Harindra Singh Balyan^1^ and Pushpendra Kumar Gupta^1^

*Correspondence: Kulvinder S. Gill: [ksgill@wsu.edu](mailto:ksgill@wsu.edu)

**Supplementary Table 3**: Positions of exons (upper row) and introns (lower row) in base pairs in genes for AGPase SS in different species. The position of first exon is marked from translation start site.

| Species | Exon/Intron number | | | | | | | | | | |
| --- | --- | --- | --- | --- | --- | --- | --- | --- | --- | --- | --- |
|  | 1 | 2 | 3 | 4 | 5 | 6 | 7 | 8 | 9 | 10 | 11 |
| Maize | 6-134 | 1497-1793 | 3007-3276 | 3673-3852 | 4144-4247 | 4365-4476 | 4597-4695 | 4772-4891 | 5481-5597 | x | x |
|  | 135-1496 | 1794-3006 | 3277-3672 | 3853-4143 | 4248-4364 | 4477-4596 | 4696-4771 | 4892-5480 | - | - |  |
| Wheat 7AS* | 121-243 | 3776-4072 | 4980-5249 | 5503-5682 | 5882-5985 | 6097-6208 | 6422-6520 | 6591-6710 | 7535-7651 | x | x |
|  | 244-3775 | 4073-4979 | 5250-5502 | 5683-5881 | 5986-6096 | 6209-6421 | 6521-6590 | 6711-7534 | - | - |  |
| Wheat 7BS* | 183-246 | 1533-1829 | 2738-3007 | 3261-3440 | 3641-3744 | 3853-3964 | 4178-4276 | 4347-4466 | 5291-5408 | x | x |
|  | 247-1532 | 1830-2737 | 3008-3260 | 3441-3640 | 3745-3852 | 3965-4177 | 4277-4346 | 4467-5291 | - | - |  |
| Wheat 7DS* | 965-1210 | 2310-2606 | 3516-3785 | 4039-4218 | 4402-4505 | 4617-4728 | 4938-5036 | 5107-5226 | 6053-6169 | x | x |
|  | 1211-2309 | 2607-3515 | 3786-4038 | 4219-4401 | 4506-4616 | 4729-4937 | 5037-5106 | 5227-6052 | - | - |  |
| *T. urartu* | 1-246 | 2352-2648 | 3556-3825 | 4079-4258 | 4458-4561 | 4673-4784 | 4998-5096 | 5167-5286 | 5606-5784 | 6327-6399 | 6434-6514 |
|  | 247-2351 | 2649-3555 | 3826-4078 | 4259-4457 | 4562-4672 | 4785-4997 | 5097-5166 | 5287-5605 | 5785-6326 | 6400-6433 |  |
| *Ae. tauschii* | 1-123 | 2208-2504 | 3414-3683 | 3937-4116 | 4300-4403 | 4515-4626 | 4836-4934 | 5005-5124 | 5951-6067 | x | x |
|  | 124-2207 | 2505-3413 | 3684-3936 | 4117-4299 | 4404-4514 | 4627-4835 | 4935-5004 | 5125-5950 | - | - |  |
| *Brachypodium* | 1-126 | 1688-1984 | 2696-2965 | 3249-3428 | 3621-3724 | 3843-3954 | 4176-4274 | 4350-4469 | 4706-4822 | x | x |
|  | 127-1687 | 1985-2695 | 2966-3248 | 3429-3620 | 3725-3842 | 3955-4175 | 4275-4349 | 4470-4705 | - | - |  |
| Rice | 1665-1910 | 2517-2813 | 3462-3730 | 3847-4026 | 4214-4317 | 4429-4540 | 4663-4761 | 4847-4966 | 5626-5742 | x | x |
|  | 1911-2516 | 2814-3461 | 3731-3846 | 4027-4213 | 4318-4428 | 4541-4662 | 4762-4846 | 4967-5625 | - | - |  |
| Barley | 241-360 | 3075-3471 | 4376-4645 | 4899-5078 | 6987-7090 | 7202-7313 | 7528-7626 | 7697-7816 | 9025-9141 | x | x |
|  | 361-3174 | 3472-4375 | 4646-4898 | 5079-6986 | 7091-7201 | 7314-7527 | 7627-7696 | 7817-9024 | - | - |  |
| Sorghum | 161-394 | 976-1272 | 1894-2163 | 2350-2529 | 2711-2814 | 2912-3023 | 3234-3332 | 3413-3532 | 3952-4068 | x | x |
|  | 395-975 | 1273-1893 | 2164-2349 | 2530-2710 | 2815-2911 | 3024-3233 | 3333-3412 | 3533-3951 | - | - |  |
| *Arabidopsis* | 87-350 | 450-1016 | 1096-1275 | 1435-1538 | 1612-1723 | 1803-1901 | 1989-2108 | 2202-2565 | x | x | x |
|  | 351-449 | 1017-1095 | 1276-1434 | 1539-1611 | 1724-1802 | 1902-1988 | 2109-2201 |  | - | - |  |
| Chickpea | 71-331 | 611-907 | 1280-1549 | 1629-1808 | 1896-1999 | 2078-2189 | 2269-2367 | 2456-2575 | 2665-2781 | x | x |
|  | 332-610 | 908-1279 | 1550-1628 | 1809-1895 | 2000-2077 | 2190-2268 | 2368-2455 | 2576-2664 | - | - |  |
| Potato | 836-1102 | 1981-2277 | 2562-2831 | 2925-3104 | 3504-3607 | 3713-3824 | 4716-4814 | 5042-5161 | 5357-5473 | x | x |
|  | 1103-1980 | 2278-2561 | 2832-2924 | 3105-3503 | 3608-3712 | 3825-4715 | 4815-5041 | 5162-5356 | - | - |  |

* indicates wheat homoeologues of group 7 chromosomes, x indicates absence of exons, - indicates absence of introns
