# Supplementary material for: Comparative Analysis of AGPase Genes and Encoded Proteins in Eight Monocots and Three Dicots with Emphasis on Wheat
Source: Front Plant Sci. 2017 Jan 24;8:19. doi: 10.3389/fpls.2017.00019 (PMC5259687; doi:10.3389/fpls.2017.00019)
Supplement: Supplementary file 4 [file Table4.DOCX]

**Supplementary material**

**Comparative analysis of AGPase genes and encoded proteins in eight monocots and three dicots with emphasis on wheat**

Ritu Batra^1¶,^ Gautam Saripalli^1¶^, Amita Mohan^2^, Kulvinder S. Gill^2*^, Harindra Singh Balyan^1^ and Pushpendra Kumar Gupta^1^

*Correpondence: Kulvinder S. Gill: [ksgill@wsu.edu](mailto:ksgill@wsu.edu)

**Supplementary Table 4**: Percent similarity of exons (upper row) and introns (lower low) in genes for AGPase SS in different species with respect to exons and introns of gene for AGPase SS of maize

| Species | Exon /Intron number | | | | | | | | |
| --- | --- | --- | --- | --- | --- | --- | --- | --- | --- |
|  | 1 | 2 | 3 | 4 | 5 | 6 | 7 | 8 | 9 |
| Wheat 7AS* | 53.66 | 89.12 | 90.41 | 89.44 | 87.13 | 94.64 | 89.90 | 86.87 | 89.74 |
|  | 49.92 | 45.52 | 56.91 | 53.37 | 64.22 | 60.83 | 64.18 | 48.96 |  |
| Wheat 7BS* | 44.96 | 89.26 | 91.14 | 90.00 | 87.13 | 93.75 | 89.90 | 85.83 | 89.74 |
|  | 46.29 | 46.03 | 56.10 | 53.09 | 63.96 | 60.83 | 59.70 | 48.53 |  |
| Wheat 7DS* | 35.71 | 89.60 | 90.41 | 90.56 | 86.14 | 94.64 | 89.90 | 86.87 | 89.74 |
|  | 41.47 | 41.86 | 55.87 | 49.66 | 62.16 | 59.29 | 41.94 | 49.73 |  |
| *T. urartu* | 35.11 | 89.26 | 91.14 | 90.00 | 87.13 | 94.64 | 89.90 | 86.86 | 9.32 |
|  | 42.47 | 41.49 | 55.47 | 49.07 | 61.26 | 61.74 | 43.55 | 38.64 |  |
| *Ae. tauschii* | 53.66 | 89.60 | 90.41 | 90.56 | 86.14 | 94.64 | 89.90 | 86.87 | 90.60 |
|  | 44.78 | 41.86 | 55.47 | 49.66 | 62.16 | 59.29 | 41.94 | 39.55 |  |
| *Brachypodium* | 53.97 | 89.26 | 92.62 | 90.00 | 86.14 | 89.29 | 89.90 | 85.83 | 86.32 |
|  | 47.52 | 45.12 | 51.10 | 47.01 | 61.54 | 54.62 | 55.88 | 51.98 |  |
| Rice | 35.19 | 89.60 | 90.41 | 91.11 | 87.13 | 92.86 | 82.83 | 84.17 | 87.18 |
|  | 48.68 | 46.70 | 59.09 | 47.24 | 44.95 | 60.40 | 47.37 | 48.93 |  |
| Barley | 46.67 | 88.59 | 90.77 | 88.89 | 85.15 | 93.75 | 90.91 | 87.50 | 88.89 |
|  | 44.82 | 41.79 | 54.66 | 47.64 | 60.36 | 60.87 | 38.71 | 41.12 |  |
| Sorghum | 37.84 | 83.22 | 84.13 | 83.33 | 86.00 | 91.96 | 81.82 | 80.83 | 81.20 |
|  | - | 43.55 | 40.56 | 46.34 | 47.23 | 41.23 | -- | 49.40 |  |
| *Arabidopsis* | 48.00 | 76.17 | 79.70 | 77.78 | 81.19 | 77.68 | 77.78 | 68.33 | 69.23 |
|  | 26.67 | 53.16 | 32.03 | 30.00 | 34.67 | 45.45 | -- | - |  |
| Chickpea | 37.60 | 77.85 | 79.34 | 75.56 | 80.39 | 75.00 | 74.75 | 75.83 | 80.34 |
|  | 44.11 | 45.89 | 35.90 | 56.25 | 43.59 | 51.72 | 24.07 | 40.45 |  |
| Potato | 37.30 | 75.84 | 83.39 | 74.44 | 79.41 | 79.46 | 74.75 | 69.17 | 76.92 |
|  | 42.29 | 46.01 | 52.69 | 43.28 | 43.28 | 35.59 | 30.30 | 46.86 |  |
