# Supplementary material for: Comparative Analysis of AGPase Genes and Encoded Proteins in Eight Monocots and Three Dicots with Emphasis on Wheat
Source: Front Plant Sci. 2017 Jan 24;8:19. doi: 10.3389/fpls.2017.00019 (PMC5259687; doi:10.3389/fpls.2017.00019)
Supplement: Supplementary file 5 [file Table5.DOCX]

**Supplementary material**

**Comparative analysis of AGPase genes and encoded proteins in eight monocots and three dicots with emphasis on wheat**

Ritu Batra^1¶,^ Gautam Saripalli^1¶^, Amita Mohan^2^, Kulvinder S. Gill^2*^, Harindra Singh Balyan^1^ and Pushpendra Kumar Gupta^1^

*Correspondence:

Kulvinder S. Gill

email: [ksgill@wsu.edu](mailto:ksgill@wsu.edu)

Phone: 509-335-4666

**Supplementary Table 5:** Ka (upper row) and Ks (lower row) values in genes for AGPase LS in monocots

| Species | Maize | Wheat 1AL | Wheat 1BL | Wheat 1DL | *T. urartu* | *Ae. tauschii* | *Brachypodium* | Rice | Barley | Sorghum | Average value of Ka/Ks |
| --- | --- | --- | --- | --- | --- | --- | --- | --- | --- | --- | --- |
| Maize | 0 |  |  |  |  |  |  |  |  |  |  |
|  | 0 |  |  |  |  |  |  |  |  |  |  |
| Wheat 1AL * | 0.246 | 0 |  |  |  |  |  |  |  |  |  |
|  | 0.093 | 0 |  |  |  |  |  |  |  |  |  |
| Wheat 1BL* | 0.262 | 0.018 | 0 |  |  |  |  |  |  |  |  |
|  | 0.088 | 0 | 0 |  |  |  |  |  |  |  |  |
| Wheat 1DL* | 0.257 | 0.014 | 0.004 | 0 |  |  |  |  |  |  |  |
|  | 0.088 | 0 | 0 | 0 |  |  |  |  |  |  |  |
| *T. urartu* | 0.255 | 0.007 | 0.025 | 0.022 | 0 |  |  |  |  |  |  |
|  | 0.105 | 0.010 | 0.010 | 0.010 | 0 |  |  |  |  |  |  |
| *Ae. tauschii* | 0.247 | 0.014 | 0.011 | 0.007 | 0.022 | 0 |  |  |  |  |  |
|  | 0.088 | 0 | 0 | 0 | 0.010 | 0 |  |  |  |  |  |
| *Brachypodium* | 0.227 | 0.095 | 0.107 | 0.103 | 0.103 | 0.103 | 0 |  |  |  |  |
|  | 0.076 | 0.010 | 0.010 | 0.010 | 0.021 | 0.010 | 0 |  |  |  |  |
| Rice | 0.169 | 0.273 | 0.267 | 0.272 | 0.283 | 0.272 | 0.219 | 0 |  |  |  |
|  | 0.092 | 0.121 | 0.122 | 0.122 | 0.133 | 0.122 | 0.106 | 0 |  |  |  |
| Barley | 0.250 | 0.026 | 0.029 | 0.025 | 0.029 | 0.025 | 0.103 | 0.275 | 0 |  |  |
|  | 0.080 | 0 | 0 | 0 | 0.010 | 0 | 0.010 | 0.116 | 0 |  |  |
| Sorghum | 0.054 | 0.240 | 0.246 | 0.242 | 0.250 | 0.232 | 0.214 | 0.171 | 0.244 | 0 |  |
|  | 0.037 | 0.069 | 0.064 | 0.064 | 0.081 | 0.064 | 0.048 | 0.100 | 0.059 | 0 | 2.92 |

*indicates wheat homoeologues of group 1 chromosomes, Ka- Non-synonymous substitutions, Ks-Synonymous substitutions
