# Supplementary material for: Comparative Analysis of AGPase Genes and Encoded Proteins in Eight Monocots and Three Dicots with Emphasis on Wheat
Source: Front Plant Sci. 2017 Jan 24;8:19. doi: 10.3389/fpls.2017.00019 (PMC5259687; doi:10.3389/fpls.2017.00019)
Supplement: Supplementary file 6 [file Table6.DOCX]

**Supplementary material**

**Comparative analysis of AGPase genes and encoded proteins in eight monocots and three dicots with emphasis on wheat**

Ritu Batra^1¶,^ Gautam Saripalli^1¶^, Amita Mohan^2^, Kulvinder S. Gill^2*^, Harindra Singh Balyan^1^ and Pushpendra Kumar Gupta^1^

*Correspondence:

Kulvinder S. Gill

email: [ksgill@wsu.edu](mailto:ksgill@wsu.edu)

Phone: 509-335-4666

**Supplementary Table 6:** Ka (upper row) and Ks (lower row) values in genes for AGPase LS in dicots

| Species | *Arabidopsis* | Chickpea | Potato | Average value of Ka/Ks |
| --- | --- | --- | --- | --- |
| *Arabidopsis* | 0 |  |  |  |
|  | 0 |  |  |  |
| Chickpea | 0.417 | 0 |  |  |
|  | 0.661 | 0 |  |  |
| Potato | 0.480 | 0.326 | 0 |  |
|  | 0.673 | 0.422 | 0 | 0.697 |

Ka- Non-synonymous substitutions; Ks-Synonymous substitutions
