# Supplementary material for: Comparative Analysis of AGPase Genes and Encoded Proteins in Eight Monocots and Three Dicots with Emphasis on Wheat
Source: Front Plant Sci. 2017 Jan 24;8:19. doi: 10.3389/fpls.2017.00019 (PMC5259687; doi:10.3389/fpls.2017.00019)
Supplement: Supplementary file 7 [file Table7.DOCX]

**Supplementary material**

**Comparative analysis of AGPase genes and encoded proteins in eight monocots and three dicots with emphasis on wheat**

Ritu Batra^1¶,^ Gautam Saripalli^1¶^, Amita Mohan^2^, Kulvinder S. Gill^2*^, Harindra Singh Balyan^1^ and Pushpendra Kumar Gupta^1^

*Correspondence:

Kulvinder S. Gill

email: [ksgill@wsu.edu](mailto:ksgill@wsu.edu)

Phone: 509-335-4666

**Supplementary Table 7:** Ka (upper row) and Ks (lower row) values in genes for AGPase SS in monocots

| Species | Maize | Wheat 7AS | Wheat 7BS | Wheat 7DS | *T. urartu* | *Ae. tauschii* | *Brachypodium* | Rice | Barley | Sorghum | Average value of Ka/KS |
| --- | --- | --- | --- | --- | --- | --- | --- | --- | --- | --- | --- |
| Maize | 0 |  |  |  |  |  |  |  |  |  |  |
|  | 0 |  |  |  |  |  |  |  |  |  |  |
| Wheat 7AS* | 0.127 | 0 |  |  |  |  |  |  |  |  |  |
|  | 0.019 | 0 |  |  |  |  |  |  |  |  |  |
| Wheat 7BS* | 0.127 | 0.009 | 0 |  |  |  |  |  |  |  |  |
|  | 0.019 | 0 | 0 |  |  |  |  |  |  |  |  |
| Wheat 7DS* | 0.127 | 0.006 | 0.009 | 0 |  |  |  |  |  |  |  |
|  | 0.019 | 0 | 0 | 0 |  |  |  |  |  |  |  |
| *T. urartu* | 0.123 | 0.003 | 0.009 | 0.006 | 0 |  |  |  |  |  |  |
|  | 0.019 | 0 | 0 | 0 | 0 |  |  |  |  |  |  |
| *Ae. tauschii* | 0.125 | 0.004 | 0.007 | 0.004 | 0.004 | 0 |  |  |  |  |  |
|  | 0.019 | 0 | 0 | 0 | 0 | 0 |  |  |  |  |  |
| *Brachypodium* | 0.131 | 0.082 | 0.083 | 0.082 | 0.080 | 0.080 | 0 |  |  |  |  |
|  | 0.033 | 0.014 | 0.014 | 0.014 | 0.014 | 0.014 | 0 |  |  |  |  |
| Rice | 0.146 | 0.127 | 0.131 | 0.131 | 0.123 | 0.129 | 0.133 | 0 |  |  |  |
|  | 0.029 | 0.029 | 0.029 | 0.029 | 0.029 | 0.029 | 0.033 | 0 |  |  |  |
| Barley | 0.131 | 0.021 | 0.024 | 0.021 | 0.018 | 0.020 | 0.087 | 0.132 | 0 |  |  |
|  | 0.019 | 0 | 0 | 0 | 0 | 0 | 0.014 | 0.029 | 0 |  |  |
| Sorghum | 0.223 | 0.205 | 0.208 | 0.205 | 0.201 | 0.203 | 0.209 | 0.221 | 0.206 | 0 |  |
|  | 0.049 | 0.046 | 0.049 | 0.046 | 0.047 | 0.046 | 0.053 | 0.017 | 0.046 | 0 | 5.263 |

* indicates wheat homoeologues of group 7 chromosomes, Ka- Non-synonymous substitutions, Ks-Synonymous substitutions
