# Supplementary material for: Comparative Analysis of AGPase Genes and Encoded Proteins in Eight Monocots and Three Dicots with Emphasis on Wheat
Source: Front Plant Sci. 2017 Jan 24;8:19. doi: 10.3389/fpls.2017.00019 (PMC5259687; doi:10.3389/fpls.2017.00019)
Supplement: Supplementary file 9 [file Table9.DOCX]

**Supplementary material**

**Comparative analysis of AGPase genes and encoded proteins in eight monocots and three dicots with emphasis on wheat**

Ritu Batra^1¶,^ Gautam Saripalli^1¶^, Amita Mohan^2^, Kulvinder S. Gill^2*^, Harindra Singh Balyan^1^ and Pushpendra Kumar Gupta^1^

*Correspondence:

Kulvinder S. Gill

email: [ksgill@wsu.edu](mailto:ksgill@wsu.edu)

Phone: 509-335-4666

**Supplementary Table 9**: Simple sequence repeats (SSRs), retro-elements and transposons identified in gene for AGPase LS in 7 species

| Species | Position in bp | Type | Number | Size(bp) |
| --- | --- | --- | --- | --- |
| Maize | 4795-5527; 5767-6216 | LINEs | 2 | 1183 |
|  | 6407-6428 | SSR | 1 | 22 |
| *Brachypodium* | 4923-4942 | SSR | 1 | 20 |
| Rice | 687-713 | SSR | 1 | 27 |
| Barley | 1296-1435; 1937-2206 | Transposons | 2 | 410 |
|  | 2994-3094 | LINE | 1 | 101 |
|  | 3287-4017 | LTR element | 1 | 731 |
| *Arabidopsis* | 83-119 | SSR | 1 | 38 |
| Chickpea | 849-893 | SSR | 1 | 46 |
| Potato | 2336-2366 | SSR | 1 | 31 |
