# Supplementary material for: Comparative Analysis of AGPase Genes and Encoded Proteins in Eight Monocots and Three Dicots with Emphasis on Wheat
Source: Front Plant Sci. 2017 Jan 24;8:19. doi: 10.3389/fpls.2017.00019 (PMC5259687; doi:10.3389/fpls.2017.00019)
Supplement: Supplementary file 10 [file Table10.DOCX]

**Supplementary material**

**Comparative analysis of AGPase genes and encoded proteins in eight monocots and three dicots with emphasis on wheat**

Ritu Batra^1¶,^ Gautam Saripalli^1¶^, Amita Mohan^2^, Kulvinder S. Gill^2*^, Harindra Singh Balyan^1^ and Pushpendra Kumar Gupta^1^

*Correspondence:

Kulvinder S. Gill

email: [ksgill@wsu.edu](mailto:ksgill@wsu.edu)

Phone: 509-335-4666

**Supplementary Table 10**: Simple sequence repeats (SSRs), retro-elements and transposons identified in gene for AGPase SS in 7 species

| Species | Position in bp | Type | Number | Size (bp) |
| --- | --- | --- | --- | --- |
| Maize | 2833-2906; 3126-3351 | LINEs | 2 |  |
| Wheat 7BS* | 4775-4991 | LTR element | 1 | 217 |
| Wheat 7DS* | 741-799 | SSR | 1 | 52 |
| *Ae. tauschii* | 643-701; 990-1034 | SSRs | 2 | 104 |
| Rice | 1542-1596; 1732-1756 | SSRs | 2 | 80 |
| Barley | 227-257 | SSR | 1 | 31 |
| Sorghum | 3893-4014 | Transposon | 1 | 122 |
|  | 96-126; 380-421 | SSRs | 2 | 73 |
| Chickpea | 108-134; 544-595; 2011-2065 | SSRs | 3 | 134 |
| Potato | 3791-3942 | SINE | 1 | 152 |
|  | 4019-4155; 4231-4324; 4668-4752; 4828-4921 | Transposons | 4 | 410 |

* indicates wheat homoeologues of group 7 chromosomes
