# Supplementary material for: Comparative Analysis of AGPase Genes and Encoded Proteins in Eight Monocots and Three Dicots with Emphasis on Wheat
Source: Front Plant Sci. 2017 Jan 24;8:19. doi: 10.3389/fpls.2017.00019 (PMC5259687; doi:10.3389/fpls.2017.00019)
Supplement: Supplementary file 11 [file Table11.DOCX]

**Supplementary material**

**Comparative analysis of AGPase genes and encoded proteins in eight monocots and three dicots with emphasis on wheat**

Ritu Batra^1¶,^ Gautam Saripalli^1¶^, Amita Mohan^2^, Kulvinder S. Gill^2*^, Harindra Singh Balyan^1^ and Pushpendra Kumar Gupta^1^

*Correspondence:

Kulvinder S. Gill

email: [ksgill@wsu.edu](mailto:ksgill@wsu.edu)

Phone: 509-335-4666

**Supplementary Table 11**: Details of 3D structure and quality assessment parameters of AGPase LS (oligostate-monomer) (upper row) and AGPase SS (oligostate-homotetramer) (lower row) in all the monocots and dicots obtained using Swiss-Model

| Species | Template used | % Identity | GMQE score | Quality assessment parameters | |
| --- | --- | --- | --- | --- | --- |
|  |  |  |  | QMEAN  6 score | Dfire  energy |
| Maize | 1yp2 | 50.58 | 0.69 | 0.665 | -584.17 |
|  | 1yp2 | 89.19 | 0.88 | 0.715 | -2654.72 |
| Wheat1AL* | 1yp2 | 51.08 | 0.74 | 0.705 | -599.14 |
| Wheat 7AS** | 1yp2 | 92.38 | 0.91 | 0.713 | -2541.41 |
| Wheat 1BL* | 1yp2 | 51.31 | 0.70 | 0.673 | -539.33 |
| Wheat 7BS** | 1yp2 | 92.38 | 0.91 | 0.713 | -2541.41 |
| Wheat1DL* | 1yp2 | 51.08 | 0.74 | 0.715 | -599.19 |
| Wheat 7DS** | 1yp2 | 92.43 | 0.77 | 0.709 | -2758.52 |
| *T. urartu* | 1yp2 | 48.96 | 0.64 | 0.663 | -434.08 |
|  | 1yp2 | 92.46 | 0.69 | 0.677 | -2494.61 |
| *Ae. tauschii* | 1yp2 | 50.72 | 0.66 | 0.706 | -607.09 |
|  | 1yp2 | 92.49 | 0.98 | 0.728 | -2469.45 |
| *Brachypodium* | 1yp2 | 51.64 | 0.67 | 0.659 | -581.28 |
|  | 1yp2 | 91.80 | 0.91 | 0.681 | -2626.92 |
| Rice | 1yp2 | 52.34 | 0.69 | 0.700 | -587.39 |
|  | 1yp2 | 91.78 | 0.79 | 0.726 | -2655.60 |
| Barley | 1yp2 | 51.64 | 0.68 | 0.663 | -577.84 |
|  | 1yp2 | 90.58 | 0.92 | 0.718 | -2636.74 |
| Sorghum | 1yp2 | 52.11 | 0.67 | 0.674 | -585.95 |
|  | 1yp2 | 87.08 | 0.71 | 0.743 | -631.87 |
| *Arabidopsis* | 1yp2 | 57.08 | 0.71 | 0.682 | -606.37 |
|  | 1yp2 | 94.44 | 0.79 | 0.718 | -2663.52 |
| Chickpea | 1yp2 | 53.26 | 0.68 | 0.647 | -572.99 |
|  | 1yp2 | 94.00 | 0.78 | 0.705 | -2672.34 |
| Potato | 1yp2 | 53.49 | 0.68 | 0.674 | -585.95 |
|  | 1yp2 | 99.11 | 0.80 | 0.721 | -2682.63 |

* indicates wheat homoeologues of group 1 chromosomes, ** indicates wheat homoeologues of group 7 chromosomes
