# Supplementary material for: Comparative Analysis of AGPase Genes and Encoded Proteins in Eight Monocots and Three Dicots with Emphasis on Wheat
Source: Front Plant Sci. 2017 Jan 24;8:19. doi: 10.3389/fpls.2017.00019 (PMC5259687; doi:10.3389/fpls.2017.00019)
Supplement: Supplementary file 12 [file Table12.DOCX]

**Supplementary material**

**Comparative analysis of AGPase genes and encoded proteins in eight monocots and three dicots with emphasis on wheat**

Ritu Batra^1¶,^ Gautam Saripalli^1¶^, Amita Mohan^2^, Kulvinder S. Gill^2*^, Harindra Singh Balyan^1^ and Pushpendra Kumar Gupta^1^

*Correspondence:

Kulvinder S. Gill

email: [ksgill@wsu.edu](mailto:ksgill@wsu.edu)

Phone: 509-335-4666

**Supplementary Table 12**: Model quality assessment scores of 3D structures of AGPase LS (upper row) and AGPase SS (lower row) in 11 species including the three homoeologues on group 1 (LS) and 7 (SS) chromosome of wheat.

| Species | PROCHEK | | | | | ERRAT | VERIFY3D | Status of 3D structure from SAVES |
| --- | --- | --- | --- | --- | --- | --- | --- | --- |
|  | Favoured*** region (%) | Allowed region  (%) | Generously allowed region (%) | Disallowed region  (%) | G-factor | Quality factor  (%) | (3D-1D Profile)  (%) |  |
| Maize | 92.6 | 5.7 | 0.7 | 0.6 | 0.02 | 77.16 | 96.34 | Pass |
|  | 90.4 | 8.7 | 0.5 | 0.5 | -0.05 | 82.78 | 99.32 | Pass |
| Wheat 1AL* | 88.1 | 11.1 | 0.5 | 0.3 | -0.08 | 88.59 | 99.53 | Pass |
| Wheat 7AS** | 88.1 | 11.0 | 0.5 | 0.4 | -0.18 | 93.45 | 95.24 | Pass |
| Wheat 1BL* | 88.3 | 10.8 | 0.6 | 0.3 | -0.08 | 88.56 | 99.74 | Pass |
| Wheat 7BS** | 88.1 | 11.0 | 0.5 | 0.4 | -0.18 | 93.45 | 95.24 | Pass |
| Wheat 1DL* | 88.1 | 11.1 | 0.5 | 0.3 | -0.08 | 88.59 | 99.53 | Pass |
| Wheat 7DS** | 88.9 | 10.3 | 0.4 | 0.5 | -0.16 | 93.74 | 99.32 | Pass |
| *T. urartu* | 86.0 | 13.7 | 0.3 | 0.0 | -0.15 | 90.19 | 92.40 | Pass |
|  | 89.6 | 9.2 | 0.8 | 0.4 | -0.18 | 93.11 | 99.50 | Pass |
| *Ae. tauschii* | 88.2 | 11.1 | 0.5 | 0.3 | -0.09 | 88.48 | 97.68 | Pass |
|  | 87.7 | 11.6 | 0.3 | 0.4 | -0.16 | 94.99 | 99.52 | Pass |
| *Brachypodium* | 90.3 | 7.4 | 1.6 | 0.7 | -0.02 | 74.23 | 91.22 | Pass |
|  | 89.1 | 10.0 | 0.5 | 0.5 | -0.09 | 81.50 | 98.86 | Pass |
| Rice | 91.0 | 6.9 | 1.6 | 0.5 | -0.02 | 73.88 | 90.85 | Pass |
|  | 90.1 | 9.4 | 0.2 | 0.3 | -0.06 | 82.43 | 99.32 | Pass |
| Barley | 90.3 | 7.2 | 1.6 | 1.0 | -0.02 | 72.10 | 93.76 | Pass |
|  | 90.6 | 8.7 | 0.3 | 0.3 | -0.1 | 80.46 | 99.09 | Pass |
| Sorghum | 88.7 | 7.9 | 3.0 | 0.5 | -0.02 | 75.95 | 93.79 | Pass |
|  | 82.2 | 15.7 | 1.6 | 0.5 | -0.06 | 87.01 | 93.18 | Pass |
| *Arabidopsis* | 94.0 | 5.5 | 0.3 | 0.2 | 0.00 | 77.88 | 95.64 | Pass |
|  | 90.5 | 8.6 | 0.7 | 0.3 | -0.05 | 83.53 | 99.55 | Pass |
| Chickpea | 87.1 | 9.7 | 2.1 | 1.1 | -0.09 | 74.52 | 96.55 | Pass |
|  | 90.4 | 8.9 | 0.4 | 0.3 | -0.06 | 80.00 | 99.55 | Pass |
| Potato | 88.7 | 7.9 | 2.3 | 1.2 | -0.02 | 75.95 | 93.79 | Pass |
|  | 90.2 | 8.9 | 0.7 | 0.3 | -0.06 | 81.85 | 99.55 | Pass |

* indicates wheat homoeologues of group 1 chromosomes, ** indicates wheat homoeologues of group 7 chromosomes, *** Based on an analysis of 118 structures of resolution of at least 2.0 A^o^ and R-factor no greater than 20%, a good quality model would be expected to have maximum number of amino acids in the most favoured regions.
