# Supplementary material for: Comparative Analysis of AGPase Genes and Encoded Proteins in Eight Monocots and Three Dicots with Emphasis on Wheat
Source: Front Plant Sci. 2017 Jan 24;8:19. doi: 10.3389/fpls.2017.00019 (PMC5259687; doi:10.3389/fpls.2017.00019)
Supplement: Supplementary file 13 [file Table13.DOCX]

**Supplementary material**

**Comparative analysis of AGPase genes and encoded proteins in eight monocots and three dicots with emphasis on wheat**

Ritu Batra^1¶,^ Gautam Saripalli^1¶^, Amita Mohan^2^, Kulvinder S. Gill^2*^, Harindra Singh Balyan^1^ and Pushpendra Kumar Gupta^1^

*Correspondence:

Kulvinder S. Gill

email: [ksgill@wsu.edu](mailto:ksgill@wsu.edu)

Phone: 509-335-4666

**Supplementary Table 13**: Predicted values of different parameters of superimposition of 3D protein structures of AGPase LS and AGPase SS of different plant species over 3D protein structure of maize AGPaseLS

| Species | RMSD* | | % identity | | % similarity | |
| --- | --- | --- | --- | --- | --- | --- |
|  | AGPase  LS | AGPase  SS | AGPase  LS | AGPase SS | AGPase LS | AGPase SS |
| Wheat 1AL**/7AS*** | 1.07 | 1.31 | 76.5 | 99.8 | 90.8 | 100.0 |
| Wheat 1BL**/7BS*** | 1.12 | 1.31 | 77.8 | 99.8 | 90.5 | 100.0 |
| Wheat 1DL**/7DS*** | 1.07 | 0.37 | 76.3 | 98.6 | 90.5 | 99.1 |
| *T. urartu* | 2.25 | 1.80 | 63.3 | 98.3 | 74.0 | 99.0 |
| *Ae. tauschii* | 1.07 | 1.71 | 74.5 | 100.0 | 88.4 | 100.0 |
| *Brachypodium* | 2.01 | 0.50 | 76.4 | 98.9 | 90.8 | 99.5 |
| Rice | 1.63 | 0.19 | 75.1 | 97.0 | 88.9 | 98.0 |
| Barley | 1.57 | 1.42 | 64.7 | 88.0 | 79.8 | 95.7 |
| Sorghum | 1.64 | 1.50 | 74.4 | 88.0 | 88.7 | 95.7 |
| *Arabidopsis* | 0.81 | 0.39 | 62.7 | 91.2 | 80.3 | 96.0 |
| Chickpea | 1.51 | 0.33 | 62.4 | 90.3 | 78.2 | 96.2 |
| Potato | 1.57 | 0.85 | 64.7 | 91.2 | 79.8 | 96.2 |

*Root Mean Square Deviation; **indicates wheat homoeologues of group 1 chromosomes of AGPase LS, *** indicates wheat homoeologues of group 7 chromosomes of AGPase SS
