# Supplementary material for: Comparative Analysis of AGPase Genes and Encoded Proteins in Eight Monocots and Three Dicots with Emphasis on Wheat
Source: Front Plant Sci. 2017 Jan 24;8:19. doi: 10.3389/fpls.2017.00019 (PMC5259687; doi:10.3389/fpls.2017.00019)
Supplement: Supplementary file 14 [file Table14.DOCX]

**Supplementary material**

**Comparative analysis of AGPase genes and encoded proteins in eight monocots and three dicots with emphasis on wheat**

Ritu Batra^1¶,^ Gautam Saripalli^1¶^, Amita Mohan^2^, Kulvinder S. Gill^2*^, Harindra Singh Balyan^1^ and Pushpendra Kumar Gupta^1^

*Correspondence:

Kulvinder S. Gill

email: [ksgill@wsu.edu](mailto:ksgill@wsu.edu)

Phone: 509-335-4666

| Species | Amino acids and their position | | | | | | | | | | | | | | | | |
| --- | --- | --- | --- | --- | --- | --- | --- | --- | --- | --- | --- | --- | --- | --- | --- | --- | --- |
| Maize | LEU | GLY | GLY | GLY | GLN | MET | GLN | GLY | THR | SER | SER | GLY | ASP |  |  |  |  |
|  | 89 | 90 | 91 | 92 | 96 | 136 | 181 | 182 | 183 | 186 | 210 | 211 | 212 |  |  |  |  |
| Wheat 1AL | LEU | GLY | GLY | GLY | GLN | MET | ARG | GLY | THR | ALA | SER | GLY | ASP | GLY | SER |  |  |
|  | 35 | 36 | 37 | 38 | 42 | 82 | 127 | 128 | 129 | 132 | 156 | 157 | 158 | 268 | 269 |  |  |
| Wheat 1BL | LEU | GLY | GLY | GLY | GLN | MET | ARG | GLY | THR | ALA | SER | GLY | ASP | GLY | SER |  |  |
|  | 45 | 46 | 47 | 48 | 52 | 92 | 137 | 138 | 139 | 142 | 166 | 167 | 168 | 278 | 279 |  |  |
| Wheat 1DL | LEU | GLY | GLY | GLY | MET | PHE | ARG | GLY | THR | ALA | SER | GLY | ASP | ASP |  |  |  |
|  | 35 | 36 | 37 | 38 | 82 | 126 | 127 | 128 | 129 | 132 | 156 | 157 | 158 | 266 |  |  |  |
| *T. urartu* | PHE | SER | GLY | ASP |  |  |  |  |  |  |  |  |  |  |  |  |  |
|  | 75 | 105 | 106 | 107 |  |  |  |  |  |  |  |  |  |  |  |  |  |
| *Ae. tauschii* | LEU | GLY | GLY | GLY | GLN | MET | ARG | GLY | THR | ALA | SER | GLY | ASP | GLY | SER |  |  |
|  | 32 | 33 | 34 | 35 | 39 | 79 | 124 | 125 | 126 | 129 | 153 | 154 | 155 | 265 | 266 |  |  |
| *Brachypodium* | LEU | GLY | GLY | GLY | GLN | MET | PHE | ARG | GLY | THR | ALA | SER | GLY | ASP | GLU | LYS | GLY |
|  | 96 | 96 | 97 | 98 | 102 | 142 | 186 | 187 | 188 | 189 | 192 | 216 | 217 | 218 | 270 | 271 | 328 |
| Rice | LEU | GLY | GLY | GLY | GLN | MET | PHE | GLN | GLY | THR | ALA | CYS | GLY | ASP | GLU | LYS | GLY |
|  | 91 | 92 | 93 | 94 | 98 | 138 | 182 | 183 | 184 | 185 | 188 | 212 | 213 | 214 | 266 | 267 | 324 |
| Barley | LEU | GLY | GLY | GLY | GLN | MET | PHE | ARG | GLY | THR | ALA | SER | GLY | ASP | GLU | LYS | GLY |
|  | 96 | 97 | 98 | 99 | 103 | 143 | 187 | 188 | 189 | 190 | 193 | 217 | 218 | 219 | 271 | 272 | 329 |
| Sorghum | LEU | GLY | GLY | GLY | GLU | GLY | GLN | GLY | THR | ALA | SER | GLY | ASP | GLY |  |  |  |
|  | 100 | 101 | 102 | 103 | 104 | 105 | 193 | 194 | 195 | 198 | 221 | 222 | 331 | 333 |  |  |  |
| *Arabidopsis* | LEU | GLY | GLY | GLY | ARG | LYS | LEU | GLN | GLY | THR | ALA | SER | GLY | ASP |  |  |  |
|  | 90 | 91 | 92 | 93 | 97 | 107 | 137 | 184 | 185 | 186 | 189 | 212 | 213 | 214 |  |  |  |
| Chickpea | GLY | GLY | GLY | PRO | GLY | GLN | GLY | THR | ALA | ALA | GLY | ASP | GLY |  |  |  |  |
|  | 92 | 93 | 94 | 95 | 96 | 184 | 185 | 186 | 189 | 212 | 213 | 322 | 324 |  |  |  |  |
| Potato | LEU | GLY | GLY | GLY | GLU | GLY | GLN | GLY | THR | ALA | SER | GLY | ASP | GLY |  |  |  |
|  | 100 | 101 | 102 | 103 | 104 | 105 | 193 | 194 | 195 | 198 | 221 | 222 | 331 | 333 |  |  |  |

**Supplementary Table 14:** Ligand binding amino acid residues and their position in AGPase LS of 11 species (including the three wheat homoeologues on group 1 chromosomes
