# Supplementary material for: Comparative Analysis of AGPase Genes and Encoded Proteins in Eight Monocots and Three Dicots with Emphasis on Wheat
Source: Front Plant Sci. 2017 Jan 24;8:19. doi: 10.3389/fpls.2017.00019 (PMC5259687; doi:10.3389/fpls.2017.00019)
Supplement: Supplementary file 15 [file Presentation1.ZIP › Supplementary Figures/Supplementary Figures/~WRL2827.tmp]

**Supplementary Figure Legends:**

**Supplementary Figure 1**: Microsynteny analysis of AGPase SS using Genomicus tool.

**Supplementary Figure 2**: Microsynteny analysis of AGPase LS using Genomicus tool.

**Supplementary Figure 3:** Regulatory elements identified in 1kb upstream region of AGPase SS. Different symbols indicate major regulatory elements identified. TATA box (
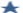
), CAAT box (
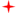
), light responsive response elements (
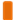
), abiotic stresses responsive elements (
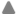
), endosperm expression responsive elements (
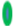
).

**Supplementary Figure 4**: Multiple sequence alignment of AGPase LS

**Supplementary Figure 5**: Multiple sequence alignment of AGPase SS

**Supplementary Figure 6**: The root mean square deviation (RMSD) of the Cα atoms and backbone of the teramer structures as a function of time.

**Supplementary Figure 7A**: The B-Factor profiles and RMSF of the Cα atoms and backbone of (a) Wheat,(b) Sorghum, (c) Barley, (d) Potato, (e) *Arabidopsis* and (f) Maize.

**Supplementary Figure 7B**: The B-Factor profiles and RMSF of the Cα atoms and backbone of (a) *T. urartu*, (b) *Ae. tauschii*, (c) *Brachypodium*, (d) rice, and (e) chickpea

**Supplementary Figure 8**: Superimposed structure of the predicted maize AGPase heterotetramer (green coloured) over Potato AGPase homotetramer (cyan coloured).

**Supplementary Figure 9**: Superimposed structure of the predicted *T. urartu* AGPase heterotetramer (gray coloured) over Potato AGPase homotetramer (cyan coloured).

**Supplementary Figure 10**: Superimposed structure of the predicted *Ae. tauschii* AGPase heterotetramer (purple coloured) over Potato AGPase homotetramer (cyan coloured).

**Supplementary Figure 11**: Superimposed structure of the predicted *Brachypodium* AGPase heterotetramer (red coloured) over Potato AGPase homotetramer (cyan coloured).

**Supplementary Figure 12**: Superimposed structure of the predicted rice AGPase heterotetramer (pink coloured) over Potato AGPase homotetramer (cyan coloured).

**Supplementary Figure 13**: Superimposed structure of the predicted barley AGPase heterotetramer (red coloured) over Potato AGPase homotetramer (cyan coloured).

**Supplementary Figure 14**: Superimposed structure of the predicted sorghum AGPase heterotetramer (blue coloured) over Potato AGPase homotetramer (cyan coloured).

**Supplementary Figure 15**: Superimposed structure of the predicted *Arabidopsis* AGPase heterotetramer (gray coloured) over Potato AGPase homotetramer (cyan coloured).

**Supplementary Figure 16**: Superimposed structure of the predicted chickpea AGPase heterotetramer (yellow coloured) over Potato AGPase homotetramer (cyan coloured).

**Supplementary Figure 17**: Superimposed structure of the predicted potato AGPase heterotetramer (pink coloured) over Potato AGPase homotetramer (cyan coloured).
