# Supplementary figures and images for: Comparative Analysis of AGPase Genes and Encoded Proteins in Eight Monocots and Three Dicots with Emphasis on Wheat
Source: Front Plant Sci. 2017 Jan 24;8:19. doi: 10.3389/fpls.2017.00019 (PMC5259687; doi:10.3389/fpls.2017.00019)

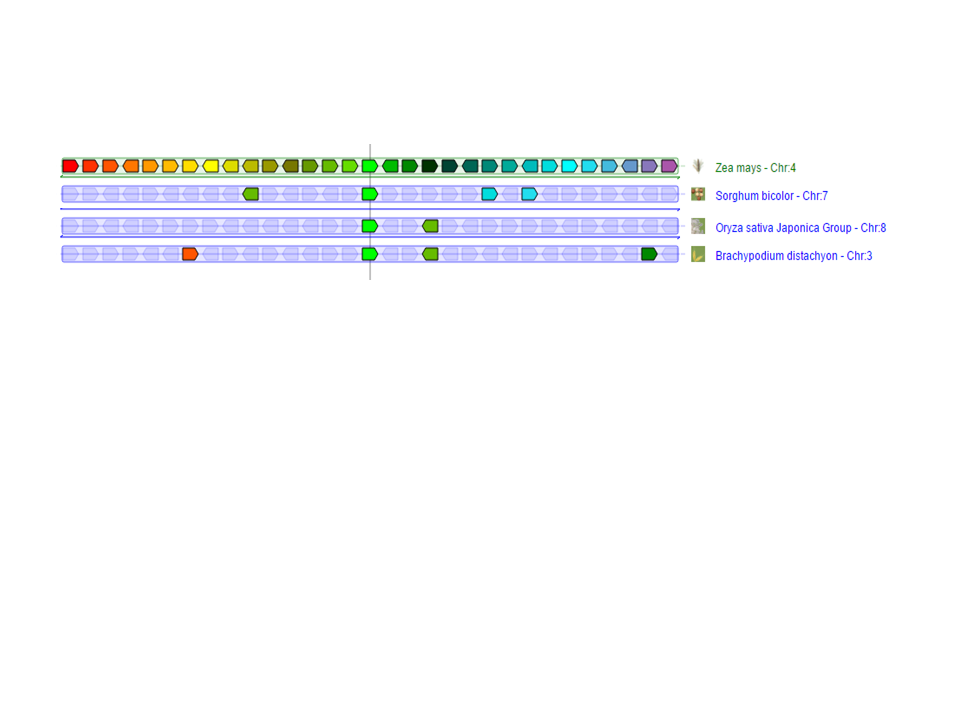

Supplement: Supplementary file 15 [file Presentation1.ZIP › Supplementary Figures/Supplementary Figures/Supplementary Figure 1.tif]

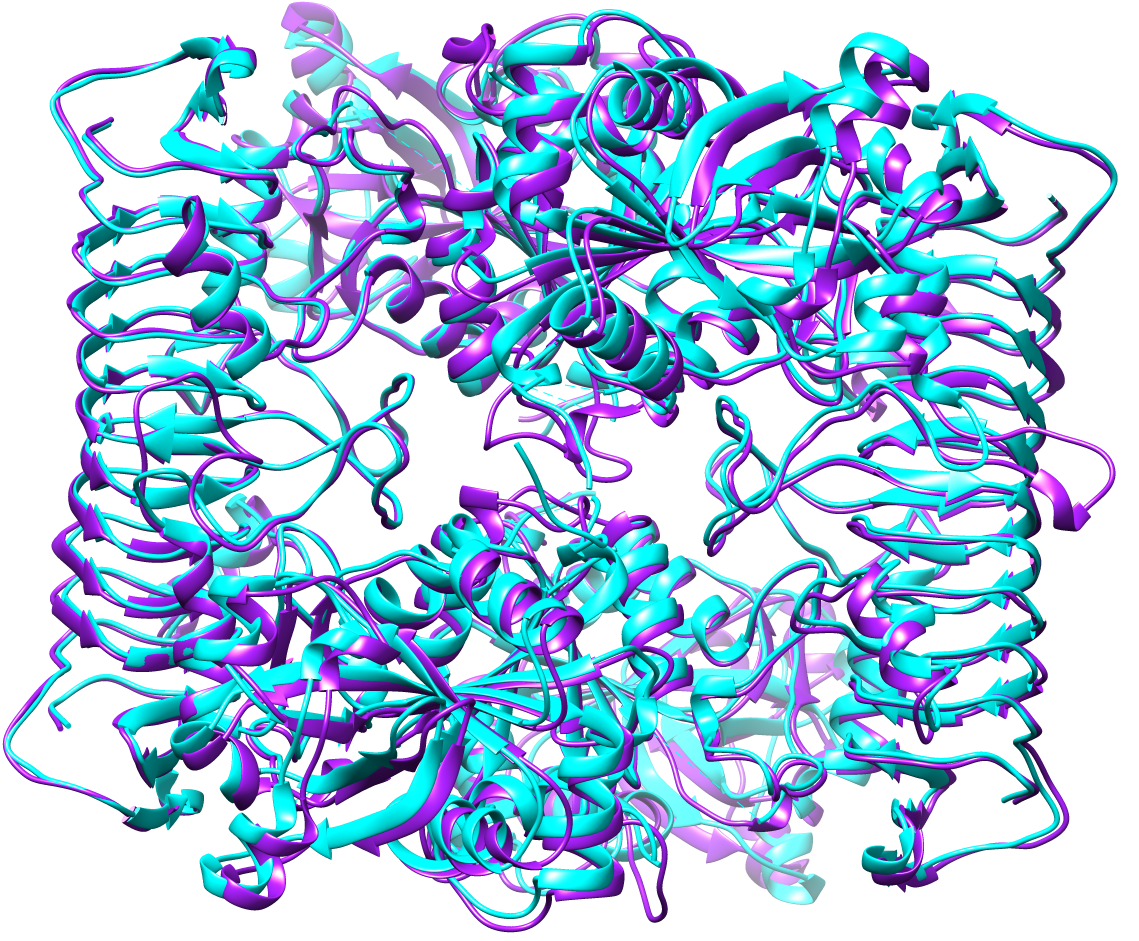

Supplement: Supplementary file 15 [file Presentation1.ZIP › Supplementary Figures/Supplementary Figures/Supplementary Figure 10.tif]

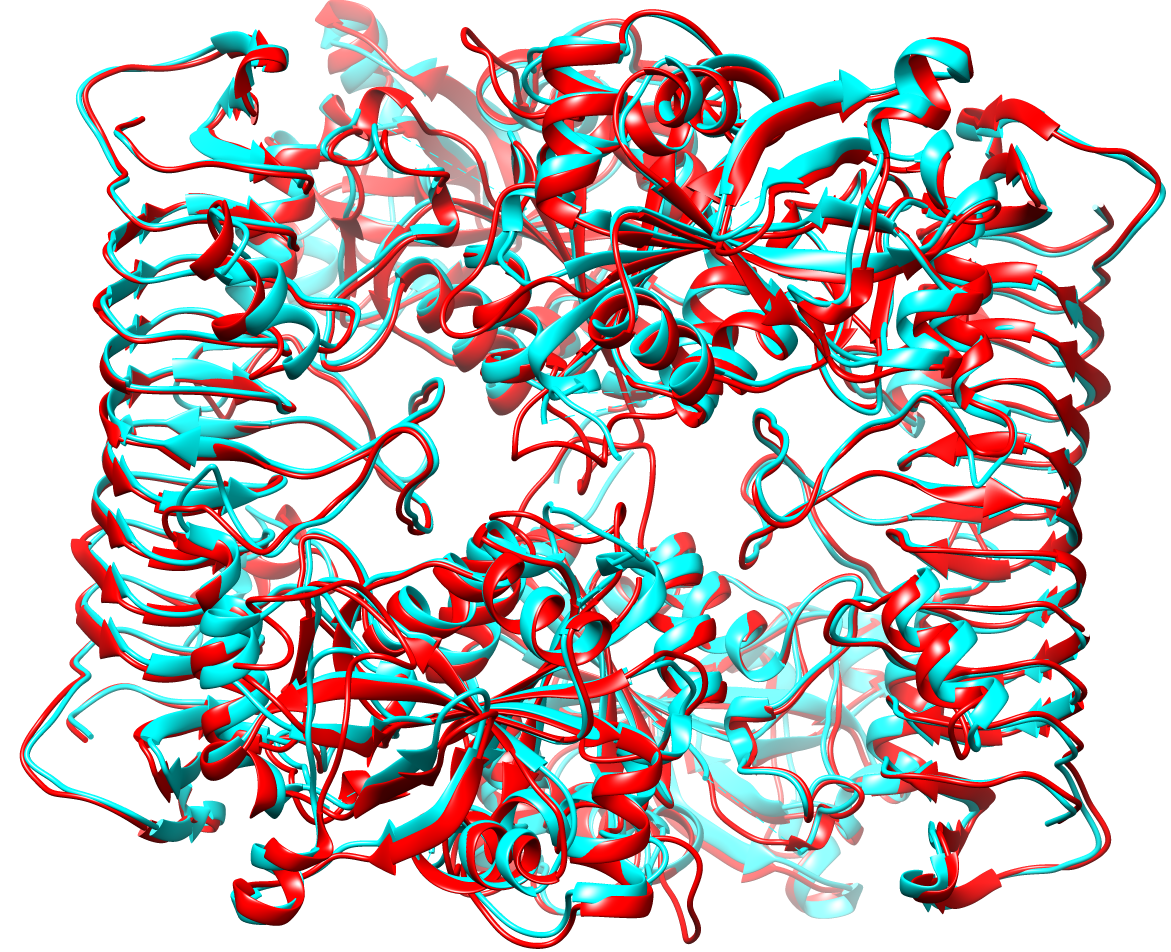

Supplement: Supplementary file 15 [file Presentation1.ZIP › Supplementary Figures/Supplementary Figures/Supplementary Figure 11 .tif]

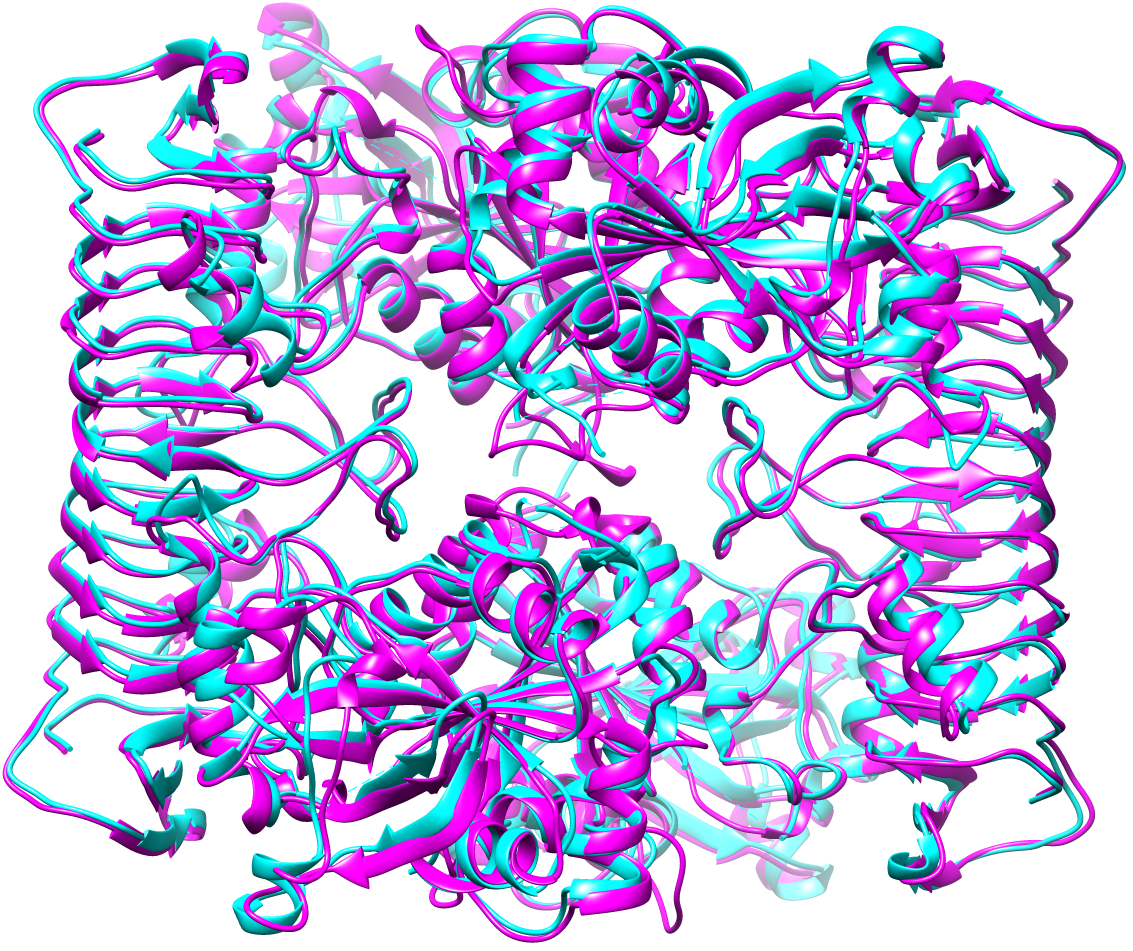

Supplement: Supplementary file 15 [file Presentation1.ZIP › Supplementary Figures/Supplementary Figures/Supplementary Figure 12.tif]

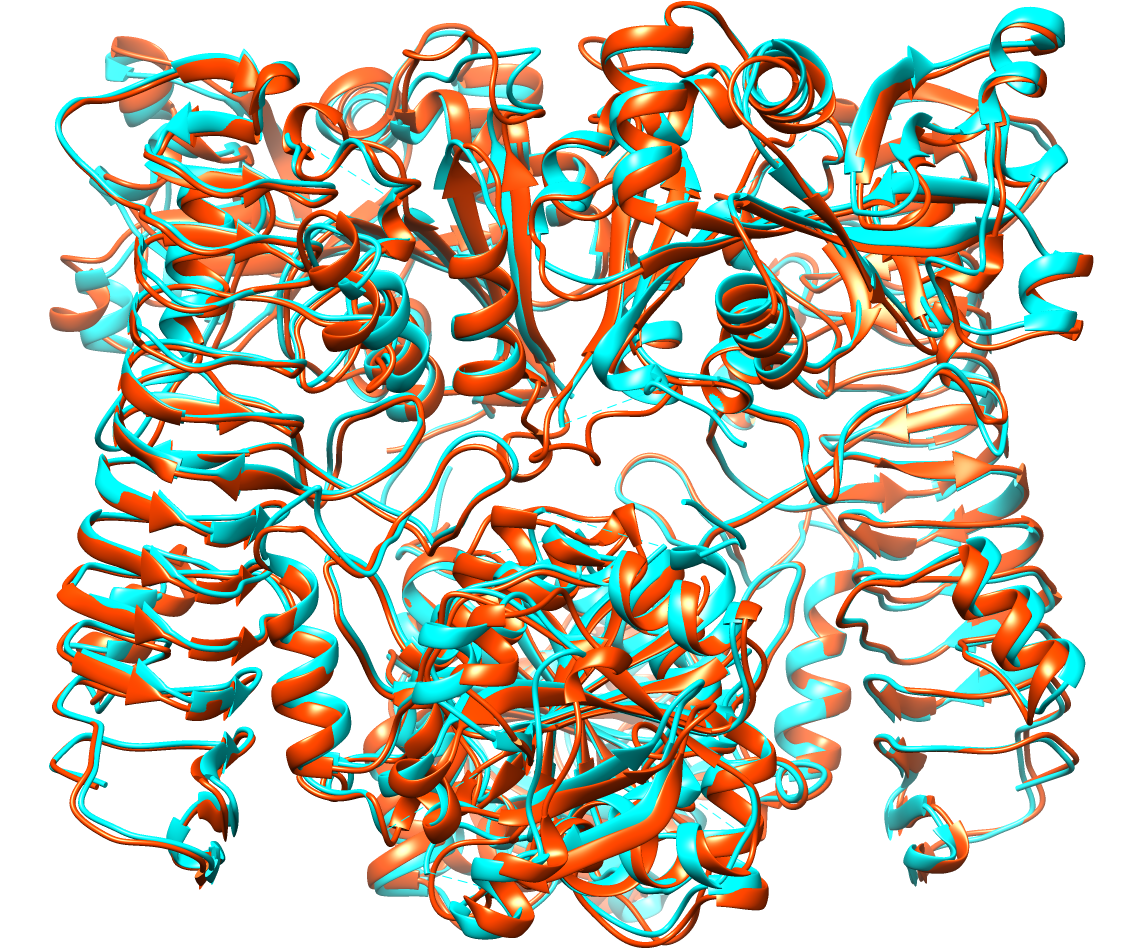

Supplement: Supplementary file 15 [file Presentation1.ZIP › Supplementary Figures/Supplementary Figures/Supplementary Figure 13.tif]

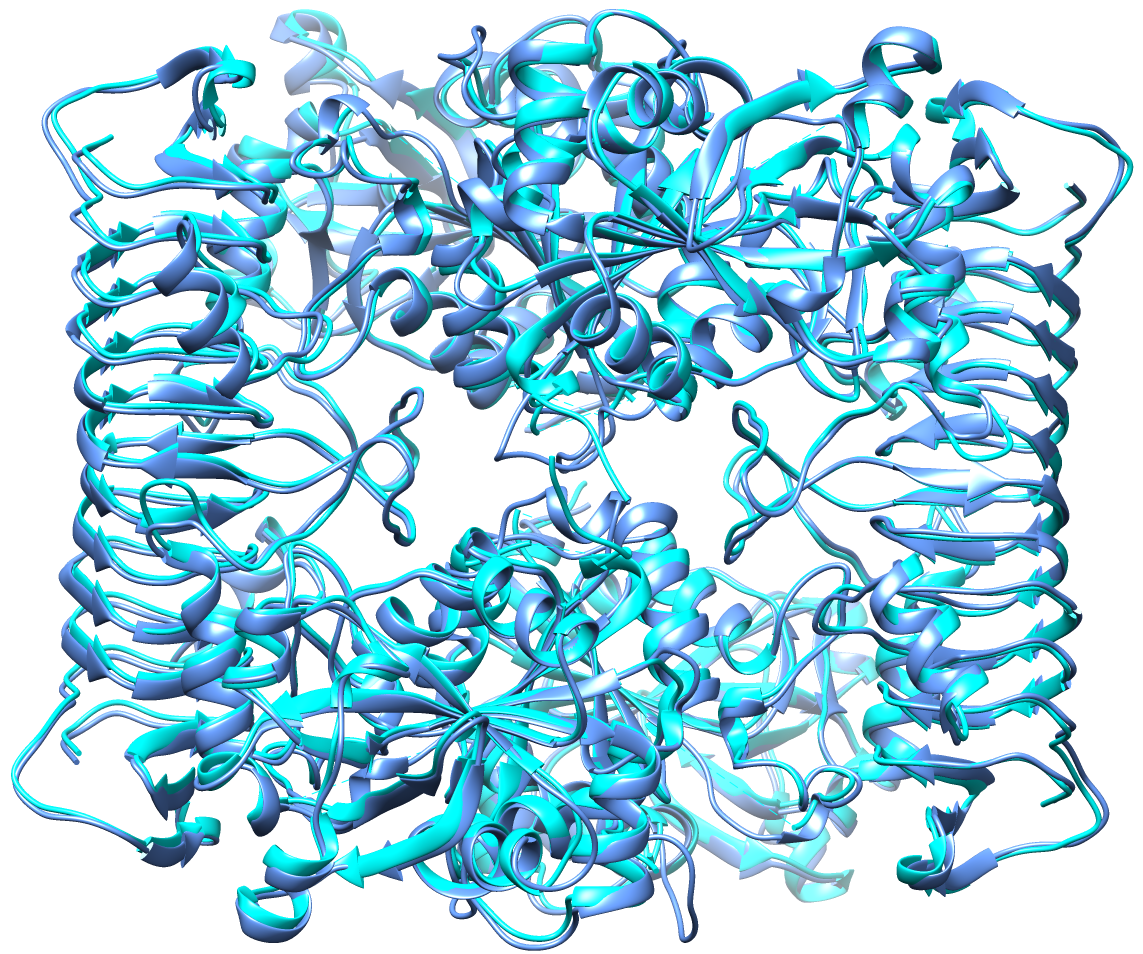

Supplement: Supplementary file 15 [file Presentation1.ZIP › Supplementary Figures/Supplementary Figures/Supplementary Figure 14.tif]

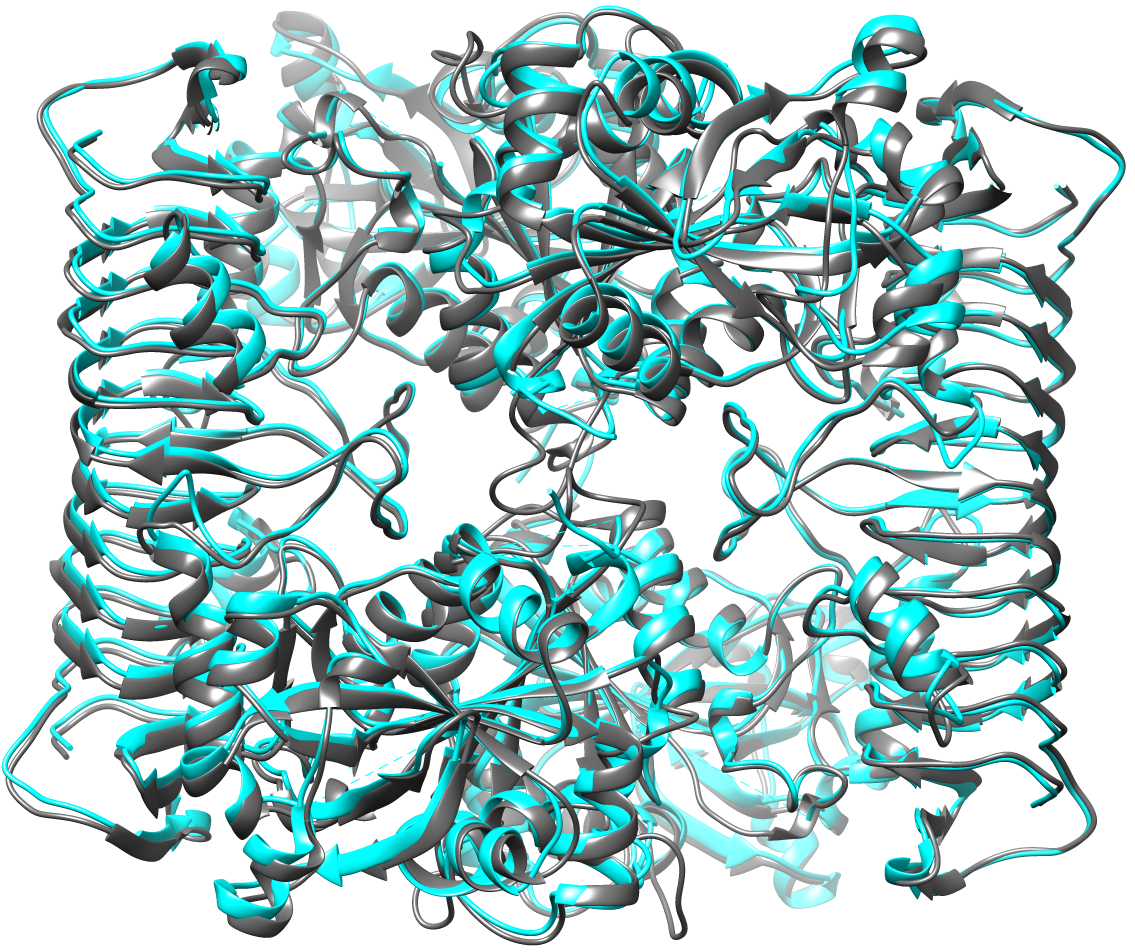

Supplement: Supplementary file 15 [file Presentation1.ZIP › Supplementary Figures/Supplementary Figures/Supplementary Figure 15.tif]

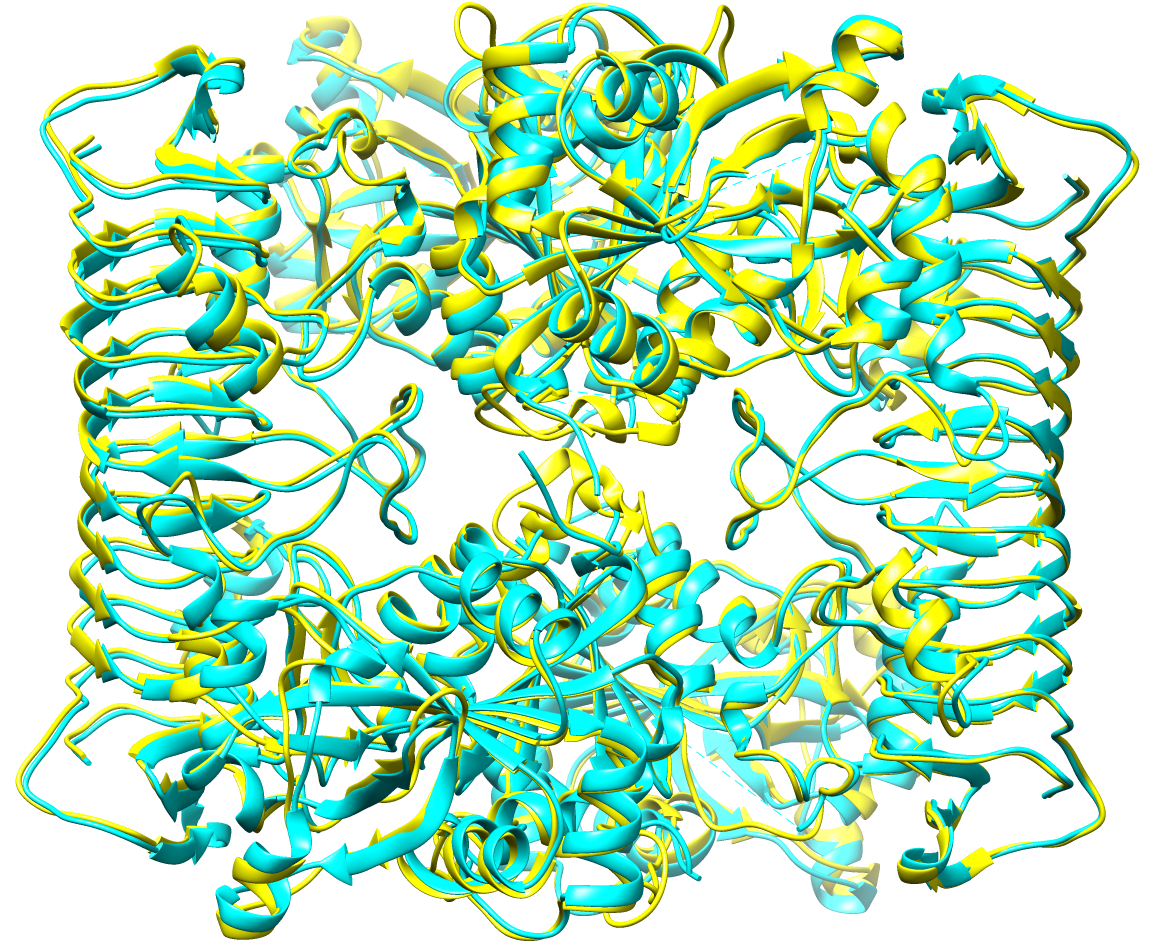

Supplement: Supplementary file 15 [file Presentation1.ZIP › Supplementary Figures/Supplementary Figures/Supplementary Figure 16.tif]

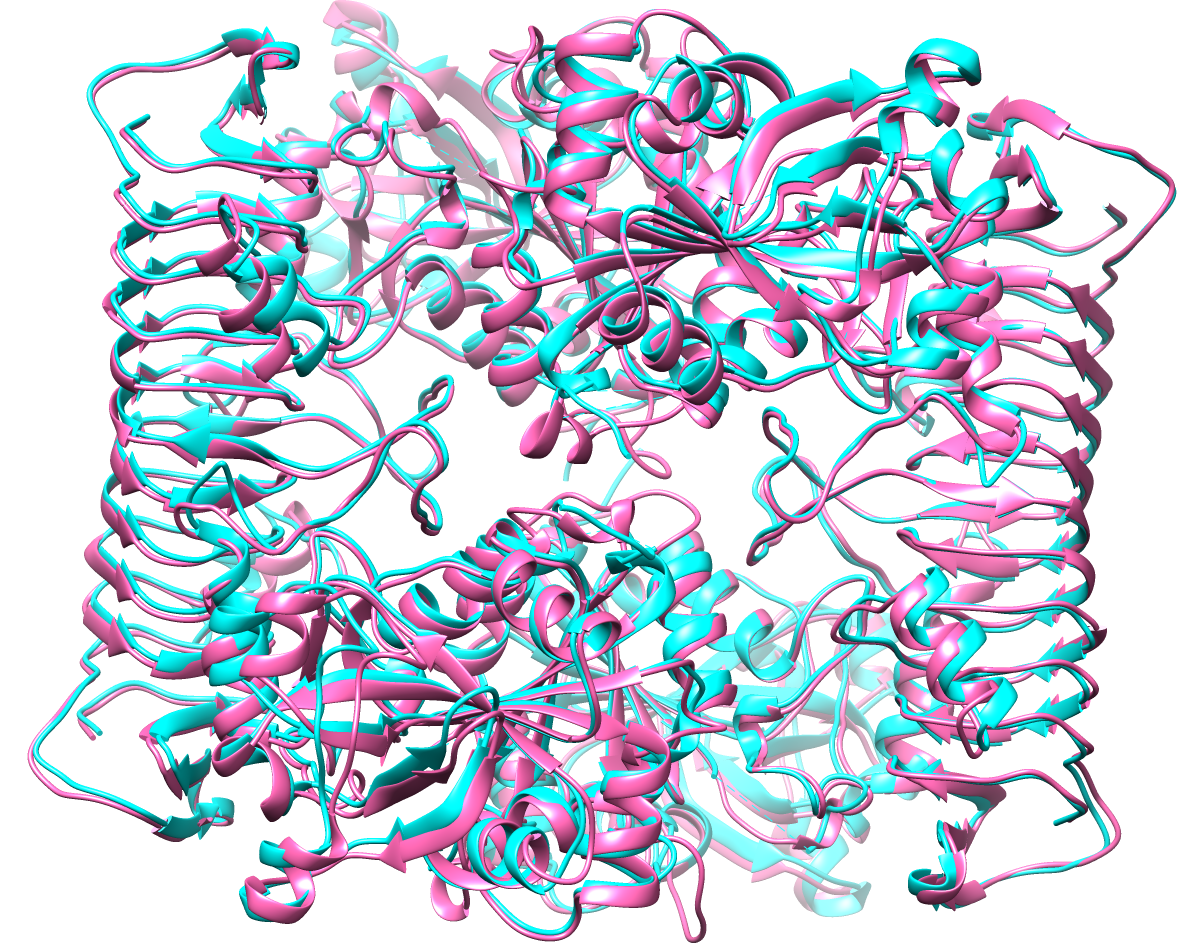

Supplement: Supplementary file 15 [file Presentation1.ZIP › Supplementary Figures/Supplementary Figures/Supplementary Figure 17.tif]

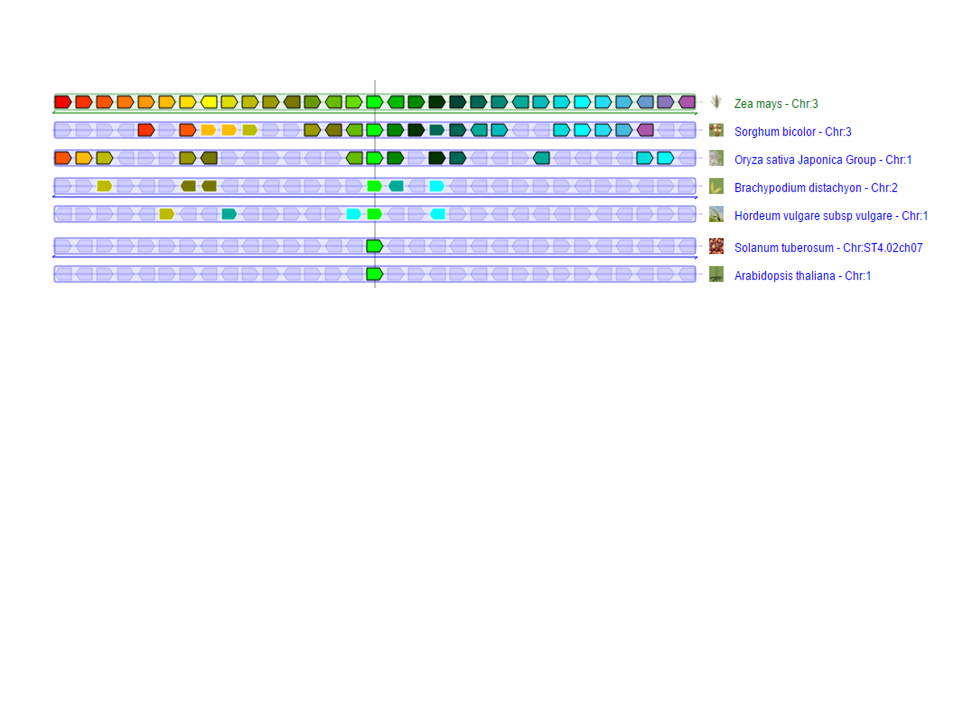

Supplement: Supplementary file 15 [file Presentation1.ZIP › Supplementary Figures/Supplementary Figures/Supplementary Figure 2.tif]

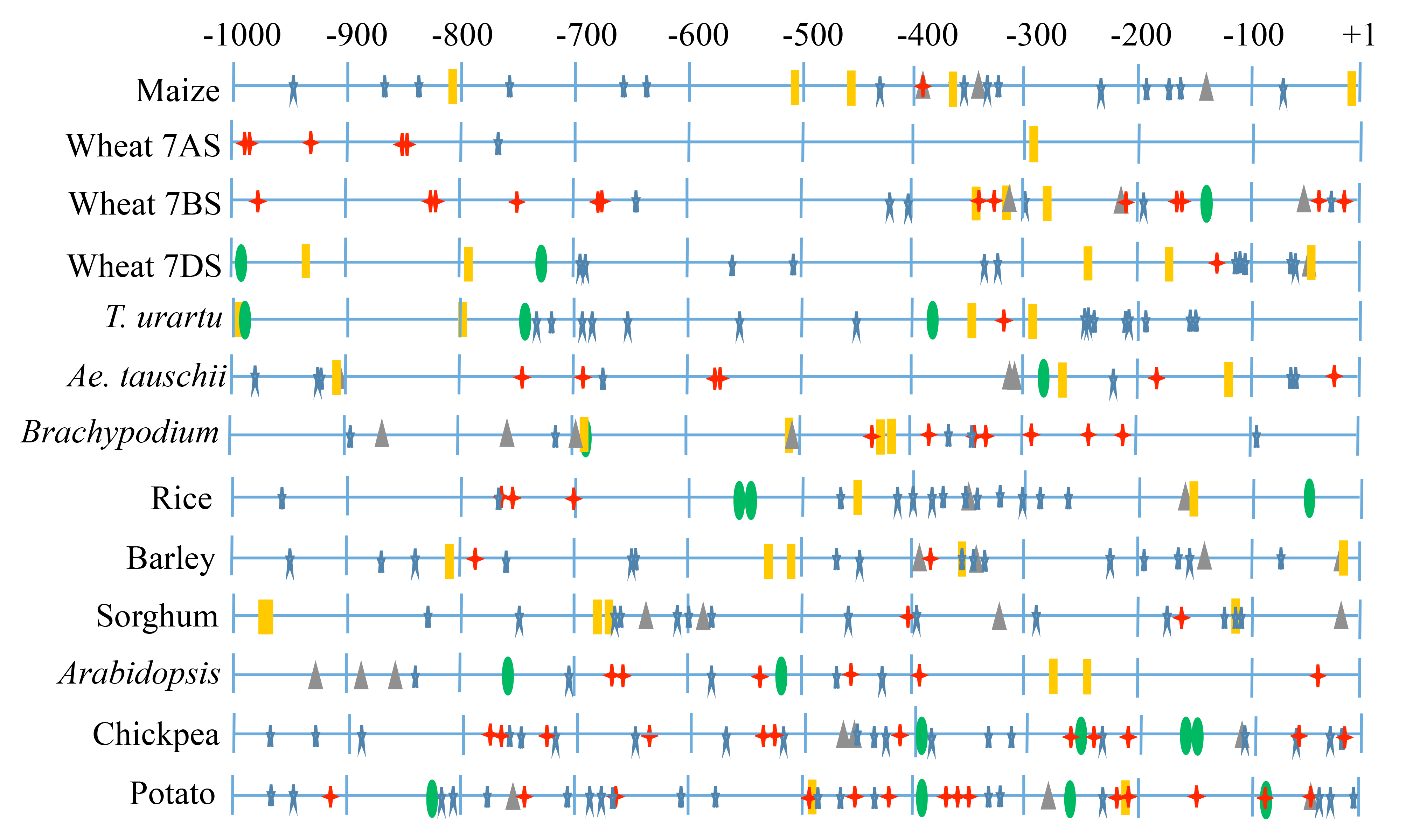

Supplement: Supplementary file 15 [file Presentation1.ZIP › Supplementary Figures/Supplementary Figures/Supplementary Figure 3.tif]

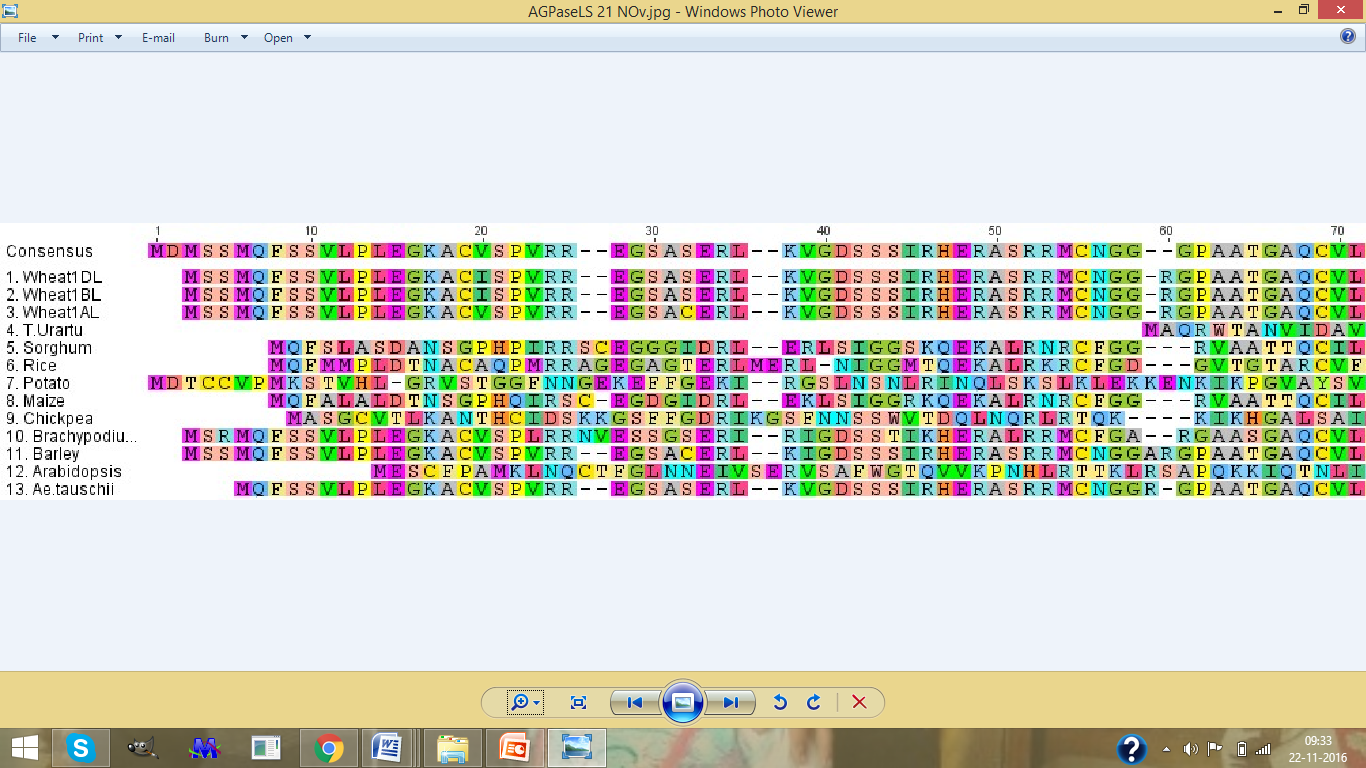


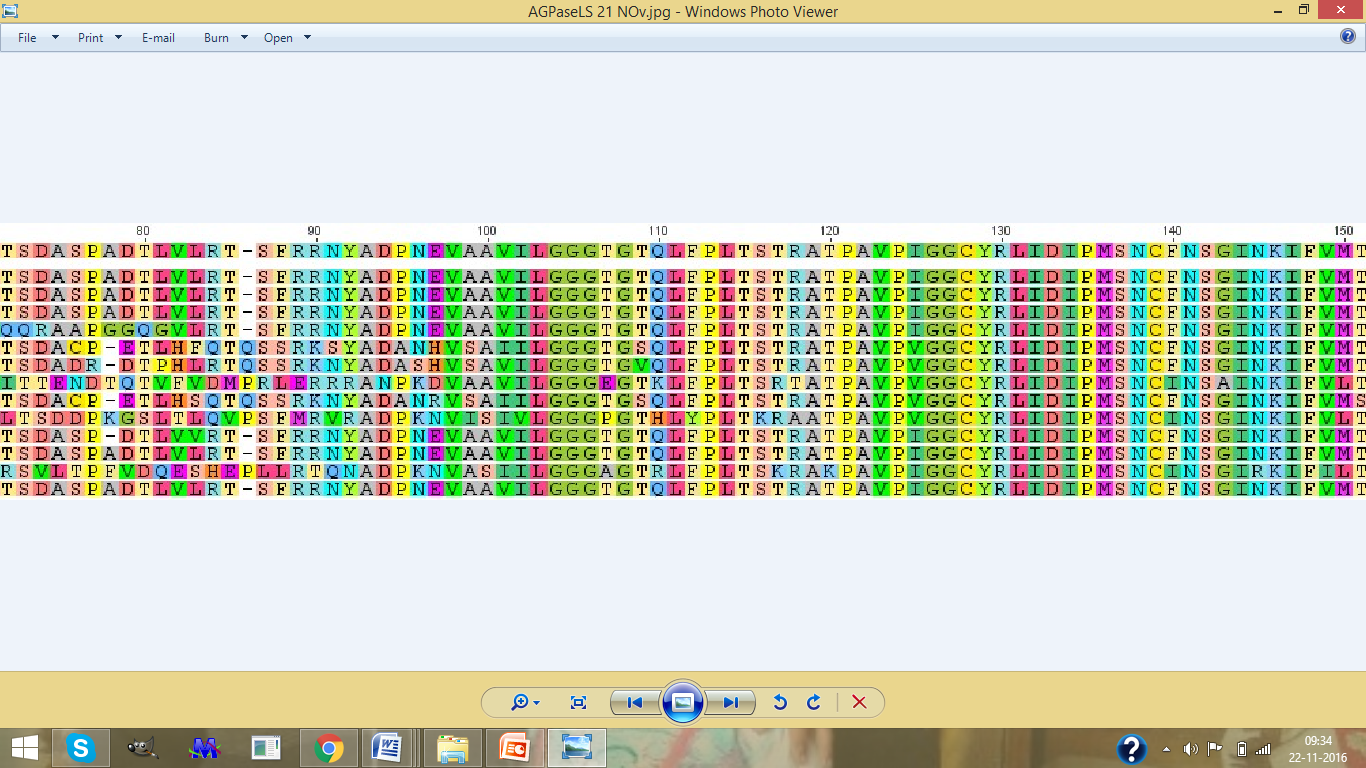


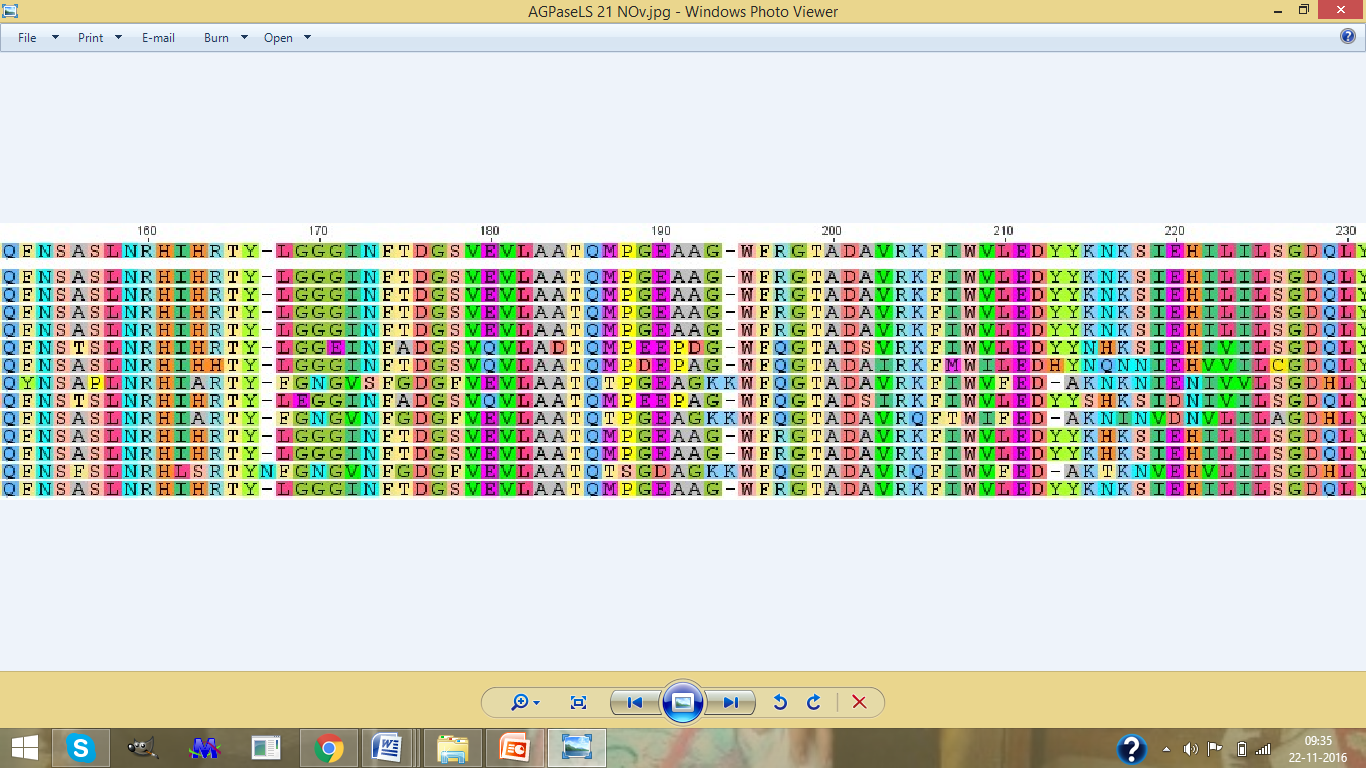


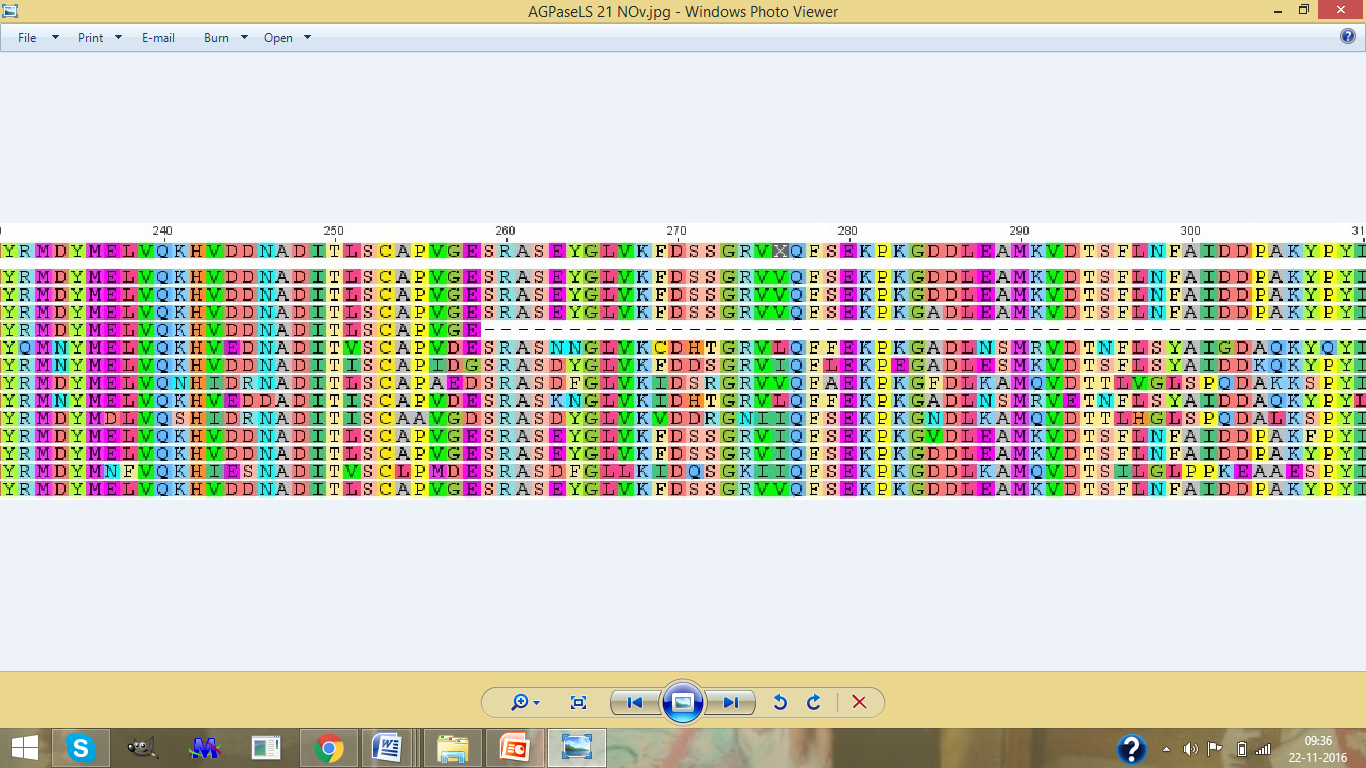


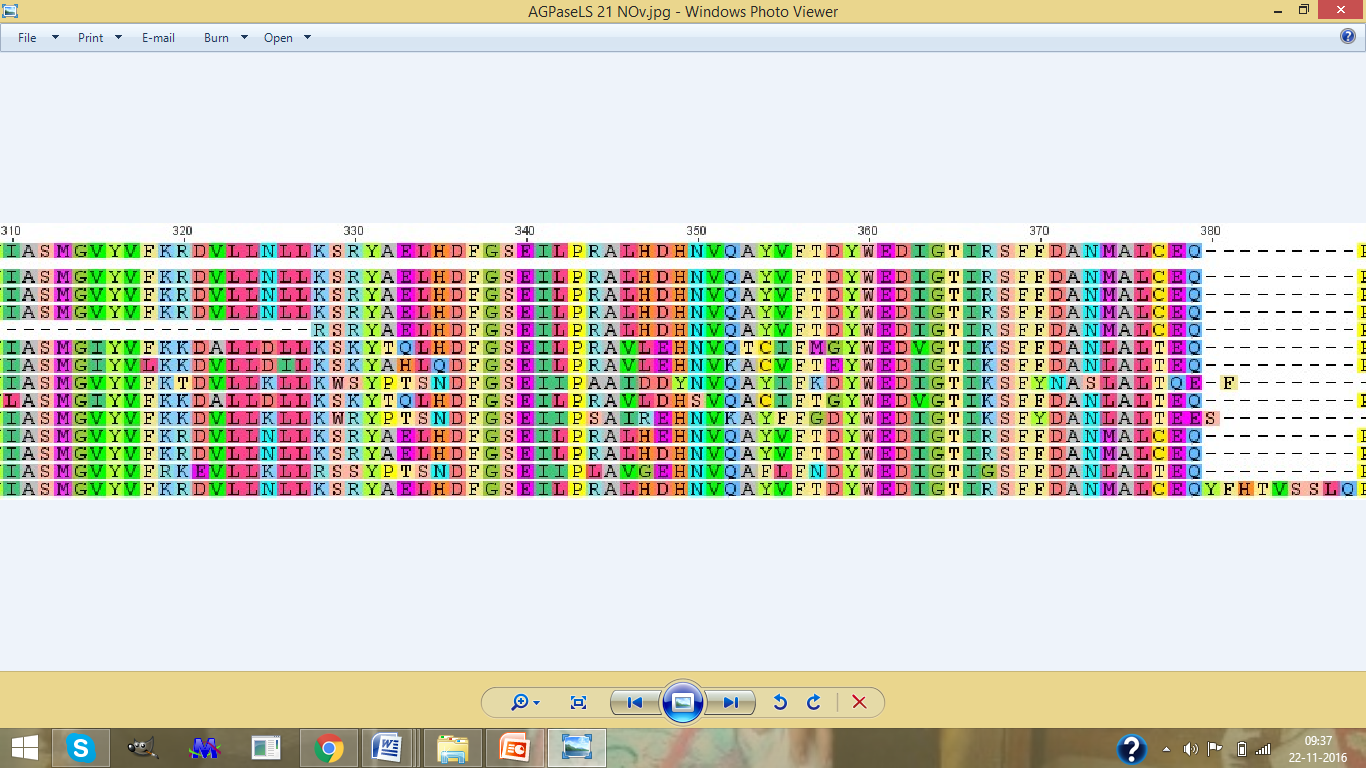


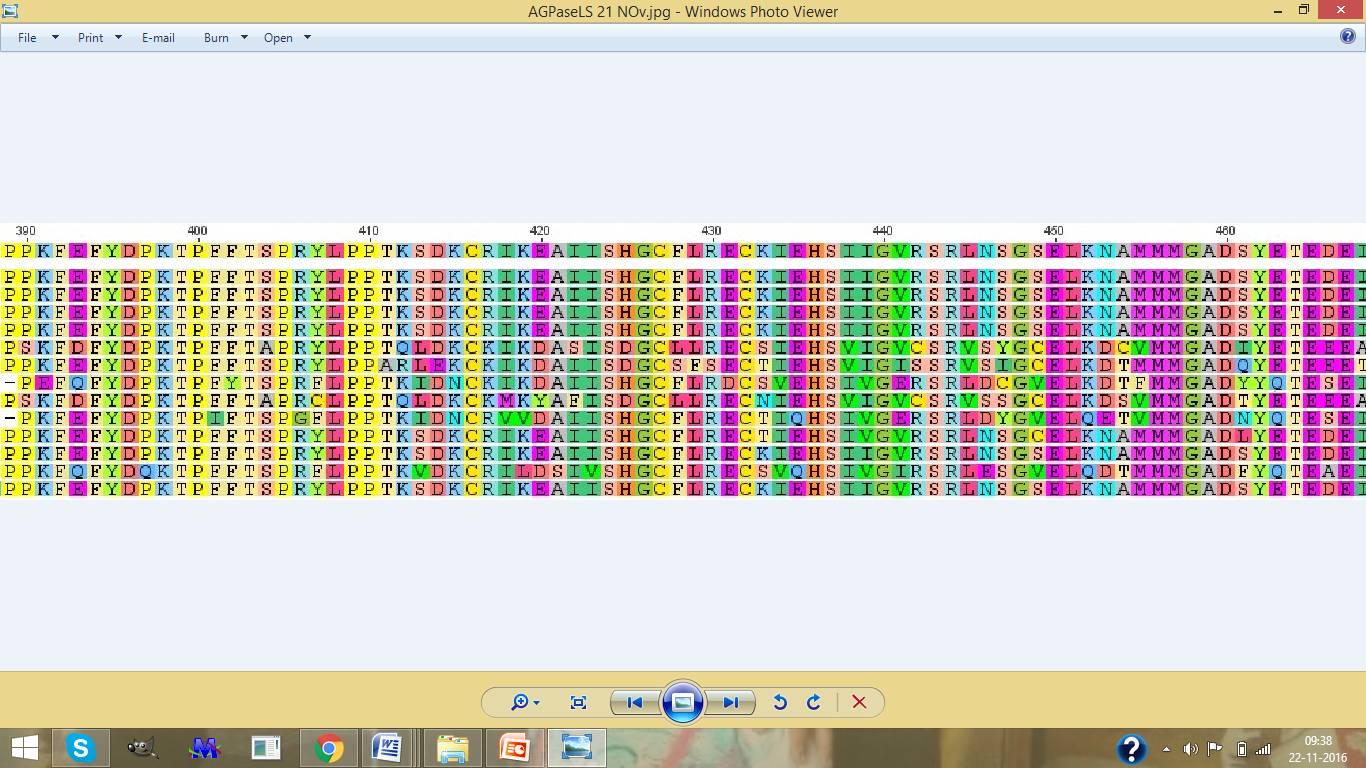


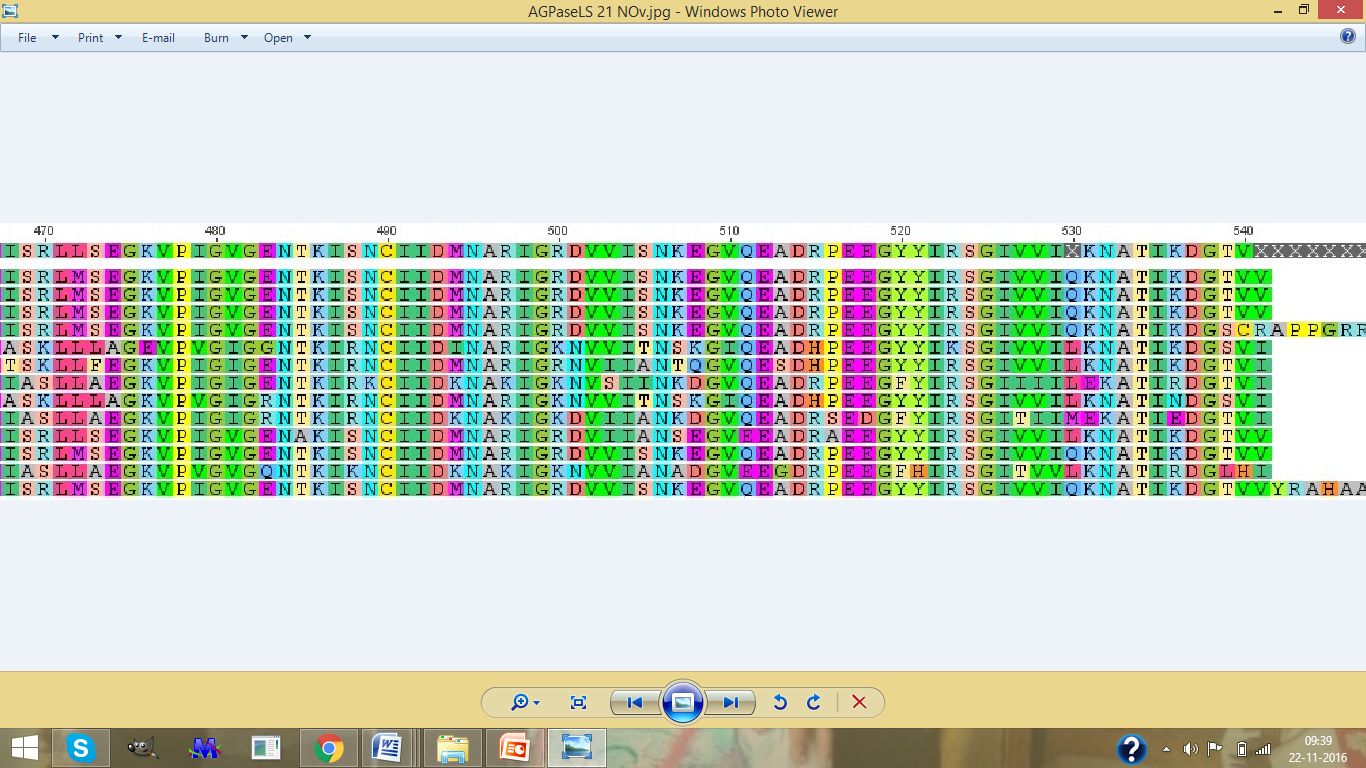


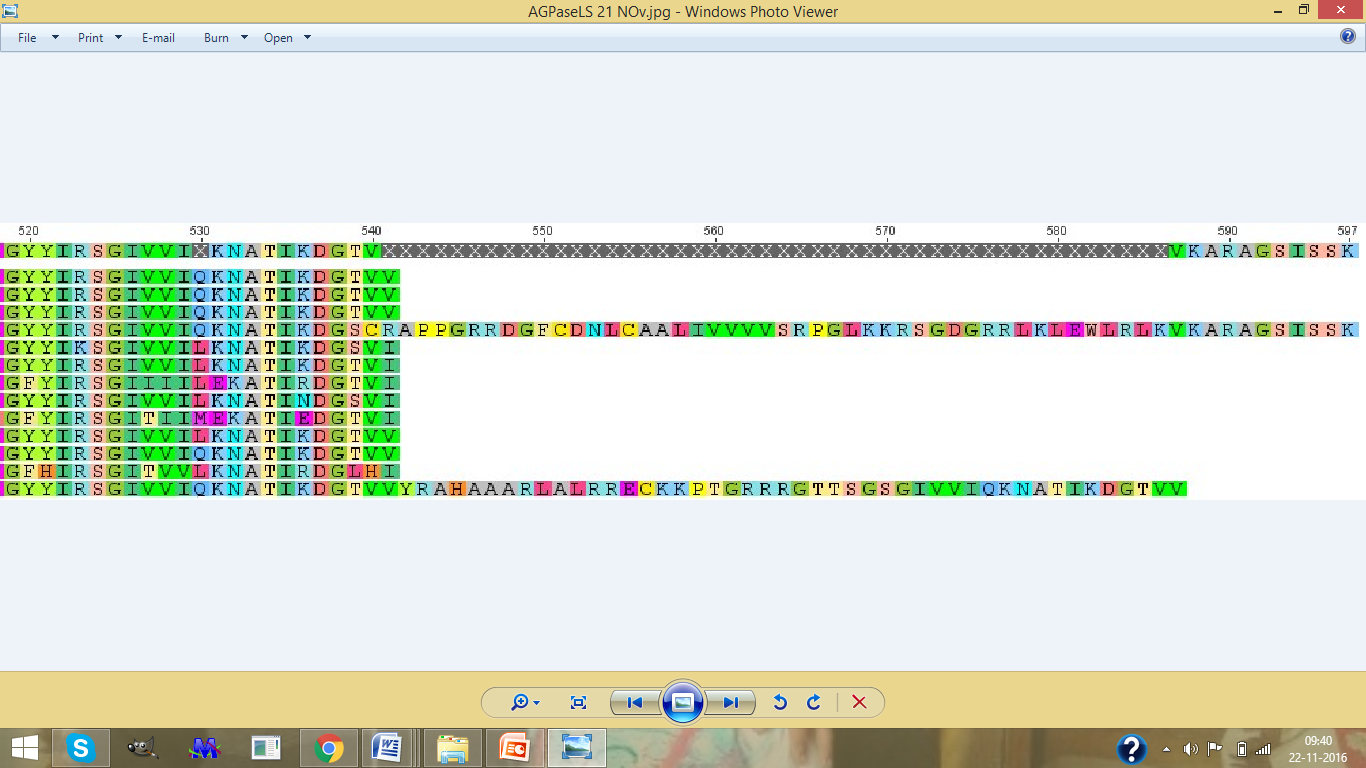

Supplement: Supplementary file 15 [file Presentation1.ZIP › Supplementary Figures/Supplementary Figures/Supplementary Figure 4.docx]

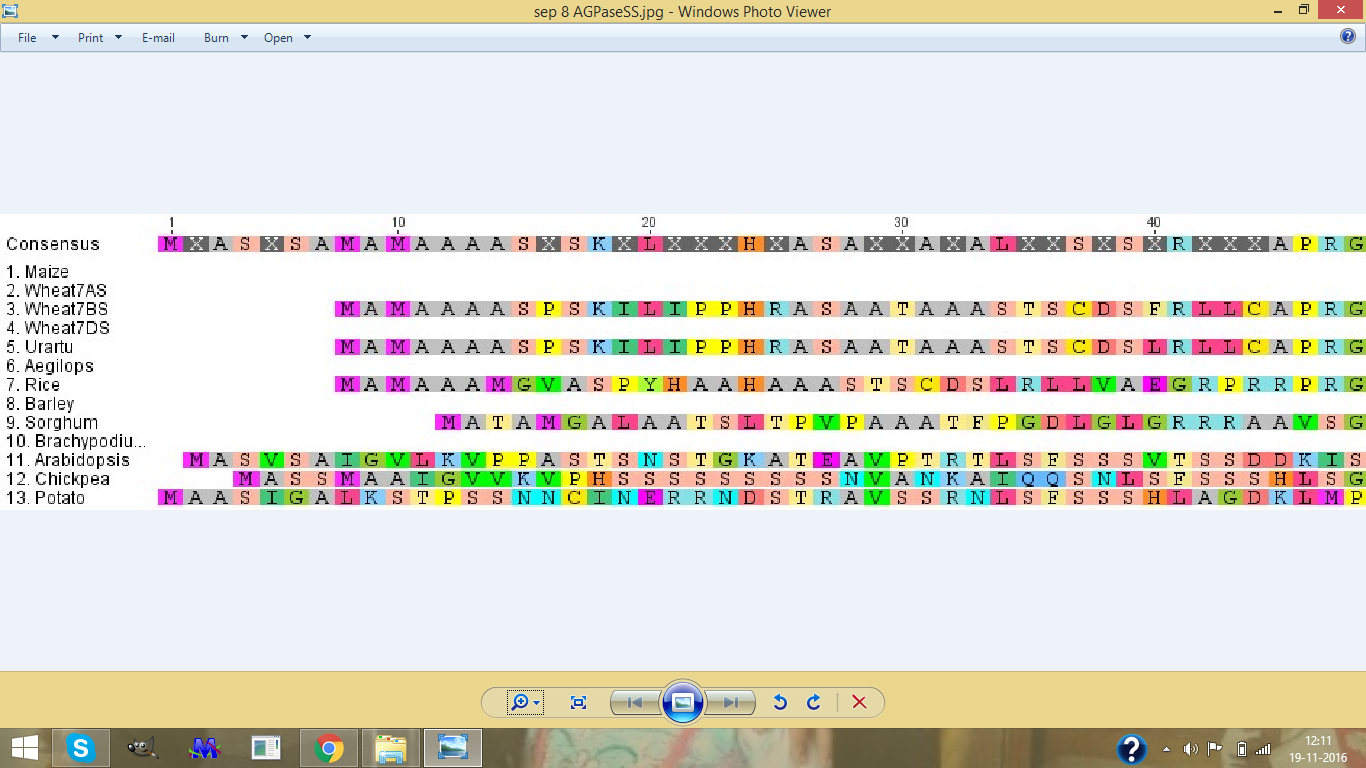


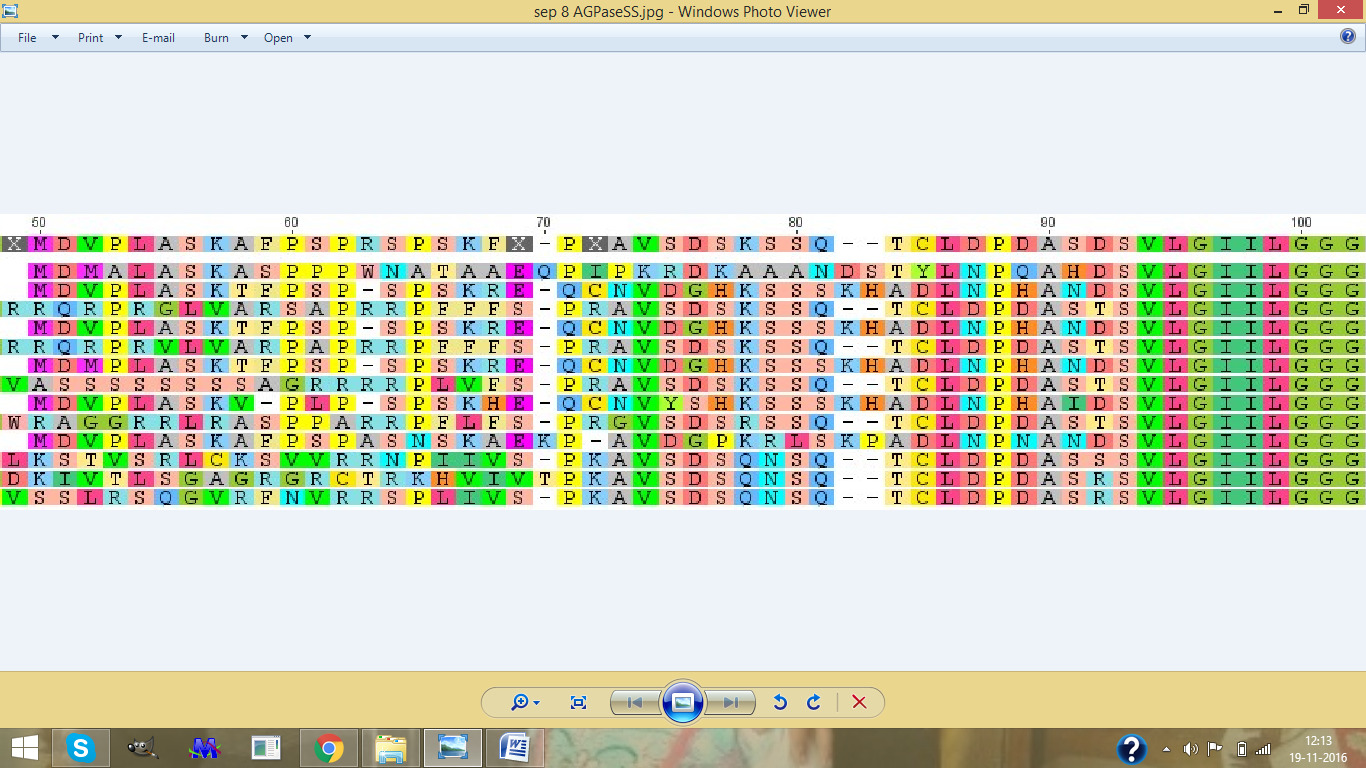


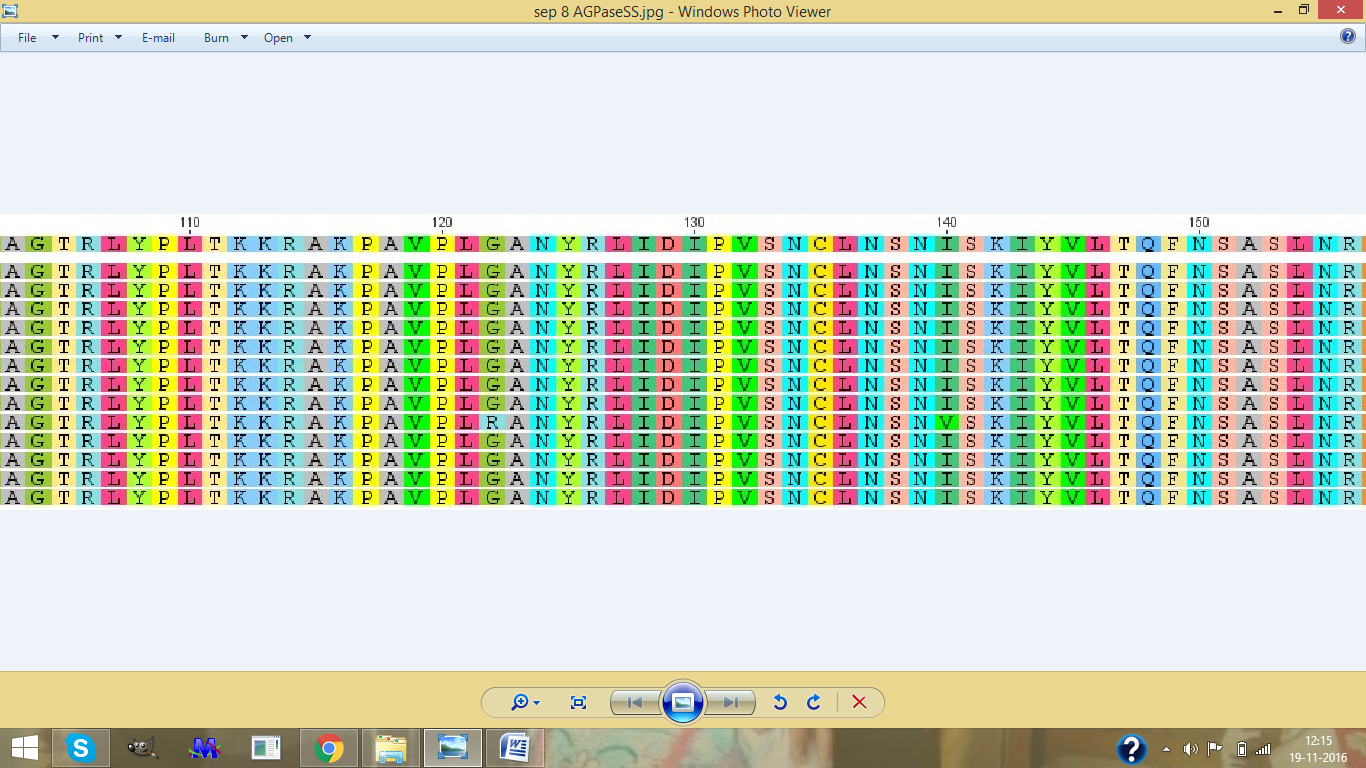


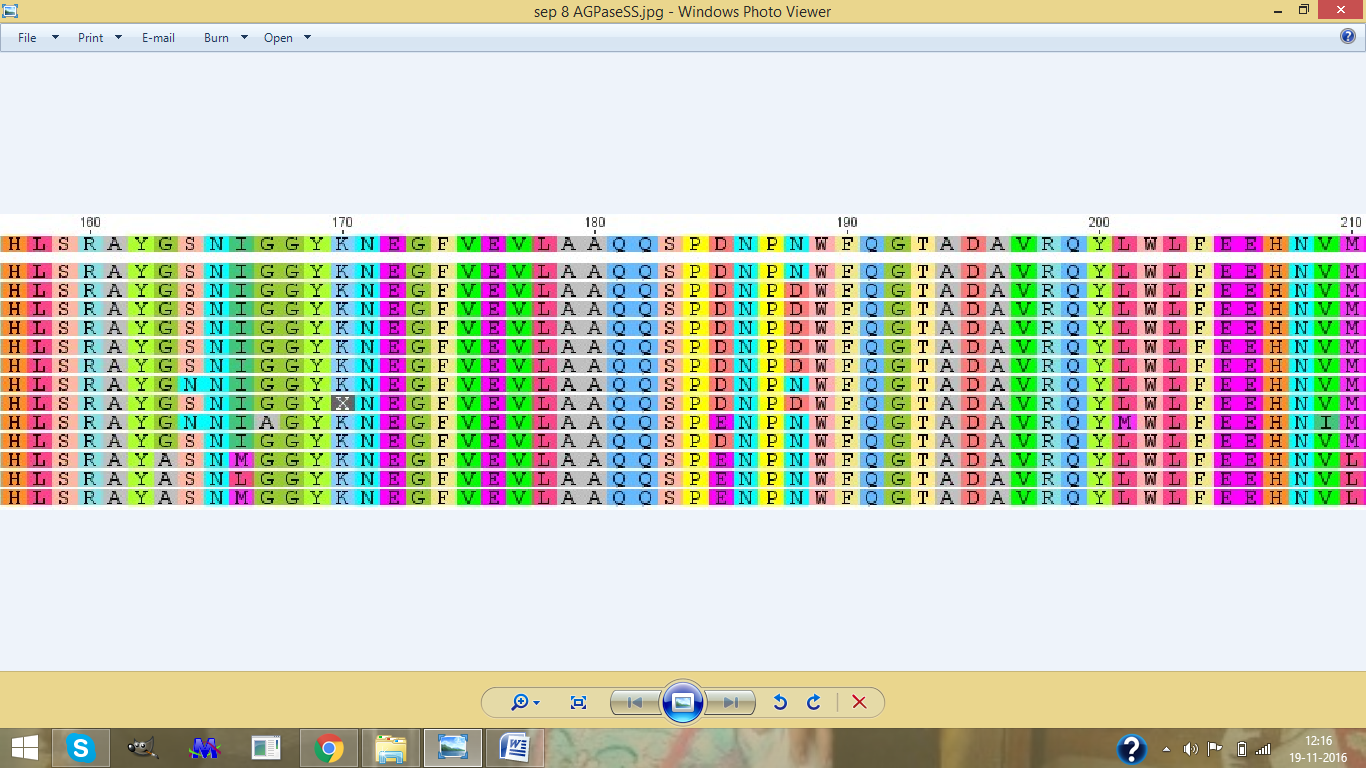


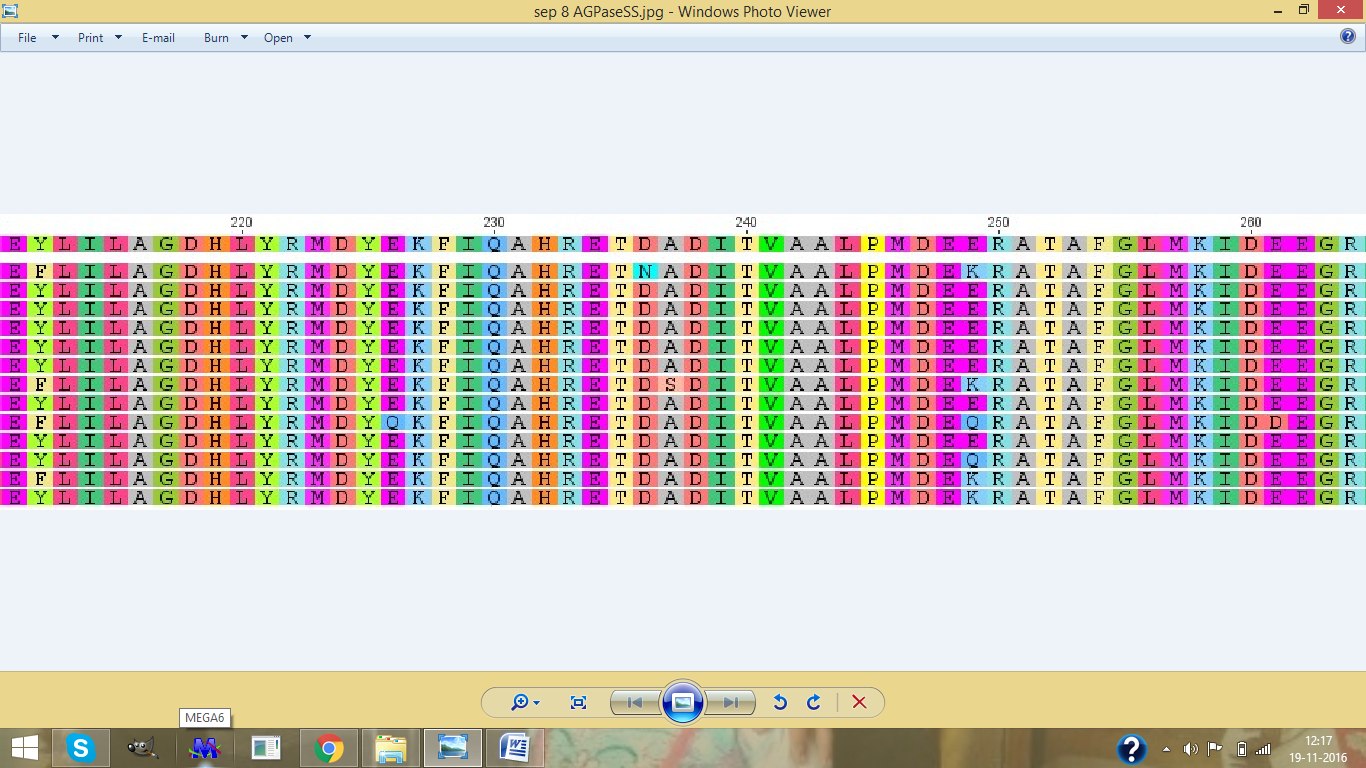


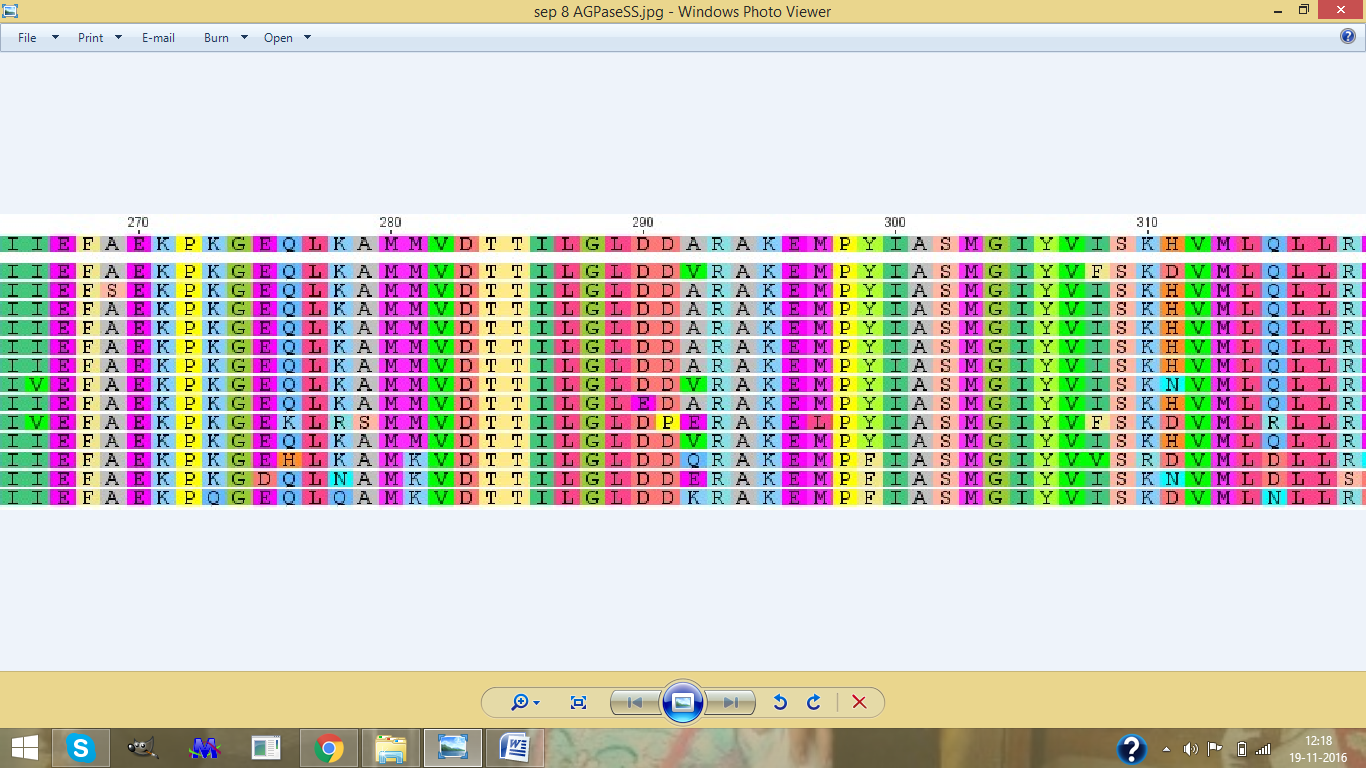


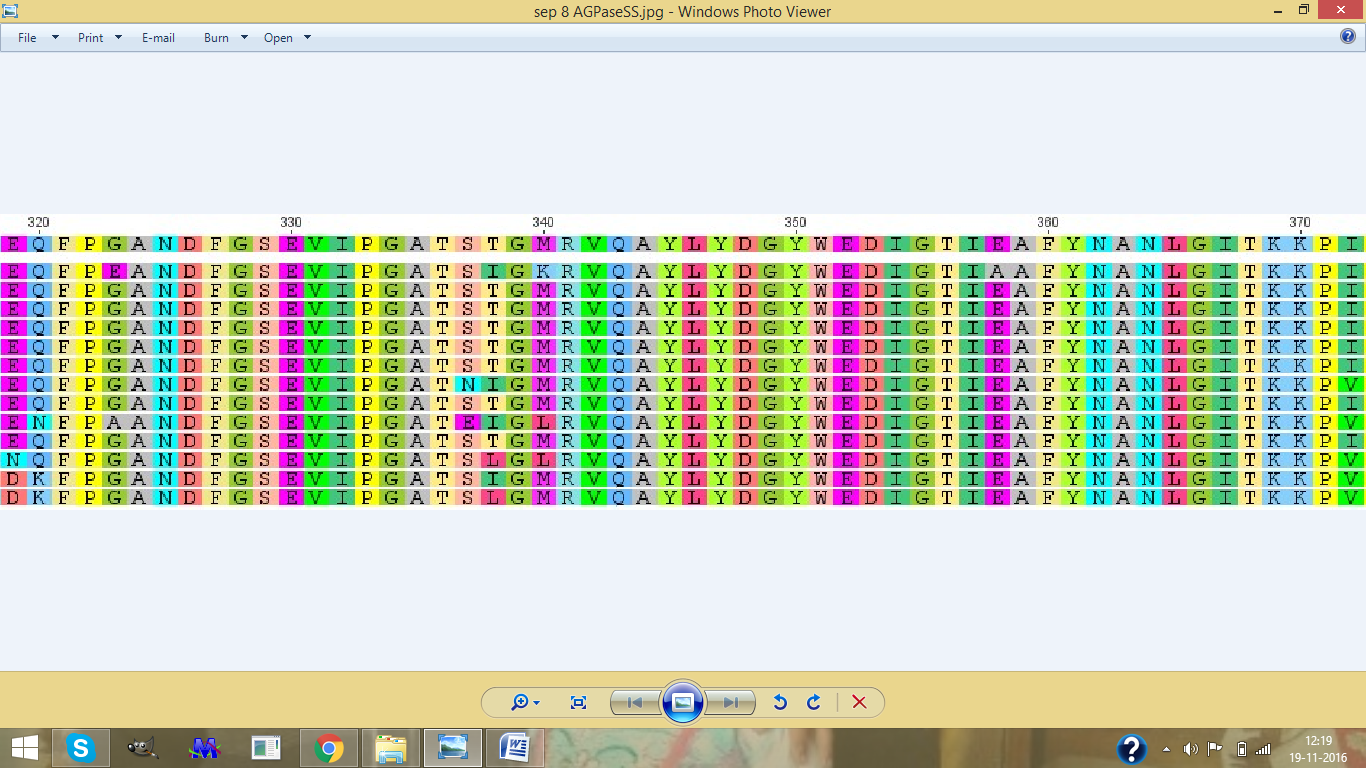


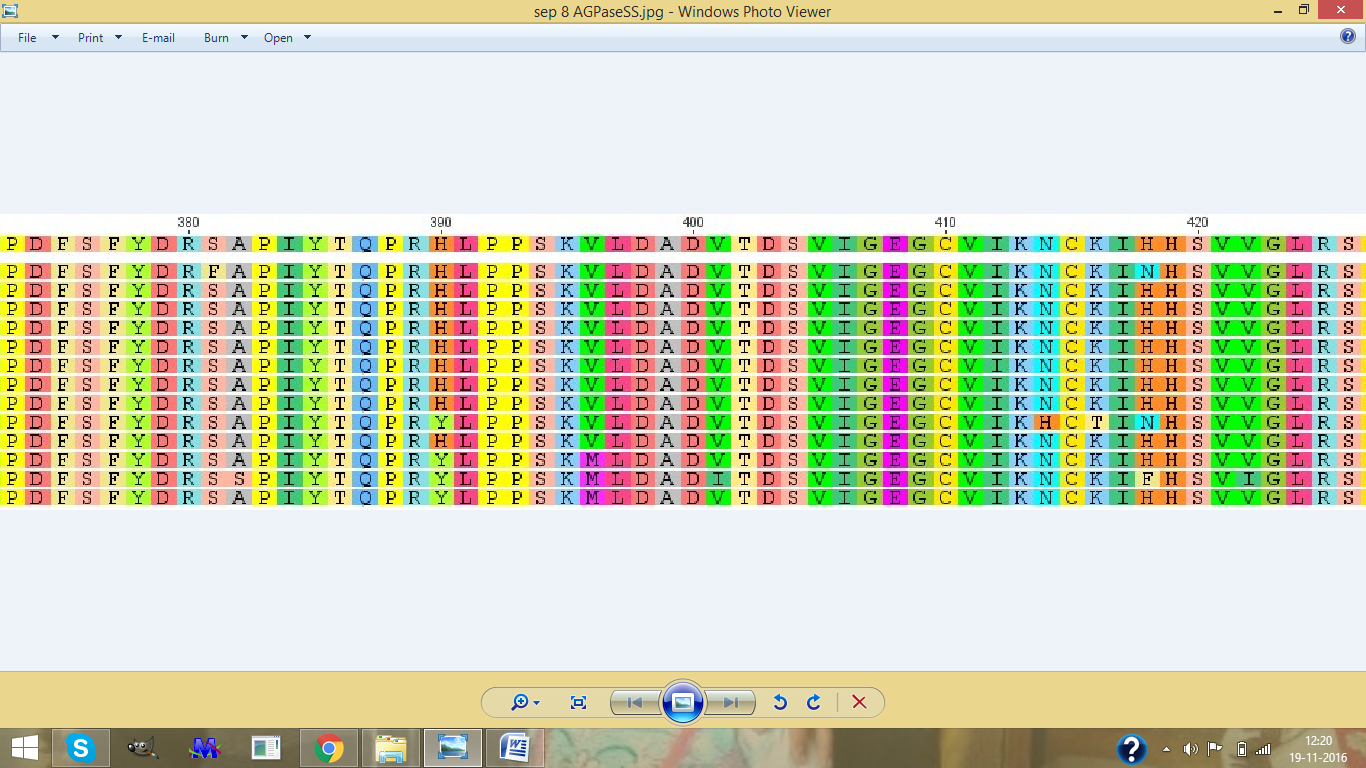


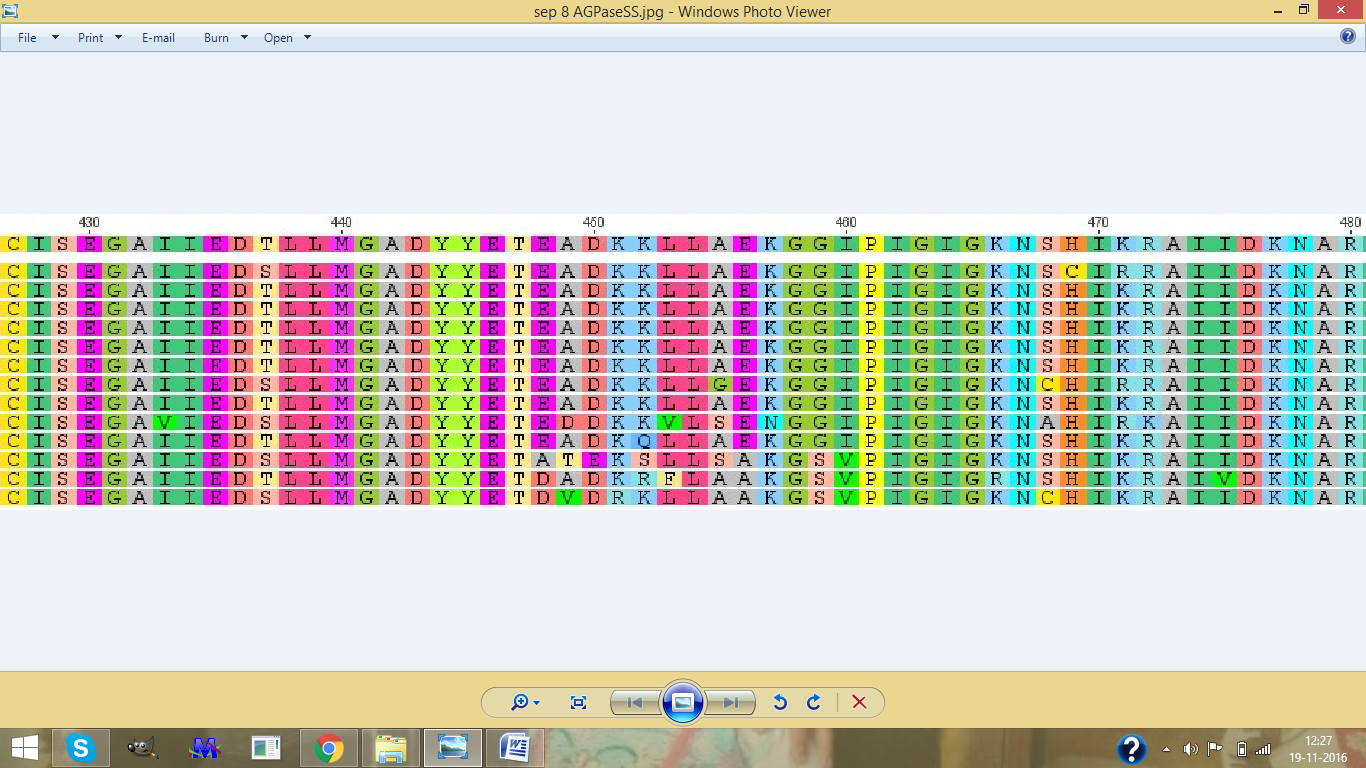


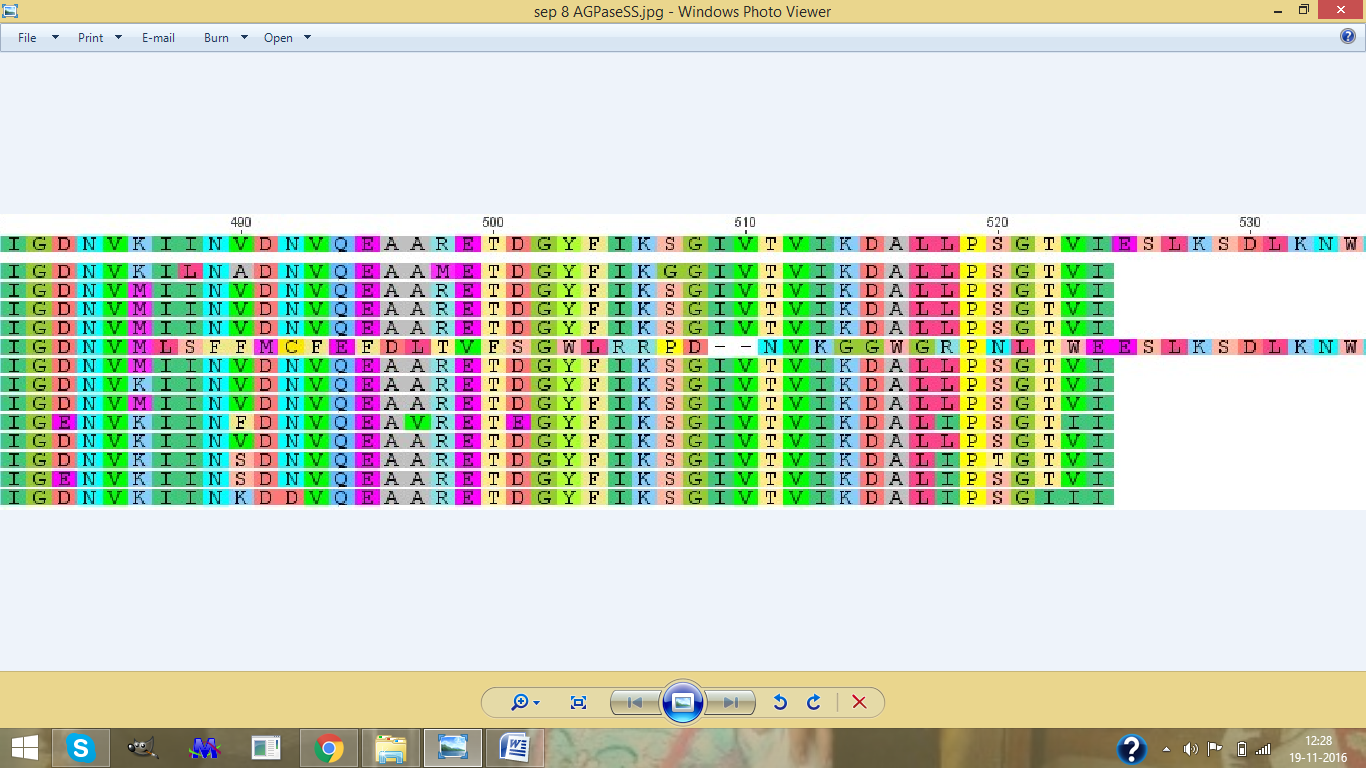


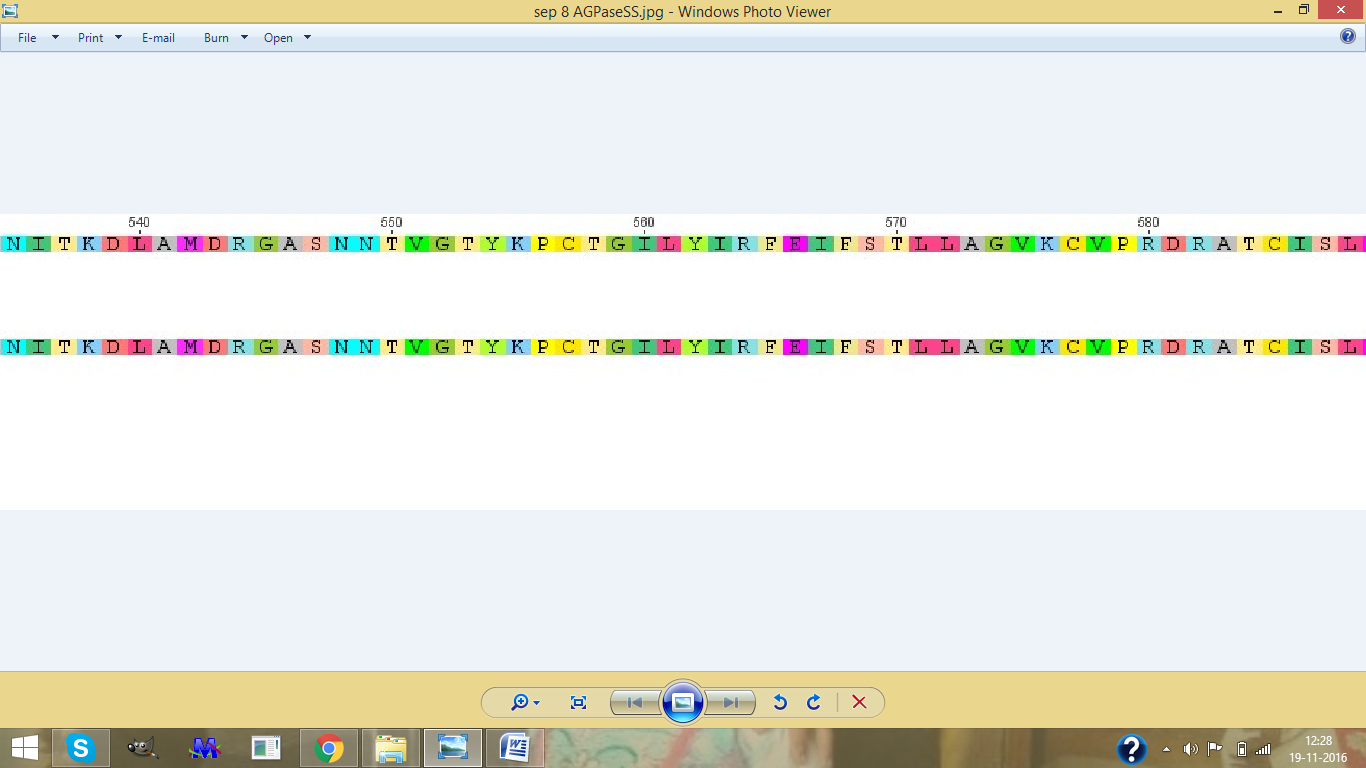


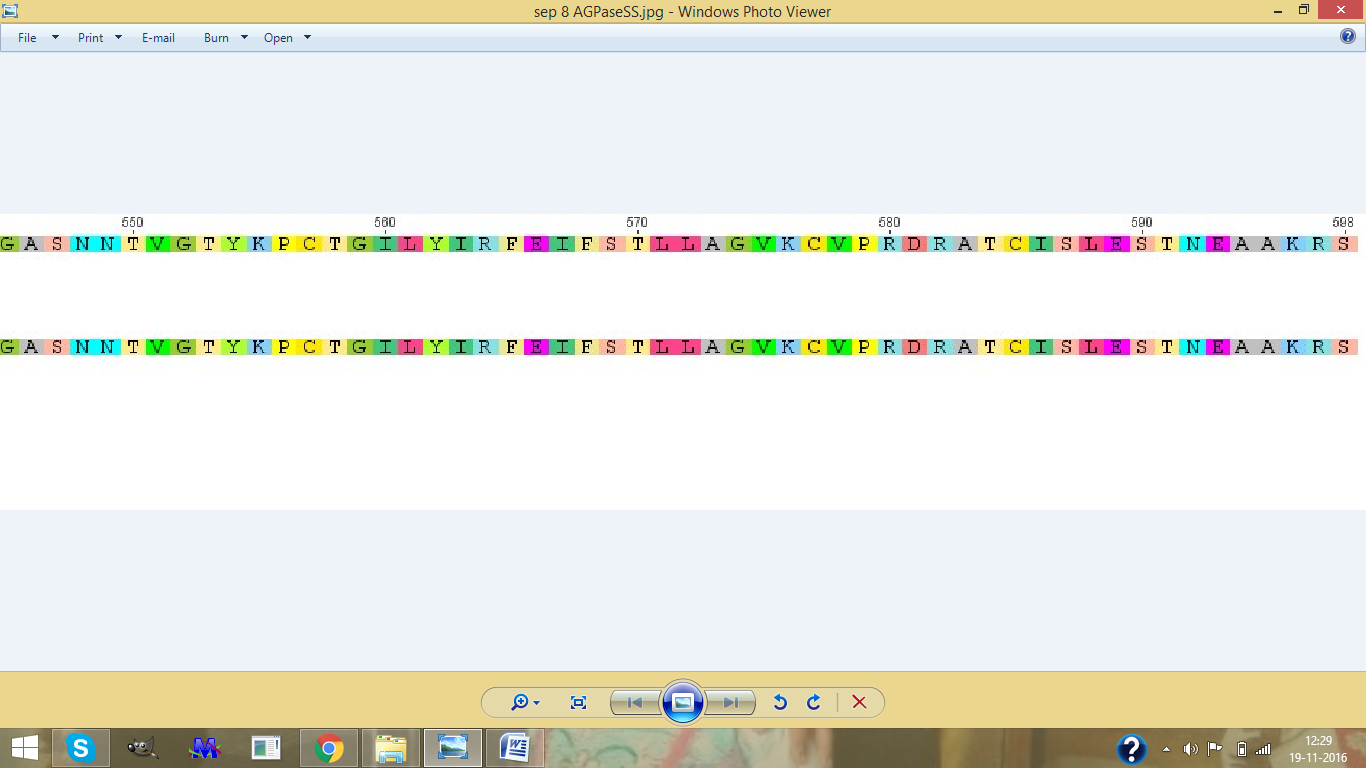

Supplement: Supplementary file 15 [file Presentation1.ZIP › Supplementary Figures/Supplementary Figures/Supplementary Figure 5.docx]

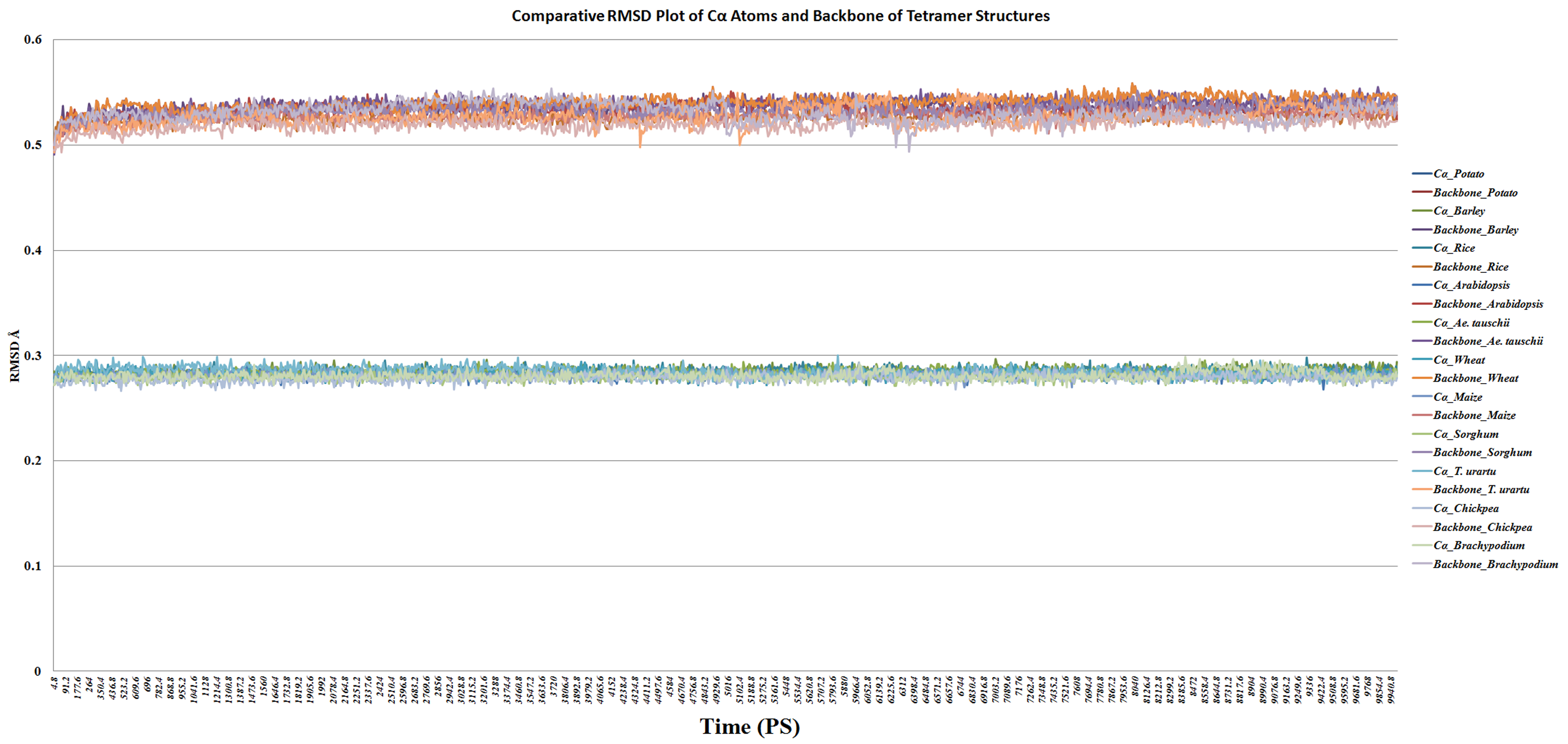

Supplement: Supplementary file 15 [file Presentation1.ZIP › Supplementary Figures/Supplementary Figures/Supplementary Figure 6.tif]

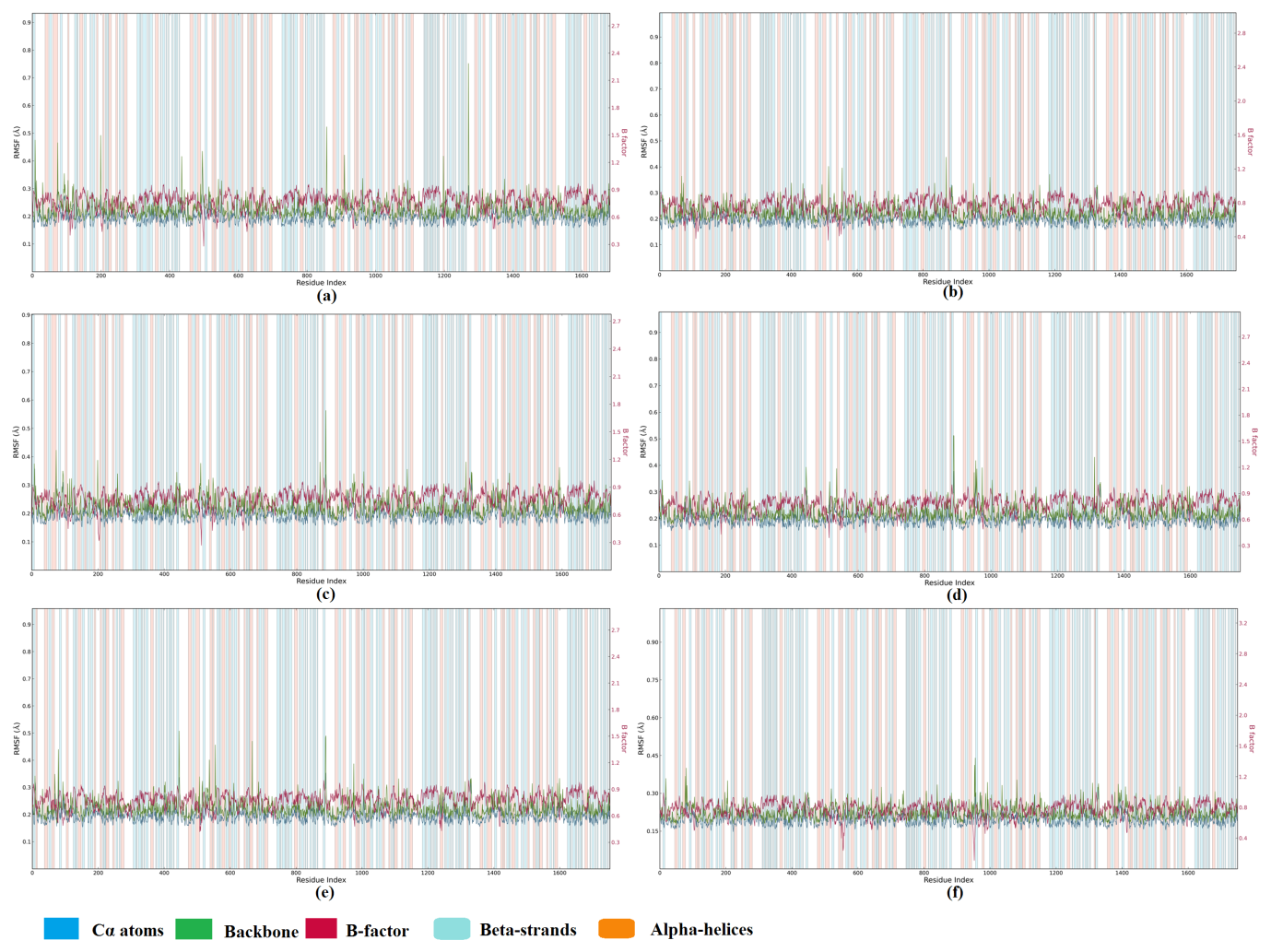

Supplement: Supplementary file 15 [file Presentation1.ZIP › Supplementary Figures/Supplementary Figures/Supplementary Figure 7A.docx]

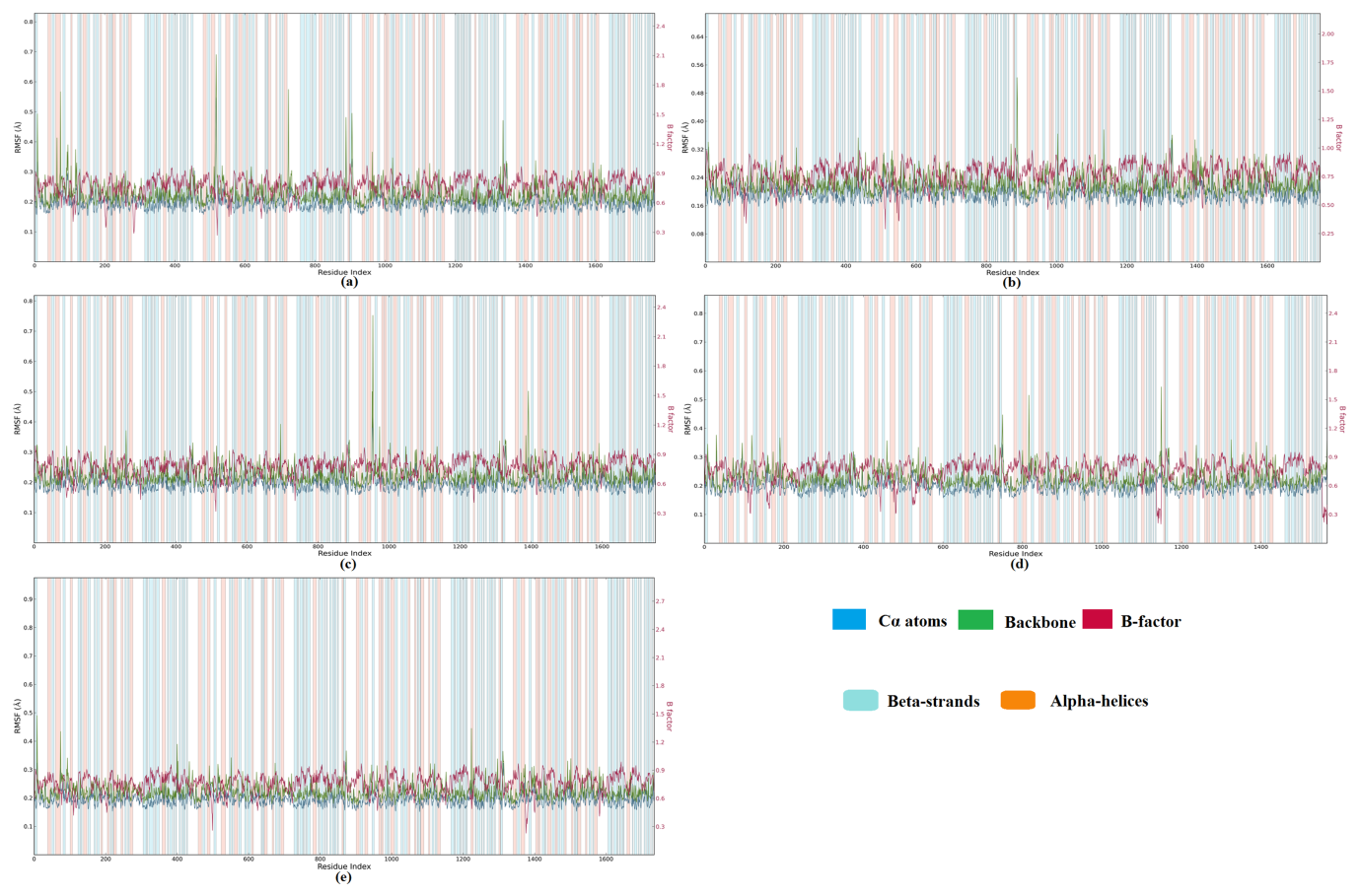

Supplement: Supplementary file 15 [file Presentation1.ZIP › Supplementary Figures/Supplementary Figures/Supplementary Figure 7B.docx]

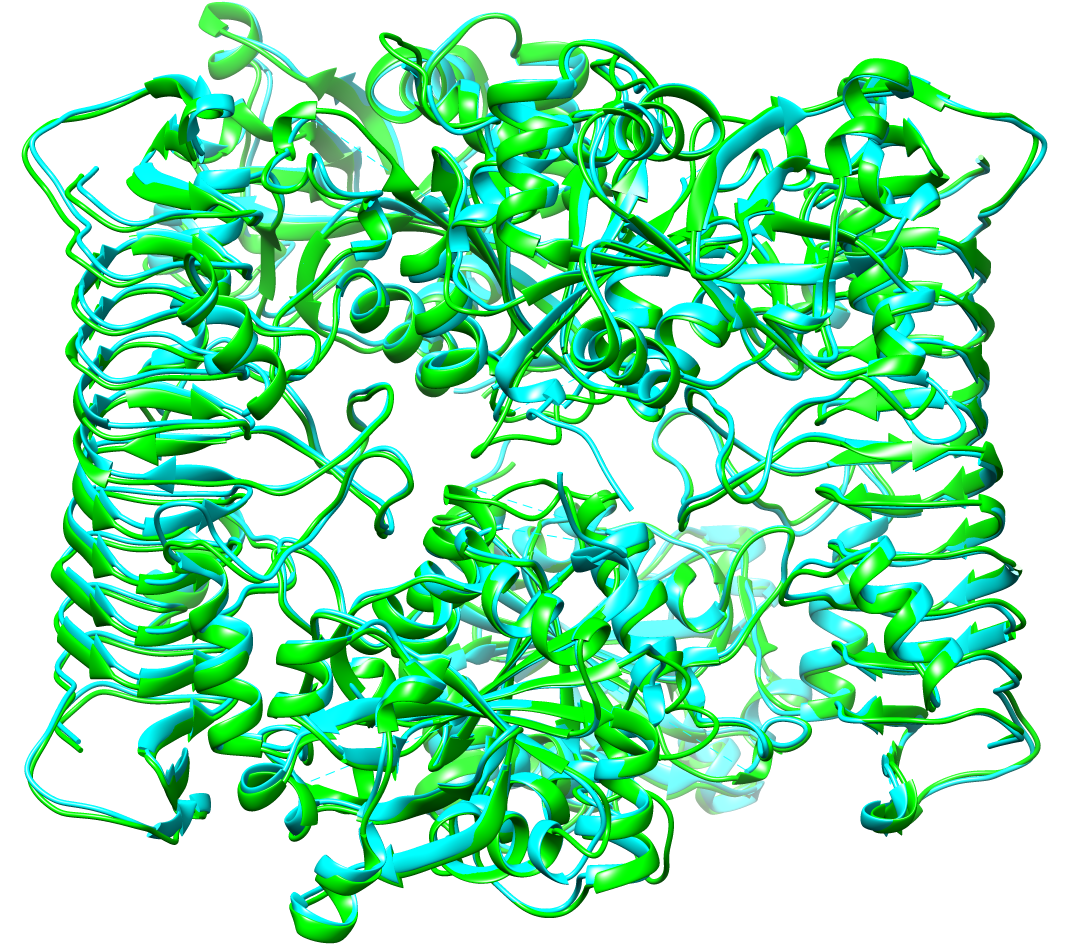

Supplement: Supplementary file 15 [file Presentation1.ZIP › Supplementary Figures/Supplementary Figures/Supplementary Figure 8.tif]

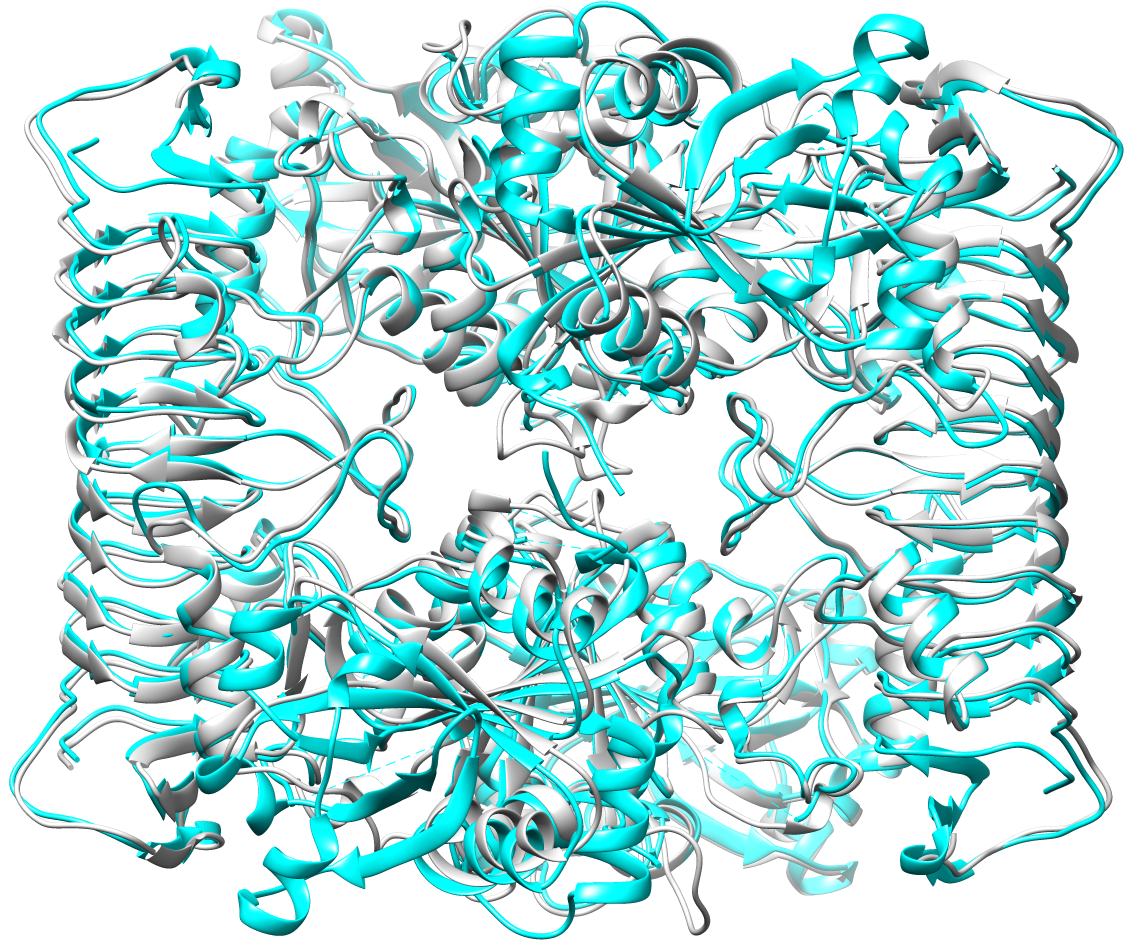

Supplement: Supplementary file 15 [file Presentation1.ZIP › Supplementary Figures/Supplementary Figures/Supplementary Figure 9.tif]
